# Modeling adsorption of brominated, chlorinated and mixed bromo/chloro-dibenzo-*p*-dioxins on C_60_ fullerene using Nano-QSPR

**DOI:** 10.3762/bjnano.8.78

**Published:** 2017-03-31

**Authors:** Piotr Urbaszek, Agnieszka Gajewicz, Celina Sikorska, Maciej Haranczyk, Tomasz Puzyn

**Affiliations:** 1 Laboratory of Environmental Chemometrics, Faculty of Chemistry, University of Gdańsk, Wita Stwosza 63, 80-308 Gdańsk, Poland; 2 Laboratory of Molecular Modeling, Faculty of Chemistry, University of Gdańsk, Wita Stwosza 63, 80-308 Gdańsk, Poland; 3 IMDEA Materials Institute, C/Eric Kandel 2, 28906 Getafe, Madrid, Spain

**Keywords:** brominated, chlorinated, dioxins, fullerenes, QSPR, sorption

## Abstract

Many technological implementations in the field of nanotechnology have involved carbon nanomaterials, including fullerenes such as the buckminsterfullerene, C_60_. The unprecedented properties of such organic nanomaterials (in particular their large surface area) gained extensive attention for their potential use as organic pollutant sorbents. Sorption interactions can be very hazardous and useful at the same time. This work investigates the influence of halogenation by bromine and/or chlorine in dibenzo-*p*-dioxins on their sorption ability on the C_60_ fullerene surface. Halogenated dibenzo-*p*-dioxins (PXDDs, where X = Br or Cl) are ever-present in the environment and accidently produced in many technological processes in only approximately known quantities. If all combinatorial Br and/or Cl dioxin substitution possibilities are present in the environment, the experimental characterization and investigation of sorbent effectiveness is more than difficult. In this work, we have developed a quantitative structure–property relationship (QSPR) model (R^2^ = 0.998), predicting the adsorption energy [kcal/mol] for 1,701 PXDDs adsorbed on C_60_ (PXDD@C_60_). Based on the QSPR model reported herein, we concluded that the lowest energy PXDD@C_60_ complexes are those that the World Health Organization (WHO) considers to be less dangerous with respect to the aryl hydrocarbon receptor (AhR) toxicity mechanism. Therefore, the effectiveness of fullerenes as sorbent agents may be underestimated as sorption could be less effective for toxic congeners than previously believed.

## Introduction

### Dioxin congeners are present and dangerous

Studies on chlorinated dibenzo-*p*-dioxins (PCDDs) as representatives of persistent organic pollutants (POPs) [1] are an important area of the environmental sciences and scientific research [2–5]. PCDDs are usually represented by 2,3,7,8-tetrachloro dibenzo-*p*-dioxin (TCDD), considered as one of the most dangerous and toxic for living organisms upon long-term exposure [6]. Previous studies have shown that other PXDDs (halogenated-brominated and/or chlorinated dioxins, where X = Br or Cl) can also cause toxic effects [7] and induce diverse enzymes and receptors such as aryl hydrocarbon hydroxylase (AHH) and 7-ethoxyresorufin-O-deethylase (EROD) [8]. Most of the toxic effects caused by dioxins are thought to be mediated through a specific protein complex known as the aryl hydrocarbon receptor (AhR) [9]. To interact with AhR, the dioxin structure must penetrate into the cell. This task is easiest for dioxins with symmetrically substituted halogen atoms, such as TCDD. Inside the cell it interacts with the AhR receptor, proteins, and finally, by entering the nucleus, it reacts with the so-called dioxin responsive element (DRE) region on the mRNA surface and causes errors in the translation process and synthesis of new proteins. Most studies are focused on chlorinated dioxins, but brominated dioxins can also be found in environmental samples [7]. Furthermore, in some cases, brominated dioxins show an even higher AhR receptor binding affinity than 2,3,7,8-TCDD [6–7,10]. Because of natural processes occurring in the environment, the total amount of chlorinated PXDD derivatives is constantly decreasing, while the amount of brominated and mixed congeners (molecules based on the same carbon skeleton differing by the number and type of substituents) is increasing [11].

### Fullerene C_60_ – opportunities and risks of possible surface interactions

Fullerene C_60_ [12–14], discovered in 1985, has a soccer ball-like structure [15] with a chemical structure representative of carbon nanostructures. Its unique properties and shape make C_60_ and its derivatives promising candidates for various applications, including sorbents, cancer therapeutics, drug delivery systems, computer sensors, etc. [16–19]. With the further development of nanotechnology, C_60_ will be produced and used in large amounts. Over time, fullerene structures will be found in the environment more often and in higher concentrations.

Aromatic structures render fullerenes as good acceptors of π-electrons. On the other hand, aromatic systems like halogenated dioxins are classified as π-donors [20]. Recent studies have proved that halogens, such as bromine or chlorine, have a more positive region on the surface opposite to the X–C bond direction as well as an equatorial belt of negative potential, so that they can display different properties depending on the angle of approach [21]. Regarding one of the toxicity mechanisms for nanoparticles proposed at the NATO Advance Research Workshop, dioxin–fullerene interactions and complex formation can be dangerous because of the ability of nanoparticles like fullerenes to penetrate biological barriers and act as a vector, transferring dioxins or other pollutants inside the cells [22].

The potential applications of C_60_ as smoke filters or air-cleaning agents are examples where it may be employed to improve environmental conditions [13,18]. On the other hand, if the sorption interactions can occur spontaneously in the environment, they may bring as much hope as they do risk [23–26].

### Objective

The possibility of surface interactions between fullerenes and organic compounds has raised the question: How many halogenated PXDDs congeners will create a PXDD@C_60_ complex based on weak π–π interactions, and what is the influence of halogen substitution of dioxin congeners in these interactions?

The main goal of this study was to calculate the adsorption energy for the representative subset of dioxin congeners and to develop a model to predict the energy for a large subset of structurally similar compounds. At the same time, the goal was to demonstrate that it is possible to predict the influence of the substitution pattern (i.e., type, number, and location of halogen substituents) in dioxin molecules on the final adsorption energy of the complex by using in silico methods.

Our model presented here may provide important information in designing new fullerene applications and assessing the risk of those interactions according to the differences in toxicity caused by the number and type of substitution. The investigations have been performed with quantitative structure–property relationship modelling for nanomaterials (Nano-QSPR) – a method of defining a mathematical function that connects the structure of the investigated nanomaterial (fullerene) and the POPs (dioxin) with a modeled property (energy of the PXDD@C_60_ complex). It is a computational technique that, to the best of our knowledge, is the first published example of the use of Nano-QSPR to predict interactions between fullerenes and numerous organic pollutants.

## Results

### Nano-QSPR model

Based on the adsorption energy values (Δ*E*_ads_) for 32 Br/Cl dibenzo-*p*-dioxin congeners adsorbed on a C_60_ fullerene surface and carefully selected structural descriptors, we developed a Nano-QSPR model, employing a hybrid genetic algorithm, partial least squares linear regression (GA-PLS), as the modeling method. The developed Nano-QSPR model utilizes only four descriptors for predicting the adsorption energy values for 1,669 PXDD@C_60_ materials as follows:

[1]
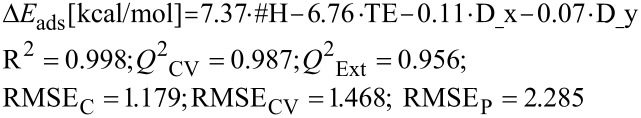


where #H is the number of hydrogen atoms in the dioxin molecule, TE is the total energy of the molecule, and D_x and D_y are the dipole moments of dioxin molecule along the *x* and *y* axis, respectively. More detailed information about the statistical description of the obtained model is available in Supporting Information File 1.

### Applicability domain and OECD guidelines

The developed Nano-QSPR model has been comprehensively validated according to the Organization for Economic Co-operation and Development (OECD) QSAR validation recommendations [27] and fulfills all the validation criteria. The presented model has a well-defined endpoint (Δ*E*_ads_ - adsorption energy of a C_60_@PXDD complex) and well-known algorithms (GA-PLS). According to OECD guidelines, it is recommended to define its applicability domain (AD). This is a theoretical space for which the predictions are most reliable and applicable. The applicability domain for models based on theoretical data can be verified by use of the leverage values [28–29] and the values predicted by the model presented on the same plot (so-called Insubria graph or Insubria plot) [30]. This approach allows for verification of the AD for training, validation and prediction sets at once. The leverage value represents the distance of a compound from the training set (TS) centroid. Given this, it is possible to determine whether a predicted model response is an effect of interpolation (compound within the AD) or extrapolation (compound outside the AD) of the model [31–32]. The Insubria graph is available in Supporting Information File 1 (Figure S1) and proves that all of the congeners from training and validation sets are located in the space of applicability domain (AD). Moreover, 1,563 congeners out of 1,669 from the prediction set lie in the space of AD. Only 84 congeners (4.94% of all 1,701 dioxin congeners) have leverage values higher than critical h* = 0.625, and for those C_60_@PXDDs, the predicted Δ*E*_ads_ may be less reliable. We consider this to be a very good result for the Nano-QSPR model based on 24 congeners in the training set, 8 congeners in the validation set and 1,669 in the prediction set.

The developed Nano-QSPR model was internally and externally validated [28]. The leave-one-out cross-validation (LOO method) was the algorithm chosen for internal validation and allowed the model's robustness to be calculated – *Q*^2^_CV_ (cross-validation coefficient) and RMSE_CV_ (root mean square error of cross validation). The external validation was performed with an independent set of congeners (8 dioxins not used during model development). With the external validation, we have calculated the predictive power of the model defined by *Q*^2^_Ext_ (0.956), the external validation coefficient, and RMSE_p_ (2.285), the root mean square error of prediction [33]. The selection of a final number of latent vectors was based on the cross-validation results (the lowest value of the root mean square error of cross validation, RMSE_CV_).

**Figure 1:**
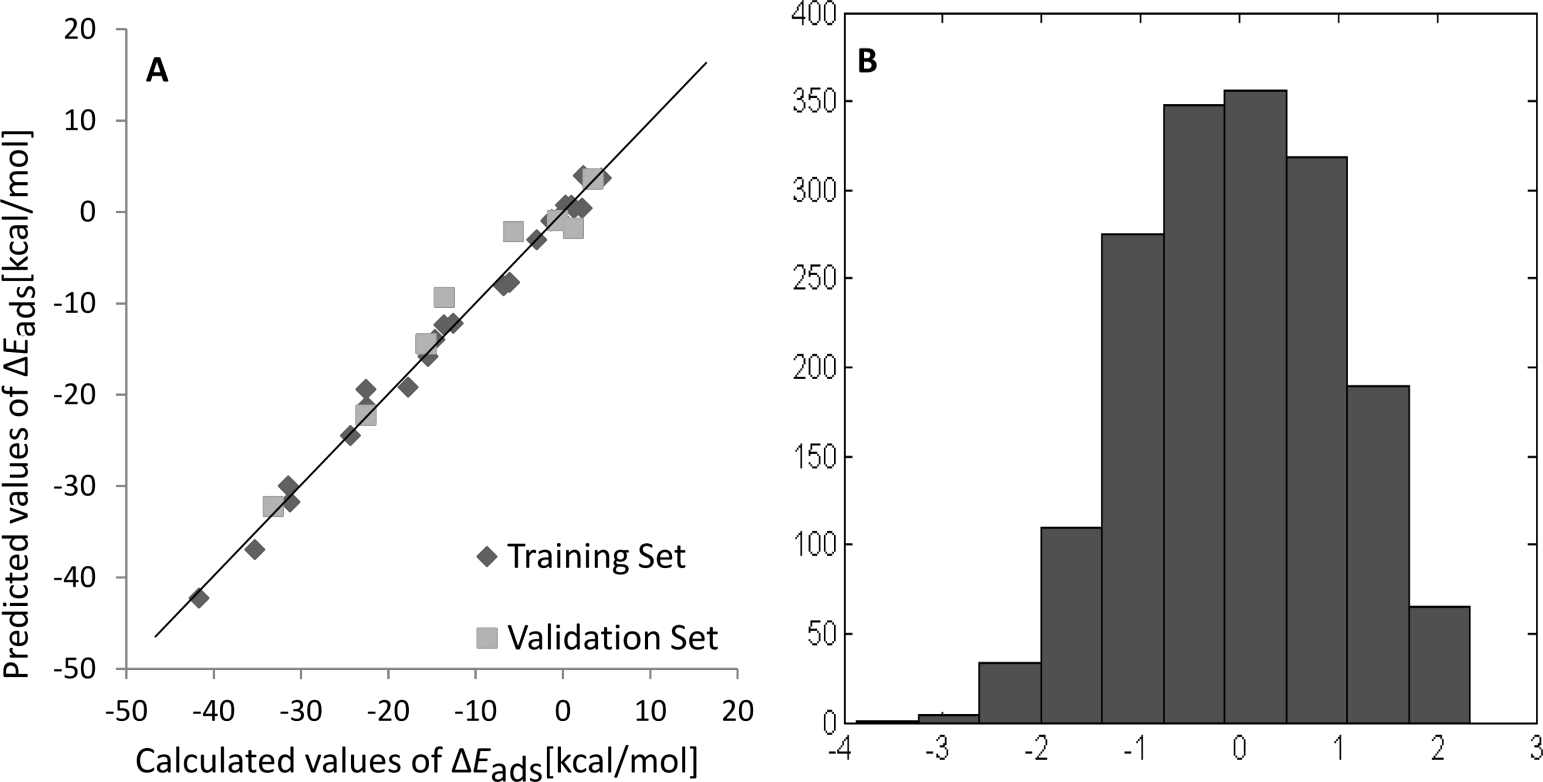
a) Plot of calculated and predicted values of Δ*E*_ads_ energy. b) Histogram of calculated and predicted values of Δ*E*_ads_.

Four latent vectors (LVs), as a set of 4, were selected by the GA descriptors: #H, TE, D_x and D_y together explained 100% of the variance (58.08% + 40.42% + 1.30% + 0.20%) of the X-block and 99.22% variance of the Y-block (40.63% + 1.70% + 22.73% + 34.16%). According to OECD guidelines, when R^2^, *Q*^2^_CV_, and *Q*^2^_Ext_ values are close to 1, and RMSE_C_, RMSE_CV_ and RMSE_P_ are as low as possible, the developed model can be considered as robust, well-fitted and having good predictive abilities. The Nano-QSPR model presented in this study fulfills the guidelines given by OECD, which is also shown in Figure 1a (a satisfactory correlation between the calculated and predicted Δ*E*_ads_ values for the training and validation sets). Moreover, the histogram performed for autoscaled *E*_ads_ values for all 1,701 PXDD congeners (Figure 1b) shows that the values have a normal distribution.

### Mechanistic interpretation of the Nano-QSPR model

**Figure 2:**
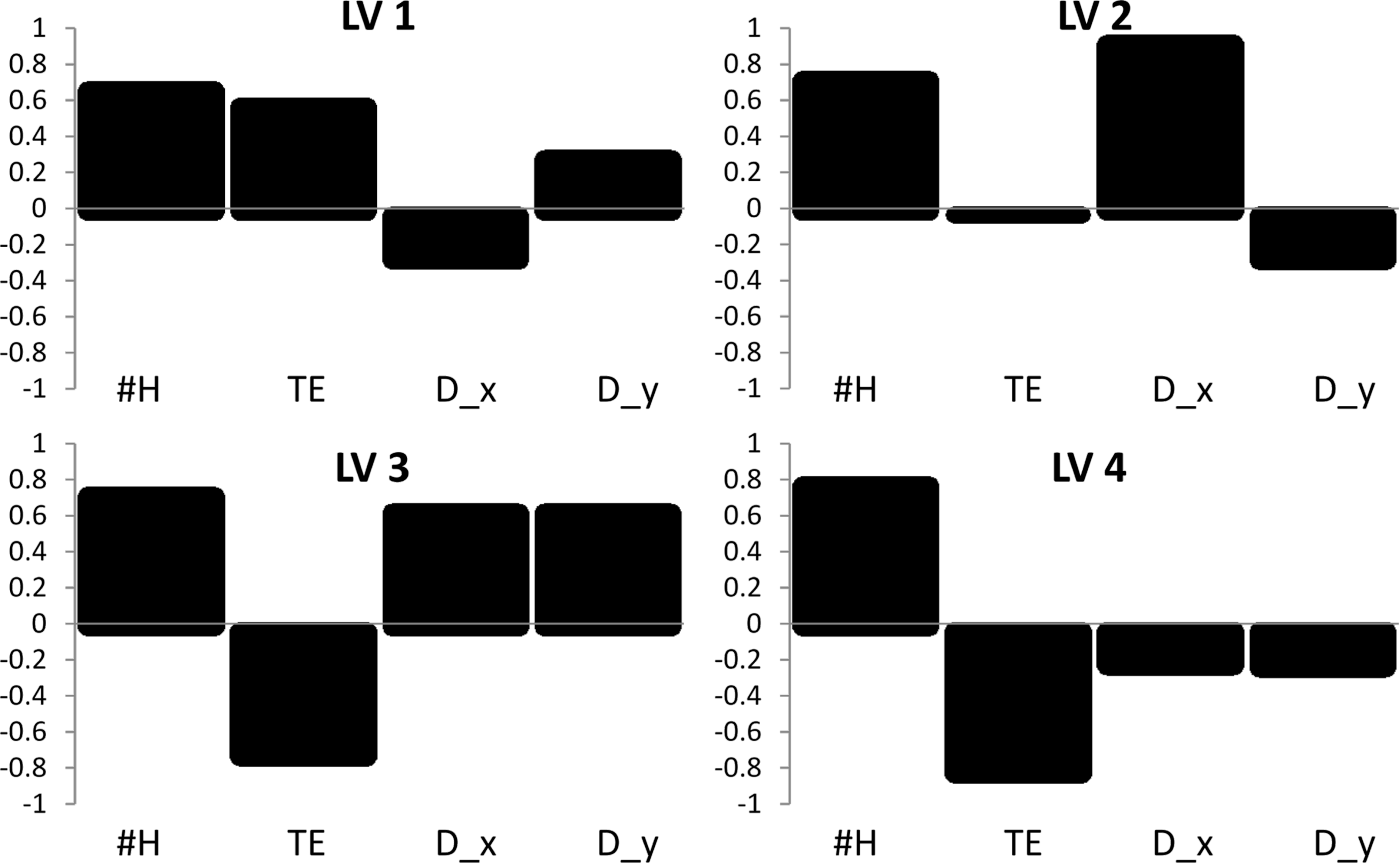
Significance and loading values of individual latent vectors (LVs).

**Figure 3:**
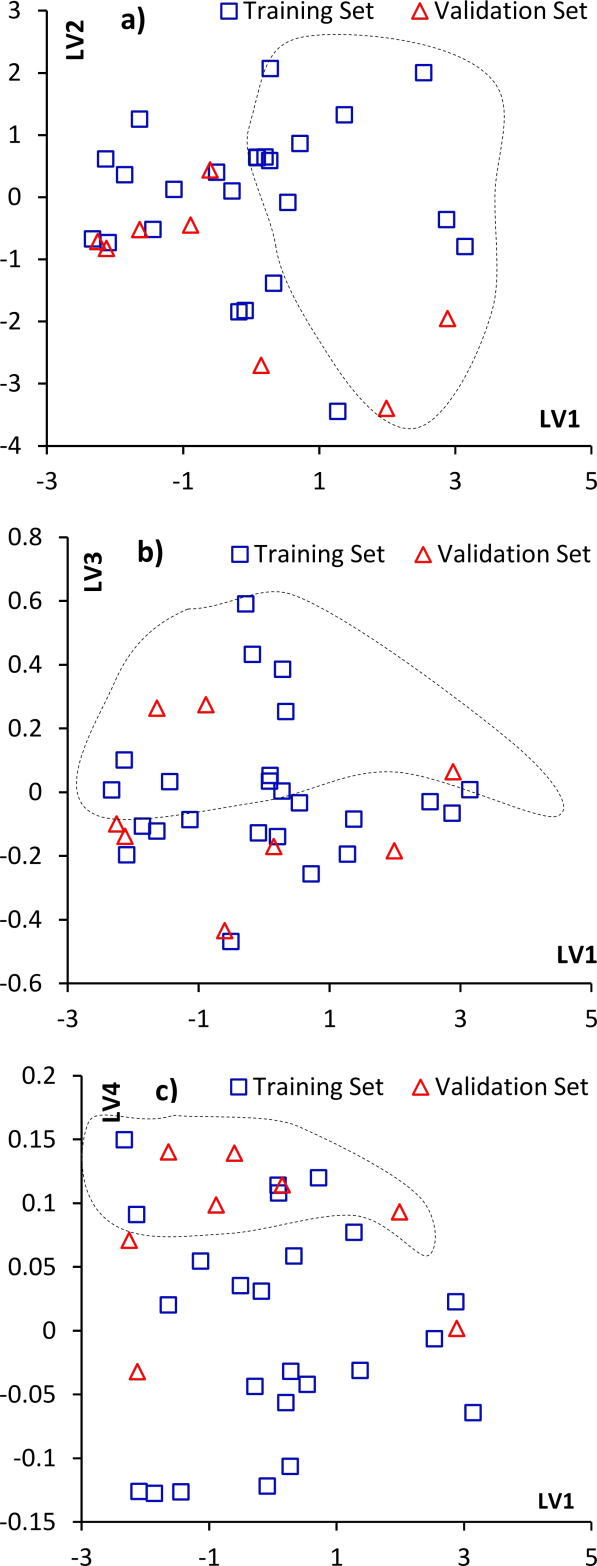
Score plots of dioxin congeners selected for the training (□) and validation (Δ) set in the space of latent vectors. Marked areas are: a) more than 50% of H atoms, and halogenation only in 2,3,7,8 positions for b) unsymmetrical halogen atom (preferably Cl) and c) unsymmetrical substitution (preferably chlorination).

By analyzing scatter plots and loading values of the LVs, it is possible to interpret the obtained model. LV loadings show the contribution of a particular descriptor to a given latent vector (Figure 2), while score plots present training and validation set congeners in the space of LVs (Figure 3).

As presented in Figure 2 and Figure 3a–c, the first latent vector (LV1) is mainly related to the #H, TE, and D_y descriptors. Such a combination suggests that congeners with hydrogen atoms, located along the *y* axis (in positions 1,4,6,9), and halogen atoms, mainly in lateral positions (2,3,7,8), have higher values of adsorption energy. D_y and D_x loadings with opposite signs suggest that higher values of predicted Δ*E*_ads_ will have dioxins substituted by more electro-negative atoms (preferably chlorine than bromine) in nonlateral positions along the *y* axis.

The second latent vector (LV2) (Figure 2, Figure 3a), is a combination of D_x and #H, and is related to those congeners with a high dipole moment along the *x* axis. Congeners mostly substituted in positions 2,3,7,8 and filled with hydrogen atoms in positions 1,4,6,9 will have the highest values of LV2. Because of the differences in the electro-negativity of bromine and chlorine (Br = 2.96, Cl = 3.16, Pauling scale), congeners more chlorinated than brominated would have a higher dipole moment along the *x* axis of dioxin, and in effect, higher predicted values of sorption energies. It should be noted that structures considered as toxic have lateral halogen atoms and will have higher *E*_ads_ values, unless the number of hydrogen atoms are decreased by chlorine or bromine substitution in 1,4,6 or 9 positions.

All four descriptors have a significant contribution in the third latent vector (LV3), but only TE has a negative loading value. As shown in Figure 2 and Figure 3b, LV3 separates congeners with few hydrogen atoms substituted in the dioxin structure, and unsymmetrical halogen substituents located along both the *x* and *y* axis. The halogen atom will preferably be chlorine because of the negative TE contribution in this LV and higher influence of chlorine substitution to the dipole moment of congeners.

As shown in Figure 2 and Figure 3c, the last latent vector (LV4) is a combination of #H and negative TE, with small negative D_x and D_y contributions, which can be interpreted as a complementation of LV3 and separates unsymmetrical substitutions in congeners with a predominance of chlorine atoms.

Since the developed Nano-QSPR model fulfilled all OECD recommendations, including the mechanistic interpretation, we applied the model to predict the adsorption energy Δ*E*_ads_ for the rest of the brominated or/and chlorinated dibenzo-*p*-dioxin congeners. All predicted data with values for particular descriptors from the prediction set are available in Supporting Information File 1, Table S3.

## Discussion

In the literature, there are only very few examples of experimental studies aimed at interactions between fullerenes or other carbon nanomaterials with particles such as proteins [34], porphyrines [35], toxic water pollutants [14], solid phases [36], or other materials [37]. Dibenzo-*p*-dioxins and dibenzofurans produced during incineration of nanomaterials have also been studied [38]. There are also a few studies aimed at using in silico methods, such as semi-empirical or density functional theory (DFT) calculations, for exploring interactions on nanoparticle surfaces [39–41].

Our results provide new knowledge about: i) general sorption interaction mechanisms of dioxin congeners on the C_60_ surface, and ii) applicability of the Nano-QSPR approach for predictions of organic pollutant congeneric groups.

As far as we know, the proposed study is the first attempt to use the Nano-QSPR approach for predictions of the interaction between the congeneric family of pollutants and the C_60_ nanoparticle.

### Toxicity results

In this study, we consider toxicity to be well-described for dioxins and dioxin-like compounds as an aryl hydrocarbon receptor (AhR) interaction mechanism. AhR is a cytosolic transcription factor. Normally, the inactive protein is bound to several co-chaperone proteins. Upon binding to a dioxin structure or dioxin-like compound, the chaperones dissociate, resulting in an AhR translocation into the nucleus and dimerization with aryl hydrocarbon receptor nuclear translocator (ARNT) protein, which leads to interaction with the dioxin responsive element (DRE) of the nucleic acids and synthesis of new proteins or changes in gene transcription [9]. Dioxins have the largest binding affinity to AhR proteins, which are symmetrically substituted in 2,3,7,8 positions. Chlorine or other halogen atoms in positions 2,3,7, and 8 in dioxin structures are essential for toxicity and also prevent the early enzymatic destruction of dioxin. Each additional chlorine in the 2,3,7,8-structure decreases the toxicity according to the AhR mechanism. However, the congeners can still cause a toxic response during enzymatic reactions or oxidation processes inside the organisms.

Less toxic (according to AhR interactions) dioxin congeners will have a halogen substitution in 1,4,6,9 positions and lower dipole moment along the *x* axis. In our study, congeners considered to be less toxic have the lowest adsorption energy values, which can suggest that they will form complexes with C_60_ more effectively (see Supporting Information File 1 for numerical data). Therefore, the predicted adsorption energy values may suggest that the highest sorption potential will appear for those dioxin congeners which are considered as less toxic. More toxic congeners may have a lower potential to be adsorbed on the fullerene surface while competing with other congeners because of their highest adsorption energies (predicted and calculated). Those predictions are strongly correlated with lateral halogen substitution and a high dipole moment distribution along the *x* axis. It may be suggested that unmodified fullerenes as sorbents can be selectively effective in adsorbing dioxins or other congeners.

The obtained results do not consider the strength of interactions between the AhR receptor and dioxin structure. It is clear that dioxin adsorption on the fullerene surface is possible. We can also assume that, according to one of the proposed mechanisms for nanoparticle toxicity, carbon structures such as fullerenes – because of their sorption abilities – will have the potential to act as vectors. This would allow more pollutants to enter to the cells of the organism. At this point, we have no further knowledge if dioxins adsorbed on the surface would be able to interact with receptors like AhR. It is also possible that they prefer to stay adsorbed, and in fact, might be deactivated as toxic agents.

### Confrontation with WHO TEF values

The obtained results which suggest that most of the PXDD congeners have higher or lower potential to adsorb on the C_60_ surface have created the need to verify the sorption potential according to structural dissimilarities. The comparison of predicted Δ*E*_ads_ values with toxic equivalency factors (TEFs) recommended by the World Health Organization (WHO) [42] seems to indicate that more dangerous congeners will have lowest sorption potential (highest predicted energy of the complex). As presented in Table 1, congeners with official WHO TEF values have relatively high predicted adsorption energies compared with the predicted values for congeners like 1,4,6,9-tetrachlorinated congeners (Δ*E*_ads_ from −4 to −22 kcal/mol), or 2,3,7,8-tetrabromo-substituted congeners (Δ*E*_ads_ from −19 to −22 kcal/mol) (see Supporting Information File 1).

**Table 1:** Predicted adsorption energies for dioxin congeners compared with official WHO TEF values.

IUPAC name	Δ*E*_ads_ [kcal/mol], calculated	Δ*E*_ads_ [kcal/mol], predicted	WHO TEF [42]

2,3,7,8-tetrachlorodibenzo-*p*-dioxin	0.958	0.733	1
1,2,3,7,8-pentachlorodibenzo-*p*-dioxin	prediction set	0.599	1
1,2,3,4,7,8-hexachlorodibenzo-*p*-dioxin	prediction set	−0.738	0.1
1,2,3,6,7,8-hexachlorodibenzo-*p*-dioxin	prediction set	−0.749	0.1
1,2,3,7,8,9-hexachlorodibenzo-*p*-dioxin	prediction set	0.095	0.1
1,2,3,4,6,7,8-heptachlorodibenzo-*p*-dioxin	prediction set	−2.132	0.01
1,2,3,4,6,7,8,9-octochlorodibenzo-*p*-dioxin	prediction set	−2.315	0.0003

As shown in Figure 1b and Table 1, and also in Table S2 in Supporting Information File 1, for some of the congeners, we observed slightly positive *E*_ads_ values in calculations and in predictions as well. Please note that all of the calculations were performed at the standard 298.15 K temperature in gas phase. More importantly, the M06-2X functional is one of the best and it is recommended for modeling of weak interactions, but still it is only an approximation of a real molecule geometry and energy. Errors obtained for this functional can fluctuate around 0.3–0.7 kcal/mol, depending on the type of calculated structure and other calculation parameters [43–45]. Furthermore, adding an error range to the obtained results will cause slightly positive *E*_ads_ values, which should be interpreted as congeners with practically no interaction with the fullerene surface.

It should also be noted that adsorption, as in many other reactions and processes, has an energetic barrier to overcome during the process. In this study, only the energies of single molecules of a fullerene and congeners were calculated, and the energies of the PXDD@C_60_ complexes were calculated without obtaining the transition state energy. A small energetic barrier to overcome during the adsorption process and a slightly higher energy of the complex (comparable to the sum of single molecule energies for calculations in a gas phase (at 298.15 K)) may seem doubtful according to Hess’s law (Equation 2). Additionally, it should be highlighted that all of the PXDD@C_60_ materials are considered to be thermodynamically stable systems, and all of the Hessian matrix eigenvalues were found to be positive, so we are confident that these structures correspond to the minima on the DFT ground state potential energy surface.

## Conclusion

In conclusion, it can be stated that sorption interactions between fullerenes and halogenated dibenzo-*p*-dioxin congeners – based on weak dispersion interactions and π−π stacking between – is possible. It is also strongly dependent on the type and amount of halogen substituents. Because the information about experimentally measured dioxin–fullerene sorption tendencies is still very limited [46], further investigation on the sorption mechanisms on carbon nanoparticles surfaces is essential in order to evaluate the risk, further applications and toxicity assessment. Moreover, the presented Nano-QSPR approach seems to be very helpful in this type of study and in predictions of such weak forces such as dispersion interactions.

Taking into account the inaccuracy of the computational calculations, the predicted values of *E*_ads_ (presented in Table S3 of Supporting Information File 1) suggest that all halogenated dibenzo-*p*-dioxin congeners will interact with the C_60_ surface. The analysis of the obtained predictions shows that congeners that have hydrogen atoms located along the *y* axis (in 1,4,6,9 positions) and halogen atoms mainly in lateral (2,3,7,8) positions will have higher values of adsorption energy. The obtained Nano-QSPR model shows the dependency between the value of predicted *E*_ads_ and the dipole moment. As a result, congeners that are more chlorinated than brominated would have a higher dipole moment along the *x* axis of the dioxin structure and higher predicted values of *E*_ads_. The valuable observation is that structures considered as toxic have lateral halogen atoms and will have higher *E*_ads_ values, unless the number of hydrogen atoms are decreased by chlorine or bromine substitution in positions 1,4,6 or 9. Keeping in mind that brominated and mixed dioxin congeners are present in the environment in mostly unknown concentrations, we hope that the presented results will be considered as a strong signal that further experimental and theoretical studies on sorption mechanisms of organic pollutants on carbon nanomaterial surfaces are critical.

## Experimental

### Congener characterization

A set of 1,701 congeners containing all combinatorial possibilities of bromine, chlorine and mixed (Br/Cl) substitutions of dibenzo-*p*-dioxins was generated as a part of the Persistent Organic Pollutants Big Data project by using ConGENER software [47], and described in more detail in our previous study [48]. A set of 26 so-called molecular descriptors was calculated for each congener at the semi-empirical PM6 level. A descriptor, by definition, is an experimentally measured or calculated numerical parameter describing a particular molecule (e.g., dipole moment, number of halogen atoms, melting temperature, molecular mass, total energy). The list of 26 descriptors used in the project and more detailed information about their calculation is available in Supporting Information File 1, Table S1.

### Subset selection

A significant lack of experimental values and time-consuming calculations, which would be required for analysis of all of the 1,701 PXDDs@C_60_ complexes, led us to select a representative subset of congeners and apply Nano-QSPR modeling of adsorption energies based on highly reliable calculations for selected compounds. A representative subset of 32 PXDDs (2% of the whole 1,701 PXDDs set) was selected by using the Kennard–Stone algorithm (KS) [49–50]. The list of selected congeners and details about the algorithm can be found in Supporting Information File 1, Table S2. This part was performed by using a script m.file [50] in MATLAB 2012 software [51].

### Δ*E*_ads_ calculations for the selected subset

**Figure 4:**
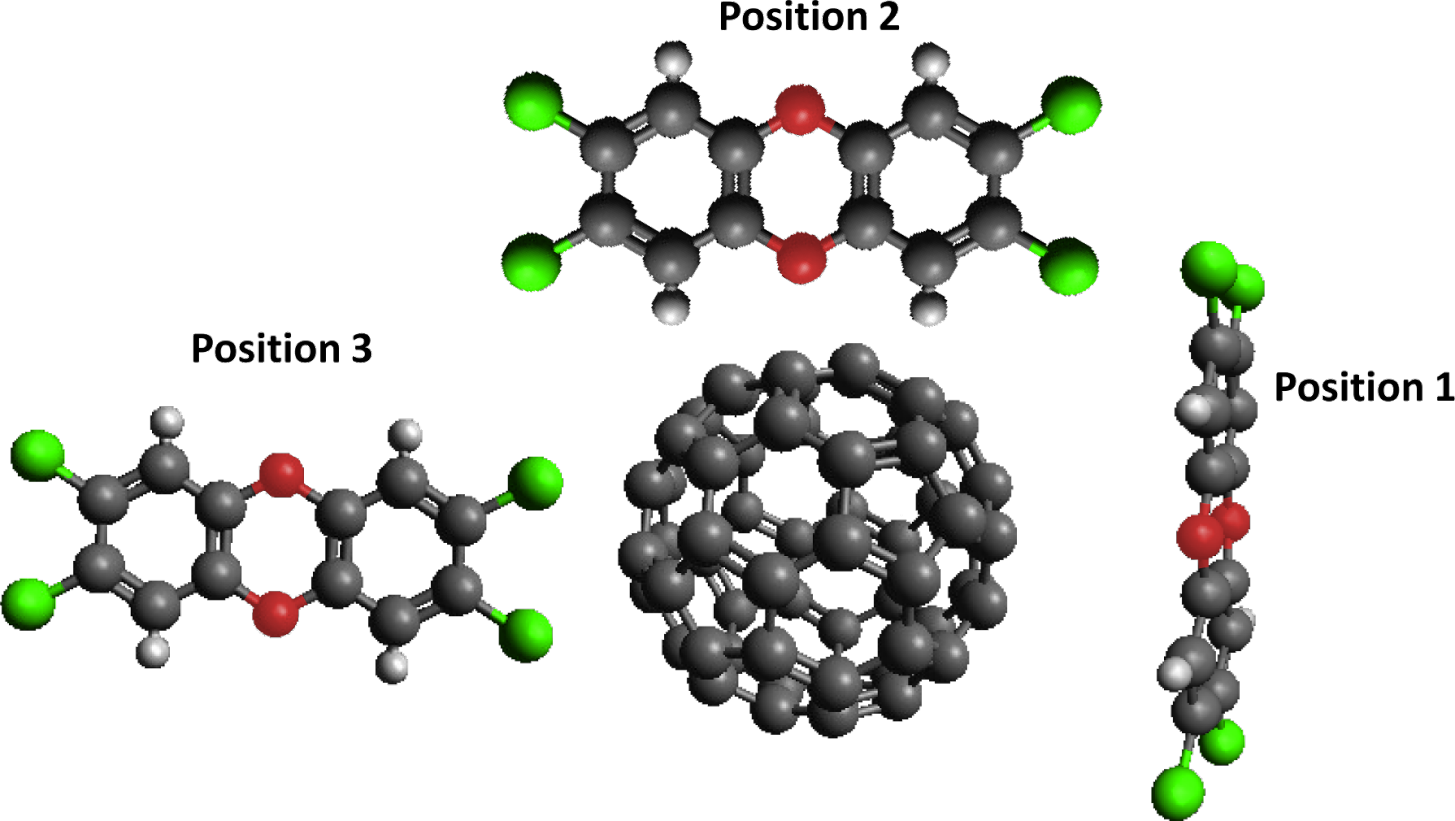
Considered positions of the potential interaction between a fullerene and 2,3,7,8-tetrachlorodibenzo-*p*-dioxin molecule.

In the first stage, three different starting positions (Figure 4) of the 2,3,7,8-tetrachlorodibenzo-*p*-dioxin molecule in the space close to the C_60_ structure were examined to verify if the initial dioxin position has an impact on its final placement as well as on the result of the optimization. The influence of the starting position was checked by molecular mechanics calculations. The influence of the initial distance between the dioxin and fullerene structure was also examined. Calculations on the M06-2X DFT level were performed at this stage for a 2.5–5 Å distance between the PXDD molecule and C_60_ [52]. Supporting Information File 1 provides further details.

A M06-2X DFT functional developed by Truhlar’s group is a hybrid DFT method with partially implemented experimental parameters from different databases. Since it is recognized as one of the best existing methods for showing weak interactions like Van der Waals forces and π–π interactions [53–56], it was applied in the presented study. The 6-31++G(d,p) basis set was used for calculations in this part. Since for each PXDD@C_60_ complex studied in this work all of the Hessian matrix eigenvalues were found to be positive, we are confident that these structures correspond to the minima in the DFT ground state potential energy surface. The adsorption energy (Δ*E*_ads_) was calculated by subtracting the energy of the dioxin–fullerene system from the sum of the separated dioxin and fullerene molecules, according to Hess’s law:

[2]
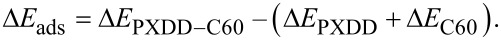


Following the recommendations of the authors of the M06-2X approximations [53], the basis set superposition error (BSSE) was not included. The choice of theoretical methods to obtain the sorption energies for PXDD@C_60_ complexes is reasoned because the values calculated by hybrid DFT methods, especially those calculated with M05-2X and M06-2X, are in good agreement with experimental measurements. What is more, they are appropriate to obtain weak interactions for organic compounds like pesticides or halogenated persistent organic pollutants [55,57–59]. All calculations were performed with the Gaussian 09 program [60].

### Nano-QSPR modeling

The Nano-QSPR method is based on the assumption that the variance in the physicochemical properties of compounds is determined by the variance in their molecular structures. Therefore, it is possible to predict the missing data from the calculated molecular parameters and a suitable mathematical model established for a group of similar chemicals [29]. For details of the QSPR procedure please see Supporting Information File 1.

Holland’s genetic algorithm (GA) [61] was used for the selection of the optimal combination of molecular descriptors and redundancy elimination in the structural data. Partial least squares (PLS) regression was applied as the method of modeling to solve the common problem of co-linearity within a set of descriptors. The PLS method is based on a linear transition of the original variables (descriptors) to a defined number of novel, “latent” variables (latent vectors, LVs) [62]. The use of this method usually results in well-fitted, stable models with high predictive ability [63–64]. The PLS method uses orthogonal latent vectors for regression instead of original descriptors, which is why the coefficients presented in the model equation cannot be individually interpreted. GA-PLS calculations were performed with MATLAB 2012 [51] and PLS Toolbox 7.3 [65] software packages.

To avoid overestimation and to confirm the stability and predictive ability of the developed Nano-QSPR model, a detailed validation procedure was performed, following the recommendations by the Organization for Economic Co-operation and Development (OECD) [29,66]. The details of the procedure are described in Supporting Information File 1.

## Supporting Information

File 1 Adsorption of dibenzo-*p*-dioxins on the surface of C_60_ fullerenes and calculations and QSPR predictions of the influence of halogenation.
Details about the molecular descriptor calculation method, the usage of the Kennard–Stone algorithm, and quantum mechanical calculations can be found in this file. Also, details about the development of the Nano-QSPR model and its statistical characterization are described. Predicted adsorption energies for all chlorinated and/or brominated dibenzo-*p*-dioxin congeners are provided for training and validation sets and for the prediction set.
