# Supplementary material for: Modeling adsorption of brominated, chlorinated and mixed bromo/chloro-dibenzo-p-dioxins on C60 fullerene using Nano-QSPR
Source: Beilstein J Nanotechnol. 2017 Mar 31;8:752–61. doi: 10.3762/bjnano.8.78 (PMC5389196; doi:10.3762/bjnano.8.78)
Supplement: File 1 — Adsorption of dibenzo-p-dioxins on the surface of C60 fullerenes and calculations and QSPR predictions of the influence of halogenation. Details about the molecular descriptor calculation method, the usage of the Kennard–Stone algorithm, and quantum mechanical calculations can be found in this file. Also, details about the development of the Nano-QSPR model and its statistical characterization are described. Predicted adsorption energies for all chlorinated and/or brominated dibenzo-p-dioxin congeners are provided for training and validation sets and for the prediction set. [file Beilstein_J_Nanotechnol-08-752-s001.pdf]

Supporting Information

for

Modeling adsorption of brominated, chlorinated and mixed bromo/chloro-dibenzo-*p*-dioxins on C_{60} fullerene using Nano-QSPR

Piotr Urbaszek[1], Agnieszka Gajewicz[1], Celina Sikorska[2], Maciej Haranczyk[3] and Tomasz Puzyn[1]*

Address: [1]Laboratory of Environmental Chemometrics, Faculty of Chemistry, University of Gdańsk, Wita Stwosza 63, 80-308 Gdańsk, Poland, [2]Laboratory of Molecular Modeling, Faculty of Chemistry, University of Gdańsk, Wita Stwosza 63, 80-308 Gdańsk, Poland and [3]IMDEA Materials Institute, C/Eric Kandel 2, 28906 Getafe, Madrid, Spain

Email: Tomasz Puzyn* - t.puzyn@qsar.eu.org

* Corresponding author

Adsorption of dibenzo-*p*-dioxins on the surface of C_{60} fullerenes and calculations and QSPR predictions of the influence of halogenation

Details on materials and methods

Structural descriptors

Calculations of molecular descriptors can be performed based only on chemical formula or on theoretical chemistry methods. Table S1 presents the list of structural descriptors calculated for all 1701 congeners of brominated, chlorinated and bromo-chlorinated dibenzo-*p*-dioxins. Dioxin database was created as a part of bigger Persistent Organic Pollutants database described in our previous study [1] All calculations were performed with MOPAC 2007 [2] package with using the increased criteria of precision (PRECISE keyword) with PM6 semi-empirical method. Using structural descriptors for Nano-QSPR modeling of sorption potential calculated as an endpoint on DFT level is, as we believe, possible according to the most important conclusions of the latter study [3].

Table S1: List of the structural descriptors.

No.	Symbol	Description	Selected to the QSPR model
**1.**	#H	The number of hydrogen atoms	YES
**2.**	#Cl	Number of chlorine atoms	NO
**3.**	#Br	Number of bromine atoms	NO
**4.**	nAT	Total number of atoms in a molecule	NO
**5.**	HOF	Heat of formation	NO
**6.**	EE	Electronic energy	NO
**7.**	Core	Core repulsion energy	NO
**8.**	TE	Total energy of a molecule	YES
**9.**	HOMO	Energy of the highest occupied molecular orbital	NO
**10.**	LUMO	Energy of the lowest unoccupied molecular orbital	NO
**11.**	HOFc	Heat of formation in water	NO

**12.**	Tec	Total energy in water	NO
**13.**	SAS	Solvent accessible surface	NO
**14.**	MV	Molecular volume	NO
**15.**	MW	Molecular weight	NO
**16.**	D	Dipole moment	NO
**17.**	D_x	X axis dipole moment	YES
**18.**	D_y	Y axis dipole moment	YES
**19.**	D_z	Z axis dipole moment	NO
**20.**	q_min	The lowest negative Mulliken partial charge (q_min)	NO
**21.**	q_max	The highest negative Mulliken partial charge (q_max)	NO
**22.**	Ahof	Polarizability derived from the heat of formation (Ahof)	NO
**23.**	Ad	Polarizability derived from the dipole moment (Ad)	NO
**24.**	EN	Mulliken electronegativity	NO
**25.**	Hard	Parr and Pople's absolute hardness	NO
**26.**	Shift	Schurmann MO shift alpha	NO

Selection of representative subset of dioxins for energy calculations

A subset of 32 representative congeners was selected by using Kennard-Stone algorithm [4]. As a result we got a subset containing the central object in the data, then furthest object from it, than furthest from selected two and so one. So for energy calculations the outliers and farthest objects situated on the surface of the multidimensional cloud formed by all of the objects (compounds) and objects representatively located inside the cloud were chosen.

The starting position influence and starting distance verification

Influence of a starting positions of molecules was verified by Molecular Mechanics calculations. Dioxin and fullerene molecules were built in Avogadro software [5]. A Ghemical Force Field was set up with 500 steps and the Steepest Descent algorithm with convergence on 10-7 level. A Ghemical Force Field is a variant of the Tripos-5.2 Force Field. It is implemented to the Avogadro software, and it was successfully used for carbon nanomaterials like graphene and other chemicals calculations in other studies before [6,7].

Calculations for the 2,3,7,8-TCDD@C60 complexes were performed with initial distances between one of the oxygen atoms from the central dioxin ring and the nearest carbon atom in fullerene. All calculations were performed for position 1 described in manuscript and starting distances: 2.5Å, 3Å, 3.5Å, 4Å, 4.5Å and 5Å respectively. For this part, methods based on Density Functional Theory (so-called DFT methods) were implemented

Quantum mechanical calculations

After a literature research, M06-2X DFT method was chosen. It is known to be suitable for nanoparticles calculations, and what is also relevant, weak Van der Waals and stacking interactions between molecules. Truhlar's hybrid DFT functionals like M05-2X and newer M06-2X, were commonly used in weak interactions modeling, and gave reliable calculation results without the Basis Set Superposition Error (BSSE) corrections, which was proved in previously published studies [8-11].

ONIOM method used in this study allows to model large molecules by defining two or three layers within the structure that are treated at different levels of accuracy. Calibration studies have demonstrated that the resulting predictions are essentially equivalent to those that produced by the high accuracy method alone on the entire molecule. Two layers in the fullerene structure were defined to reduce the computation time in this study. 6-31++g(d,p) basis set was set for high computation layer and 3-21g basis set was used for the lower one (fullerene carbon atoms located on the opposite side from the surface on which the π-π interactions were expected).

Details on the QSPR modeling

QSPR details

The usual QSPR procedure involves (i) collection of experimental data/theoretical calculation of data; (ii) calculation of molecular descriptors for all the studied compounds (this part was fulfilled in earlier studies) [12]; (iii) splitting of the compounds for which experimental/calculated data exist into two sets, a training set (TS) and a validation set (VS); (iv) calibration of the model with the training set; (v) external validation with the validation set; and (vi) if the model passes the validation criteria, prediction of values for new compounds

OECD principles

The OECD principles for (Q)SAR (Quantitative Structure Activity Relationship) methods assume that correctly developed and validated model should have: (i) a well defined predicted endpoint, (ii) an unambiguous algorithm, (iii)a defined domain of applicability, (iv) appropriate measures of goodness-of-fit, robustness and predictivity and (v) a mechanistic interpretation, if possible[13]

Splitting data for training and validation set

After the sorption energy calculations, endpoint value, as an extra column was added to the descriptors matrix. Data was sorted by increasing values of calculated sorption energy for each dioxin structure, and split on training (TS) and validation (VS) sets in a way to be ensured that points from a VS are evenly distributed within the range of the calculated energies in the training set. In sorted data set we utilized the following pattern of splitting: TS-TS-VS-TS-TS-TS-VS-TS-TS-TS-VS-(…)-TS-TS-TS-VS-TS, called 1:X[14] , (where X=4). It was fitted to data to ensure that the first and the last compound in the data set belongs to TS to avoid errors in external validation. Compounds with assignment to TS and VS can be found in Table S2.

Preprocessing of data, internal validation, statistical measures of goodness-of-fit and robustness of the QSPR model

The data set was auto-scaled, so the average value was subtracted from the descriptors, and the resultant values divided by the standard deviation to ensure the same scale and range of all variables.

As goodness-of-fit parameters, the determination coefficient R^2 (1) and the Root mean Square Error of Calibration ($RMSE_C$) (2) were used.

$$R^2 = 1 - \frac{\sum_{i=1}^{n}\left(y_i^{obs} - y_i^{pred}\right)^2}{\sum_{i=1}^{n}\left(y_i^{obs} - \bar{y}^{obs}\right)^2} \qquad (1)$$

$$RMSE_C = \sqrt{\frac{\sum_{n=1}^{n}\left(y_i^{obs} - y_i^{pred}\right)^2}{n}} \qquad (2)$$

where: y_i^{obs} – the experimental (calculated) value of the property for the *i*-th compound; y_i^{pred} – the predicted value for the *i*-th compound; $\tilde{y}^{obs}$ – the mean experimental value of the property in the training set; n – the number of compounds in the training set.

Internal validation (cross-validation leve-one-out technique, CV LOO) was applied to reduce the probability of model's overfitting to the training data, and to measure robustness of the model. According to CV LOO algorithm each dioxin congener from training set was removed, one at a time. Thus, 24 reduced models were calculated; each of it was developed with the remaining 23 congeners in TS and used to predict the sorption energy of the congener temporarily removed. The cross-validated correlation coefficient Q^2_{CV} (3) and cross-validated Root Mean Square Error of Cross-Validation $RMSE_{CV}$ (4) were calculated.

$$Q^2_{CV} = 1 - \frac{\sum_{i=1}^{n}\left(y_i^{obs} - y_i^{predcv}\right)^2}{\sum_{i=1}^{n}\left(y_i^{obs} - \bar{y}^{obs}\right)^2} \tag{3}$$

$$RMSE_{CV} = \sqrt{\frac{\sum_{n=1}^{n}\left(y_i^{obs} - y_i^{predcv}\right)^2}{n}} \tag{4}$$

where y_i^{predcv} – is the predicted value for the temporary excluded (cross-validated) *i*-th compound.

External validation and measures of predictive ability

By applying of the model to the validation set (not previously used to develop the model), we confirmed the models' prediction ability. Predictive ability of the model is described by the externally validated determination coefficient Q^2_{Ext} (5) and the Root Mean Square Error of Prediction $RMSE_P$ (6)

$$Q^2_{Ext} = 1 - \frac{\sum_{j=1}^{k}\left(y_j^{obs} - y_j^{pred}\right)^2}{\sum_{j=1}^{k}\left(y_j^{obs} - \bar{y}^{obs}\right)^2} \tag{5}$$

$$RMSE_P = \sqrt{\frac{\sum_{j=1}^{k}\left(y_j^{obs} - y_j^{pred}\right)^2}{k}} \tag{6}$$

where: y_j^{obs} – the experimental (observed) value of the property for the *j*-th compound; y_j^{pred} – the predicted value for *j*-th compound; $\tilde{y}^{obs}$ – the mean experimental value of the property in the validation set; k – the number of compounds in the validation set.

QSPR model equation and values calculated by using equations 1-6 are presented below:

$$\Delta E_{ads}\ [\text{kcal/mol}] = 7.37\ \mathbf{\#H}\ -6.76\ \mathbf{TE}\ -0.11\ \mathbf{D\_x}\ -\ 0.07\ \mathbf{D\_y}$$

$$R^2 = 0.998 \qquad Q^2_{CV} = 0.987 \qquad Q^2_{Ext} = 0.956 \qquad (7)$$

$$RMSE_C = 1.18 \qquad RMSE_{CV} = 1.468 \qquad RMSE_P = 2.285$$

Applicability domain of the model

The Insubria plot presented on Figure S1 gives the full information about all congeners from training, validation and prediction sets in the space called applicability domain of the QSPR model. It can be noticed that only few from 1701 congeners (from prediction set) have leverage values higher that the critical h*=0.625, what gives the information that those particular predictions are based on extrapolation of a model and are less reliable. The leverage (hi) is defined by (8):

$$h_i = \mathbf{x_i^T}(\mathbf{X^T X})^{-1}\mathbf{x_i} \quad i=(1,\ldots\ldots,n) \qquad (8)$$

where xi is the vector of descriptors calculated for the considered compound and X is the matrix of descriptors calculated for the training set.

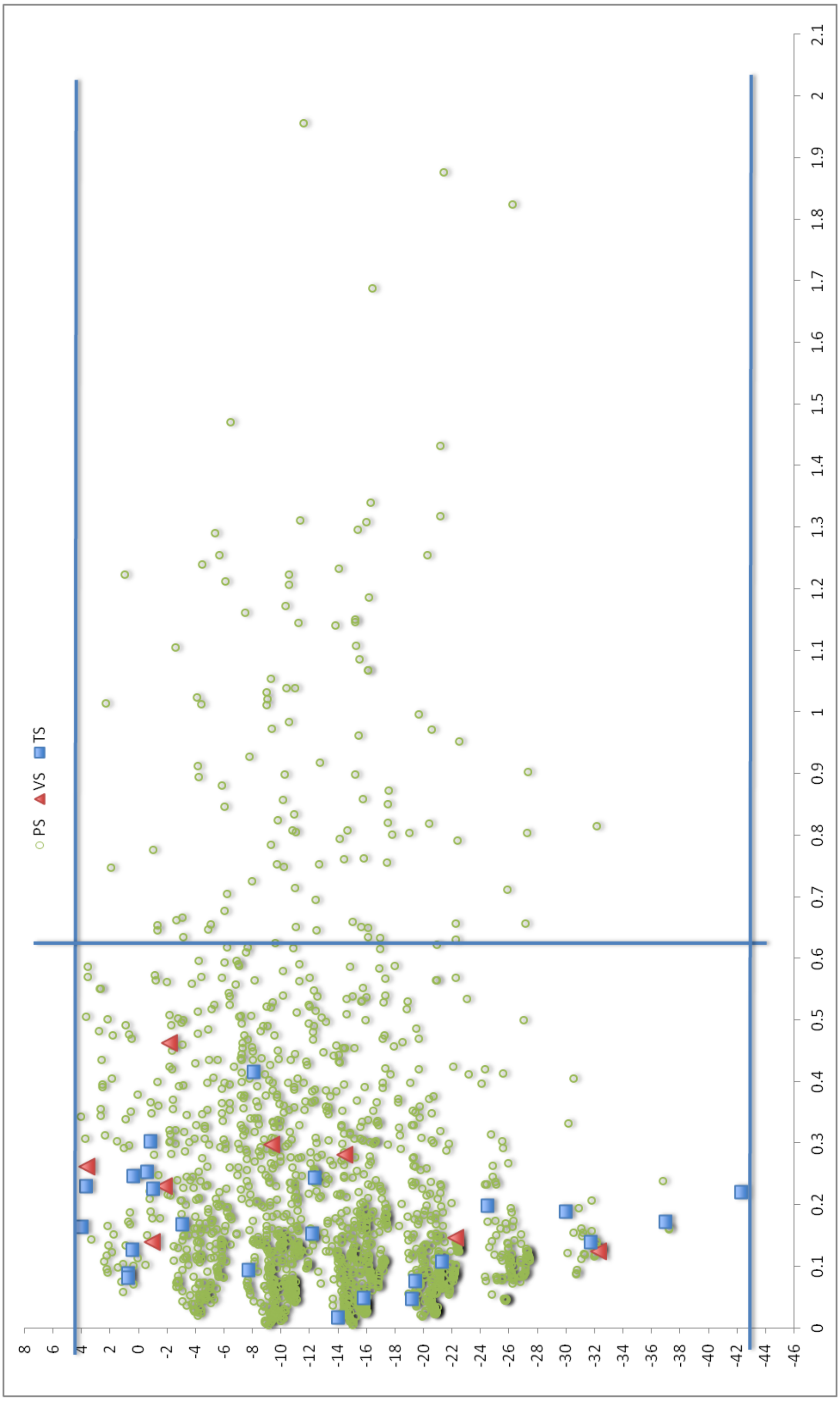

Figure S1: The Insubria Plot, with y_{obs} minimum and maximum values and the h*=0.625 marked by blue lines.

Table S2: List of congeners selected to training and validation sets (TS/VS) with descriptors used in nano-QSPR model (#H - number of H atoms in the molecule, TE - total energy molecule D_x, D_y - dipol moments along X and Y axis). ΔE Calc/Pred Energies calculated/predicted [kcal/mol].

IUPAC name	#H	TE	D_x	D_y	ΔE Calc.	ΔE Pred.	Training/ Validation
1,2,3,4,6,7,8,9-octobromodibenzo-*p*-dioxin	0	-3874.06	0.00	0.00	-41.69	-42.27	TS
1,2,3,4,6,7,8-heptabromodibenzo-*p*-dioxin	1	-3659.79	0.50	-0.02	-35.29	-36.95	TS
1,2,3,7,8,9-hexabromodibenzo-*p*-dioxin	2	-3445.52	-1.13	0.06	-31.47	-29.98	TS
1,2,3,4,6,9-hexabromo-7-chlorodibenzo-*p*-dioxin	1	-3686.27	-0.04	0.01	-31.27	-31.74	TS
1,2,4,7,8-pentabromodibenzo-*p*-dioxin	3	-3231.25	-0.02	-0.02	-24.35	-24.47	TS
1,4,6,9-tetrabromodibenzo-*p*-dioxin	4	-3016.81	0.00	0.01	-22.57	-19.41	TS
1,3,7,9-tetrabromo-4,6-dichlorodibenzo-*p*-dioxin	2	-3498.48	0.82	-0.04	-22.49	-21.29	TS
2,3,7,8-tetrabromodibenzo-*p*-dioxin	4	-3016.97	0.00	0.00	-17.74	-19.17	TS
1,3,8-tribromo-2,6,7-trichlorodibenzo-*p*-dioxin	2	-3524.89	0.10	-0.01	-15.46	-15.81	TS
1,3,9-tribromo-7-chlorodibenzo-*p*-dioxin	4	-3043.52	-0.45	0.03	-14.67	-13.96	TS
1,7-dibromo-2,3,4,6,8,9-hexachlorodibenzo-*p*-dioxin	0	-4033.02	0.03	0.00	-13.64	-12.35	TS
2,8-dibromo-1,3,4,6,7-pentachlorodibenzo-*p*-dioxin	1	-3792.24	0.66	-0.02	-12.57	-12.18	TS
2,8-dibromo-1,3,9-trichlorodibenzo-*p*-dioxin	3	-3310.61	-2.44	0.11	-6.81	-8.07	TS
2,7-dibromodibenzo-*p*-dioxin	6	-2588.22	0.00	0.00	-6.12	-7.70	TS
1-bromo-4,8-dichlorodibenzo-*p*-dioxin	5	-2855.65	-0.14	-0.01	-3.00	-3.04	TS
2-bromodibenzo-*p*-dioxin	7	-2373.74	-1.55	0.08	-1.38	-0.99	TS
1,2,3,4,7-pentachlorodibenzo-*p*-dioxin	3	-3363.81	0.01	0.03	-1.27	-0.83	TS
1,2,3,4,6,9-hexachlorodibenzo-*p*-dioxin	2	-3604.65	-1.06	0.07	-0.48	-0.58	TS
1,4,6,9-tetrachlorodibenzo-*p*-dioxin	4	-3123.04	0.00	0.00	0.29	0.75	TS
2,3,7,8-tetrachlorodibenzo-*p*-dioxin	4	-3123.10	0.00	0.00	0.96	0.73	TS
1,4,6-trichlorodibenzo-*p*-dioxin	5	-2882.11	0.79	-0.02	1.27	0.40	TS
1,2,4,6,9-pentachlorodibenzo-*p*-dioxin	3	-3363.91	-1.03	0.06	2.20	0.42	TS
1-chlorodibenzo-*p*-dioxin	7	-2400.22	-1.31	0.06	2.33	3.98	TS
dibenzo-*p*-dioxin	8	-2159.25	0.00	0.00	4.40	3.72	TS
1,2,3,4,6,8-hexabromo-7,9-dichlorodibenzo-*p*-dioxin	0	-3926.92	-0.07	0.01	-33.19	-32.25	VS
1,2,3,6-tetrabromo-4,7,8,9-tetrachlorodibenzo-*p*-dioxin	0	-3980.04	-0.01	0.00	-22.57	-22.25	VS
1,2,4-tribromo-7,8-dichlorodibenzo-*p*-dioxin	3	-3284.32	-0.01	-0.02	-15.68	-14.44	VS
1,3-dibromo-2,4,8,9-tetrachlorodibenzo-*p*-dioxin	2	-3551.37	-1.75	0.08	-13.58	-9.35	VS
2-bromo-1,8,9-trichlorodibenzo-*p*-dioxin	4	-3096.36	-2.63	0.12	-5.66	-2.15	VS
1,2,3,4,6,8-hexachlorodibenzo-*p*-dioxin	2	-	-0.21	0.02	-0.62	-0.94	VS

		3604.73					
1,2,3,4,6,7,9-heptachlorodibenzo-*p*-dioxin	1	-3845.51	-0.04	0.01	1.19	-1.79	VS
1,9-dichlorodibenzo-*p*-dioxin	6	-2641.17	-2.08	0.10	3.45	3.62	VS

Table S3: Sorption energy values (ΔE_{Pred} [kcal/mol]) predicted for congeners in prediction set. #H – the number of hydrogen atoms in the structure, TE –Total Energy of particular dioxin structure, D_x and D_y – dipole moments along X and Y axis respectively

IUPAC name	#H	TE	D_x	D_y	ΔE Pred
2-chlorodibenzo-*p*-dioxin	7	-2400.308	-1.581	0.089	3.750
1-bromodibenzo-*p*-dioxin	7	-2373.660	-1.281	0.071	-1.373
1,2-dichlorodibenzo-*p*-dioxin	6	-2641.117	-2.380	0.108	4.037
1,3-dichlorodibenzo-*p*-dioxin	6	-2641.250	-1.379	0.088	2.647
1,4-dichlorodibenzo-*p*-dioxin	6	-2641.164	-0.400	0.034	2.142
1,6-dichlorodibenzo-*p*-dioxin	6	-2641.182	0.000	0.003	2.177
1,7-dichlorodibenzo-*p*-dioxin	6	-2641.267	0.279	-0.038	2.719
1,8-dichlorodibenzo-*p*-dioxin	6	-2641.266	-1.053	0.023	3.708
2,3-dichlorodibenzo-*p*-dioxin	6	-2641.193	-1.479	0.096	2.622
2,7-dichlorodibenzo-*p*-dioxin	6	-2641.355	0.000	0.001	2.261
2,8-dichlorodibenzo-*p*-dioxin	6	-2641.353	-1.334	0.058	3.358
1-bromo-2-chlorodibenzo-*p*-dioxin	6	-2614.529	-2.322	0.100	-0.867
1-bromo-3-chlorodibenzo-*p*-dioxin	6	-2614.685	-1.330	0.096	-2.650
1-bromo-4-chlorodibenzo-*p*-dioxin	6	-2614.607	-0.365	0.036	-2.972
1-bromo-6-chlorodibenzo-*p*-dioxin	6	-2614.622	0.038	0.014	-3.176
1-bromo-7-chlorodibenzo-*p*-dioxin	6	-2614.708	0.310	-0.038	-2.335
1-bromo-8-chlorodibenzo-*p*-dioxin	6	-2614.706	-1.024	0.025	-1.394
1-bromo-9-chlorodibenzo-*p*-dioxin	6	-2614.611	-2.045	0.087	-1.049
2-bromo-1-chlorodibenzo-*p*-dioxin	6	-2614.530	-2.337	0.099	-0.812
2-bromo-3-chlorodibenzo-*p*-dioxin	6	-2614.602	-1.446	0.087	-2.208
2-bromo-4-chlorodibenzo-*p*-dioxin	6	-2614.680	-0.635	0.061	-3.086
2-bromo-6-chlorodibenzo-*p*-dioxin	6	-2614.701	-0.251	0.018	-2.707
2-bromo-7-chlorodibenzo-*p*-dioxin	6	-2614.787	0.030	0.000	-2.766
2-bromo-8-chlorodibenzo-*p*-dioxin	6	-2614.787	-1.303	0.050	-1.489
2-bromo-9-chlorodibenzo-*p*-dioxin	6	-2614.699	-2.363	0.121	-1.301
1,2-dibromodibenzo-*p*-dioxin	6	-2588.054	-2.321	0.099	-5.821
1,3-dibromodibenzo-*p*-dioxin	6	-2588.119	-1.339	0.092	-7.525
1,4-dibromodibenzo-*p*-dioxin	6	-2588.046	-0.439	0.037	-7.849
1,7-dibromodibenzo-*p*-dioxin	6	-2588.142	0.282	-0.039	-7.251
1,8-dibromodibenzo-*p*-dioxin	6	-2588.140	-1.004	0.023	-6.376
1,9-dibromodibenzo-*p*-dioxin	6	-2588.052	-1.964	0.093	-6.356
2,3-dibromodibenzo-*p*-dioxin	6	-2588.127	-1.469	0.088	-7.168
2,8-dibromodibenzo-*p*-dioxin	6	-2588.220	-1.286	0.049	-6.491
1,2,3-trichlorodibenzo-*p*-dioxin	5	-2881.994	-2.223	0.119	2.697
1,2,4-trichlorodibenzo-*p*-dioxin	5	-2882.044	-1.468	0.082	2.208
1,2,6-trichlorodibenzo-*p*-dioxin	5	-2882.074	-1.075	0.065	1.896
1,2,7-trichlorodibenzo-*p*-dioxin	5	-2882.159	-0.791	0.031	2.246
1,2,8-trichlorodibenzo-*p*-dioxin	5	-2882.157	-2.099	0.076	3.604
1,2,9-trichlorodibenzo-*p*-dioxin	5	-2882.064	-3.105	0.150	3.609
1,3,6-trichlorodibenzo-*p*-dioxin	5	-2882.205	-0.110	0.041	0.679

1,3,7-trichlorodibenzo-*p*-dioxin	5	-2882.288	0.177	-0.001	1.231
1,3,8-trichlorodibenzo-*p*-dioxin	5	-2882.289	-1.124	0.050	2.421
1,3,9-trichlorodibenzo-*p*-dioxin	5	-2882.195	-2.124	0.119	2.543
1,4,7-trichlorodibenzo-*p*-dioxin	5	-2882.205	1.117	-0.059	0.905
1,7,8-trichlorodibenzo-*p*-dioxin	5	-2882.146	0.214	-0.047	2.327
2,3,7-trichlorodibenzo-*p*-dioxin	5	-2882.232	0.075	0.012	1.080
1-bromo-2,3-dichlorodibenzo-*p*-dioxin	5	-2855.401	-2.149	0.116	-2.369
1-bromo-2,4-dichlorodibenzo-*p*-dioxin	5	-2855.457	-1.412	0.083	-2.925
1-bromo-2,6-dichlorodibenzo-*p*-dioxin	5	-2855.486	-1.006	0.061	-3.133
1-bromo-2,7-dichlorodibenzo-*p*-dioxin	5	-2855.572	-0.726	0.028	-2.801
1-bromo-2,8-dichlorodibenzo-*p*-dioxin	5	-2855.568	-2.038	0.067	-1.279
1-bromo-2,9-dichlorodibenzo-*p*-dioxin	5	-2855.476	-3.041	0.135	-1.125
1-bromo-3,4-dichlorodibenzo-*p*-dioxin	5	-2855.483	-0.612	0.082	-4.440
1-bromo-3,6-dichlorodibenzo-*p*-dioxin	5	-2855.640	-0.053	0.040	-4.400
1-bromo-3,7-dichlorodibenzo-*p*-dioxin	5	-2855.724	0.230	0.005	-4.022
1-bromo-3,8-dichlorodibenzo-*p*-dioxin	5	-2855.725	-1.072	0.049	-2.649
1-bromo-3,9-dichlorodibenzo-*p*-dioxin	5	-2855.631	-2.071	0.108	-2.269
1-bromo-4,6-dichlorodibenzo-*p*-dioxin	5	-2855.555	0.837	-0.001	-5.071
1-bromo-4,7-dichlorodibenzo-*p*-dioxin	5	-2855.648	1.156	-0.057	-4.216
1-bromo-4,9-dichlorodibenzo-*p*-dioxin	5	-2855.555	-1.133	0.044	-2.433
1-bromo-6,7-dichlorodibenzo-*p*-dioxin	5	-2855.515	1.114	-0.073	-3.744
1-bromo-6,8-dichlorodibenzo-*p*-dioxin	5	-2855.645	0.152	-0.015	-3.367
1-bromo-6,9-dichlorodibenzo-*p*-dioxin	5	-2855.553	-0.747	0.051	-3.361
1-bromo-7,8-dichlorodibenzo-*p*-dioxin	5	-2855.587	0.245	-0.053	-2.571
1-bromo-7,9-dichlorodibenzo-*p*-dioxin	5	-2855.636	-0.474	0.005	-2.679
1-bromo-8,9-dichlorodibenzo-*p*-dioxin	5	-2855.504	-1.637	0.066	-2.040
2-bromo-1,3-dichlorodibenzo-*p*-dioxin	5	-2855.370	-2.170	0.112	-2.230
2-bromo-1,4-dichlorodibenzo-*p*-dioxin	5	-2855.452	-1.416	0.076	-2.737
2-bromo-1,6-dichlorodibenzo-*p*-dioxin	5	-2855.487	-1.031	0.060	-3.059
2-bromo-1,7-dichlorodibenzo-*p*-dioxin	5	-2855.570	-0.743	0.030	-2.820
2-bromo-1,8-dichlorodibenzo-*p*-dioxin	5	-2855.569	-2.051	0.068	-1.280
2-bromo-1,9-dichlorodibenzo-*p*-dioxin	5	-2855.476	-3.054	0.138	-1.177
2-bromo-3,4-dichlorodibenzo-*p*-dioxin	5	-2855.398	-0.718	0.079	-4.173
2-bromo-3,6-dichlorodibenzo-*p*-dioxin	5	-2855.555	-0.180	0.029	-3.885
2-bromo-3,7-dichlorodibenzo-*p*-dioxin	5	-2855.639	0.107	0.015	-4.060
2-bromo-3,8-dichlorodibenzo-*p*-dioxin	5	-2855.640	-1.202	0.058	-2.647
2-bromo-3,9-dichlorodibenzo-*p*-dioxin	5	-2855.554	-2.238	0.115	-2.142
2-bromo-4,6-dichlorodibenzo-*p*-dioxin	5	-2855.625	0.555	0.013	-4.876
2-bromo-4,7-dichlorodibenzo-*p*-dioxin	5	-2855.718	0.883	-0.013	-4.818
2-bromo-4,8-dichlorodibenzo-*p*-dioxin	5	-2855.718	-0.414	0.015	-3.039
2-bromo-4,9-dichlorodibenzo-*p*-dioxin	5	-2855.634	-1.448	0.068	-2.433
2-bromo-6,7-dichlorodibenzo-*p*-dioxin	5	-2855.592	0.821	-0.046	-3.865
2-bromo-6,8-dichlorodibenzo-*p*-dioxin	5	-2855.722	-0.143	0.008	-3.380
2-bromo-6,9-dichlorodibenzo-*p*-dioxin	5	-2855.638	-1.080	0.068	-3.143

2-bromo-7,8-dichlorodibenzo-*p*-dioxin	5	-2855.664	-0.040	-0.009	-3.148
2-bromo-7,9-dichlorodibenzo-*p*-dioxin	5	-2855.721	-0.799	0.044	-3.047
2-bromo-8,9-dichlorodibenzo-*p*-dioxin	5	-2855.590	-1.956	0.097	-2.213
1,2-dibromo-3-chlorodibenzo-*p*-dioxin	5	-2828.891	-2.151	0.107	-7.116
1,2-dibromo-4-chlorodibenzo-*p*-dioxin	5	-2828.979	-1.400	0.076	-7.746
1,2-dibromo-6-chlorodibenzo-*p*-dioxin	5	-2829.011	-1.005	0.057	-8.009
1,2-dibromo-7-chlorodibenzo-*p*-dioxin	5	-2829.095	-0.722	0.028	-7.787
1,2-dibromo-8-chlorodibenzo-*p*-dioxin	5	-2829.093	-2.029	0.063	-6.171
1,2-dibromo-9-chlorodibenzo-*p*-dioxin	5	-2828.999	-3.030	0.131	-6.021
1,3-dibromo-2-chlorodibenzo-*p*-dioxin	5	-2828.795	-2.163	0.127	-7.630
1,3-dibromo-4-chlorodibenzo-*p*-dioxin	5	-2828.894	-0.615	0.076	-9.278
1,3-dibromo-6-chlorodibenzo-*p*-dioxin	5	-2829.072	-0.067	0.037	-9.291
1,3-dibromo-7-chlorodibenzo-*p*-dioxin	5	-2829.157	0.219	0.005	-8.997
1,3-dibromo-8-chlorodibenzo-*p*-dioxin	5	-2829.157	-1.078	0.047	-7.581
1,3-dibromo-9-chlorodibenzo-*p*-dioxin	5	-2829.064	-2.076	0.109	-7.280
1,4-dibromo-2-chlorodibenzo-*p*-dioxin	5	-2828.892	-1.499	0.083	-7.752
1,4-dibromo-6-chlorodibenzo-*p*-dioxin	5	-2828.994	0.755	0.007	-10.115
1,4-dibromo-7-chlorodibenzo-*p*-dioxin	5	-2829.087	1.085	-0.052	-9.203
1,6-dibromo-2-chlorodibenzo-*p*-dioxin	5	-2828.928	-1.044	0.079	-8.521
1,6-dibromo-3-chlorodibenzo-*p*-dioxin	5	-2829.081	-0.093	0.035	-9.187
1,6-dibromo-4-chlorodibenzo-*p*-dioxin	5	-2828.996	0.793	-0.011	-9.720
1,7-dibromo-2-chlorodibenzo-*p*-dioxin	5	-2829.004	-0.756	0.031	-7.817
1,7-dibromo-3-chlorodibenzo-*p*-dioxin	5	-2829.158	0.197	-0.001	-8.798
1,7-dibromo-4-chlorodibenzo-*p*-dioxin	5	-2829.081	1.121	-0.058	-9.118
1,7-dibromo-6-chlorodibenzo-*p*-dioxin	5	-2828.927	1.071	-0.071	-8.713
1,7-dibromo-8-chlorodibenzo-*p*-dioxin	5	-2828.995	0.214	-0.055	-7.459
1,7-dibromo-9-chlorodibenzo-*p*-dioxin	5	-2829.066	-0.516	0.005	-7.594
1,8-dibromo-2-chlorodibenzo-*p*-dioxin	5	-2829.002	-2.017	0.069	-6.367
1,8-dibromo-3-chlorodibenzo-*p*-dioxin	5	-2829.157	-1.057	0.047	-7.622
1,8-dibromo-4-chlorodibenzo-*p*-dioxin	5	-2829.081	-0.124	-0.009	-7.985
1,8-dibromo-6-chlorodibenzo-*p*-dioxin	5	-2829.074	0.171	-0.019	-8.296
1,8-dibromo-7-chlorodibenzo-*p*-dioxin	5	-2828.994	0.280	-0.038	-8.028
1,8-dibromo-9-chlorodibenzo-*p*-dioxin	5	-2828.916	-1.619	0.061	-6.945
1,9-dibromo-2-chlorodibenzo-*p*-dioxin	5	-2828.917	-2.964	0.140	-6.397
1,9-dibromo-3-chlorodibenzo-*p*-dioxin	5	-2829.072	-1.995	0.106	-7.357
1,9-dibromo-4-chlorodibenzo-*p*-dioxin	5	-2828.996	-1.056	0.047	-7.654
2,3-dibromo-1-chlorodibenzo-*p*-dioxin	5	-2828.893	-2.194	0.112	-7.163
2,3-dibromo-6-chlorodibenzo-*p*-dioxin	5	-2829.079	-0.209	0.033	-8.911
2,3-dibromo-7-chlorodibenzo-*p*-dioxin	5	-2829.164	0.083	0.014	-8.966
2,7-dibromo-1-chlorodibenzo-*p*-dioxin	5	-2829.004	-0.775	0.032	-7.806
2,7-dibromo-3-chlorodibenzo-*p*-dioxin	5	-2829.072	0.072	0.014	-8.962
2,7-dibromo-4-chlorodibenzo-*p*-dioxin	5	-2829.150	0.846	-0.014	-9.716
2,8-dibromo-1-chlorodibenzo-*p*-dioxin	5	-2829.002	-2.031	0.069	-6.340
2,8-dibromo-3-chlorodibenzo-*p*-dioxin	5	-2829.073	-1.188	0.057	-7.644

2,8-dibromo-4-chlorodibenzo-*p*-dioxin	5	-2829.151	-0.402	0.015	-8.057
1,2,3-tribromodibenzo-*p*-dioxin	5	-2802.416	-2.165	0.110	-12.145
1,2,4-tribromodibenzo-*p*-dioxin	5	-2802.416	-1.488	0.079	-12.648
1,2,6-tribromodibenzo-*p*-dioxin	5	-2802.452	-1.046	0.065	-13.132
1,2,7-tribromodibenzo-*p*-dioxin	5	-2802.528	-0.752	0.030	-12.777
1,2,8-tribromodibenzo-*p*-dioxin	5	-2802.526	-2.009	0.065	-11.257
1,2,9-tribromodibenzo-*p*-dioxin	5	-2802.440	-2.952	0.135	-11.269
1,3,6-tribromodibenzo-*p*-dioxin	5	-2802.513	-0.108	0.033	-14.102
1,3,7-tribromodibenzo-*p*-dioxin	5	-2802.590	0.186	0.004	-13.902
1,3,8-tribromodibenzo-*p*-dioxin	5	-2802.589	-1.063	0.046	-12.580
1,3,9-tribromodibenzo-*p*-dioxin	5	-2802.505	-2.001	0.106	-12.341
1,4,6-tribromodibenzo-*p*-dioxin	5	-2802.435	0.722	-0.002	-14.811
1,4,7-tribromodibenzo-*p*-dioxin	5	-2802.520	1.049	-0.053	-14.103
1,7,8-tribromodibenzo-*p*-dioxin	5	-2802.519	0.244	-0.048	-12.678
2,3,7-tribromodibenzo-*p*-dioxin	5	-2802.597	0.047	0.016	-13.944
1,2,3,4-tetrachlorodibenzo-*p*-dioxin	4	-3122.784	-1.497	0.103	0.942
1,2,3,6-tetrachlorodibenzo-*p*-dioxin	4	-3122.946	-0.953	0.073	0.700
1,2,3,7-tetrachlorodibenzo-*p*-dioxin	4	-3123.029	-0.659	0.047	0.823
1,2,3,8-tetrachlorodibenzo-*p*-dioxin	4	-3123.028	-1.946	0.084	2.349
1,2,3,9-tetrachlorodibenzo-*p*-dioxin	4	-3122.937	-2.932	0.145	2.653
1,2,4,6-tetrachlorodibenzo-*p*-dioxin	4	-3122.988	-0.275	0.047	0.074
1,2,4,7-tetrachlorodibenzo-*p*-dioxin	4	-3123.080	0.055	0.010	0.414
1,2,4,8-tetrachlorodibenzo-*p*-dioxin	4	-3123.078	-1.217	0.035	2.222
1,2,4,9-tetrachlorodibenzo-*p*-dioxin	4	-3122.988	-2.198	0.094	2.569
1,2,6,7-tetrachlorodibenzo-*p*-dioxin	4	-3122.962	0.000	0.001	0.732
1,2,6,8-tetrachlorodibenzo-*p*-dioxin	4	-3123.090	-0.940	0.044	1.456
1,2,6,9-tetrachlorodibenzo-*p*-dioxin	4	-3123.001	-1.822	0.105	1.559
1,2,7,8-tetrachlorodibenzo-*p*-dioxin	4	-3123.032	-0.836	0.020	1.867
1,2,7,9-tetrachlorodibenzo-*p*-dioxin	4	-3123.083	-1.538	0.077	1.753
1,2,8,9-tetrachlorodibenzo-*p*-dioxin	4	-3122.952	-2.682	0.122	2.770
1,3,6,8-tetrachlorodibenzo-*p*-dioxin	4	-3123.222	0.000	0.002	0.755
1,3,6,9-tetrachlorodibenzo-*p*-dioxin	4	-3123.131	-0.880	0.060	0.932
1,3,7,8-tetrachlorodibenzo-*p*-dioxin	4	-3123.162	0.114	-0.018	1.043
1,3,7,9-tetrachlorodibenzo-*p*-dioxin	4	-3123.211	-0.589	0.032	1.112
1,4,7,8-tetrachlorodibenzo-*p*-dioxin	4	-3123.079	1.033	-0.084	0.965
1-bromo-2,3,4-trichlorodibenzo-*p*-dioxin	4	-3096.192	-1.432	0.103	-4.184
1-bromo-2,3,6-trichlorodibenzo-*p*-dioxin	4	-3096.352	-0.868	0.071	-4.412
1-bromo-2,3,7-trichlorodibenzo-*p*-dioxin	4	-3096.436	-0.577	0.043	-4.232
1-bromo-2,3,8-trichlorodibenzo-*p*-dioxin	4	-3096.435	-1.866	0.076	-2.598
1-bromo-2,3,9-trichlorodibenzo-*p*-dioxin	4	-3096.343	-2.852	0.136	-2.269
1-bromo-2,4,6-trichlorodibenzo-*p*-dioxin	4	-3096.401	-0.209	0.048	-5.079
1-bromo-2,4,7-trichlorodibenzo-*p*-dioxin	4	-3096.494	0.118	0.009	-4.681
1-bromo-2,4,8-trichlorodibenzo-*p*-dioxin	4	-3096.491	-1.158	0.033	-2.839
1-bromo-2,4,9-trichlorodibenzo-*p*-dioxin	4	-3096.402	-2.137	0.088	-2.393

1-bromo-2,6,7-trichlorodibenzo-*p*-dioxin	4	-3096.374	0.073	-0.002	-4.331
1-bromo-2,6,8-trichlorodibenzo-*p*-dioxin	4	-3096.502	-0.868	0.040	-3.579
1-bromo-2,6,9-trichlorodibenzo-*p*-dioxin	4	-3096.413	-1.747	0.094	-3.300
1-bromo-2,7,8-trichlorodibenzo-*p*-dioxin	4	-3096.445	-0.771	0.014	-3.102
1-bromo-2,7,9-trichlorodibenzo-*p*-dioxin	4	-3096.496	-1.469	0.065	-3.068
1-bromo-2,8,9-trichlorodibenzo-*p*-dioxin	4	-3096.363	-2.617	0.107	-1.966
1-bromo-3,4,6-trichlorodibenzo-*p*-dioxin	4	-3096.427	0.569	0.029	-6.084
1-bromo-3,4,7-trichlorodibenzo-*p*-dioxin	4	-3096.517	0.894	-0.004	-5.838
1-bromo-3,4,8-trichlorodibenzo-*p*-dioxin	4	-3096.518	-0.377	0.026	-4.161
1-bromo-3,4,9-trichlorodibenzo-*p*-dioxin	4	-3096.426	-1.360	0.072	-3.474
1-bromo-3,6,7-trichlorodibenzo-*p*-dioxin	4	-3096.526	1.001	-0.039	-5.135
1-bromo-3,6,8-trichlorodibenzo-*p*-dioxin	4	-3096.657	0.062	-0.002	-4.256
1-bromo-3,6,9-trichlorodibenzo-*p*-dioxin	4	-3096.566	-0.815	0.056	-4.085
1-bromo-3,7,8-trichlorodibenzo-*p*-dioxin	4	-3096.598	0.170	-0.020	-4.009
1-bromo-3,7,9-trichlorodibenzo-*p*-dioxin	4	-3096.647	-0.531	0.026	-3.840
1-bromo-3,8,9-trichlorodibenzo-*p*-dioxin	4	-3096.518	-1.666	0.081	-3.099
1-bromo-4,6,7-trichlorodibenzo-*p*-dioxin	4	-3096.444	1.870	-0.088	-5.557
1-bromo-4,6,8-trichlorodibenzo-*p*-dioxin	4	-3096.573	0.927	-0.054	-4.593
1-bromo-4,6,9-trichlorodibenzo-*p*-dioxin	4	-3096.483	0.049	0.003	-4.394
1-bromo-4,7,8-trichlorodibenzo-*p*-dioxin	4	-3096.522	1.074	-0.084	-4.108
1-bromo-4,7,9-trichlorodibenzo-*p*-dioxin	4	-3096.574	0.371	-0.038	-3.934
1-bromo-4,8,9-trichlorodibenzo-*p*-dioxin	4	-3096.443	-0.750	0.016	-3.195
1-bromo-6,7,8-trichlorodibenzo-*p*-dioxin	4	-3096.386	0.994	-0.078	-4.135
1-bromo-6,7,9-trichlorodibenzo-*p*-dioxin	4	-3096.428	0.321	-0.032	-4.021
1-bromo-6,8,9-trichlorodibenzo-*p*-dioxin	4	-3096.428	-0.468	0.021	-3.873
1-bromo-7,8,9-trichlorodibenzo-*p*-dioxin	4	-3096.377	-0.374	0.002	-3.571
2-bromo-1,3,4-trichlorodibenzo-*p*-dioxin	4	-3096.154	-1.443	0.092	-3.884
2-bromo-1,3,6-trichlorodibenzo-*p*-dioxin	4	-3096.321	-0.901	0.066	-4.225
2-bromo-1,3,7-trichlorodibenzo-*p*-dioxin	4	-3096.403	-0.606	0.044	-4.208
2-bromo-1,3,8-trichlorodibenzo-*p*-dioxin	4	-3096.404	-1.891	0.076	-2.556
2-bromo-1,3,9-trichlorodibenzo-*p*-dioxin	4	-3096.311	-2.872	0.132	-2.132
2-bromo-1,4,6-trichlorodibenzo-*p*-dioxin	4	-3096.396	-0.223	0.039	-4.820
2-bromo-1,4,7-trichlorodibenzo-*p*-dioxin	4	-3096.486	0.111	0.008	-4.643
2-bromo-1,4,8-trichlorodibenzo-*p*-dioxin	4	-3096.486	-1.160	0.029	-2.733
2-bromo-1,4,9-trichlorodibenzo-*p*-dioxin	4	-3096.395	-2.138	0.085	-2.314
2-bromo-1,6,7-trichlorodibenzo-*p*-dioxin	4	-3096.373	0.047	-0.003	-4.255
2-bromo-1,6,8-trichlorodibenzo-*p*-dioxin	4	-3096.503	-0.890	0.046	-3.692
2-bromo-1,6,9-trichlorodibenzo-*p*-dioxin	4	-3096.412	-1.770	0.099	-3.385
2-bromo-1,7,8-trichlorodibenzo-*p*-dioxin	4	-3096.444	-0.784	0.019	-3.207
2-bromo-1,7,9-trichlorodibenzo-*p*-dioxin	4	-3096.494	-1.484	0.073	-3.248
2-bromo-3,4,6-trichlorodibenzo-*p*-dioxin	4	-3096.341	0.452	0.027	-5.822
2-bromo-3,4,7-trichlorodibenzo-*p*-dioxin	4	-3096.431	0.781	0.010	-6.000
2-bromo-3,4,8-trichlorodibenzo-*p*-dioxin	4	-3096.432	-0.499	0.033	-4.124
2-bromo-3,4,9-trichlorodibenzo-*p*-dioxin	4	-3096.348	-1.517	0.083	-3.471

2-bromo-3,6,7-trichlorodibenzo-*p*-dioxin	4	-3096.440	0.868	-0.036	-4.972
2-bromo-3,6,8-trichlorodibenzo-*p*-dioxin	4	-3096.571	-0.080	0.012	-4.362
2-bromo-3,6,9-trichlorodibenzo-*p*-dioxin	4	-3096.487	-0.994	0.060	-3.858
2-bromo-3,7,8-trichlorodibenzo-*p*-dioxin	4	-3096.512	0.035	-0.002	-4.231
2-bromo-3,7,9-trichlorodibenzo-*p*-dioxin	4	-3096.568	-0.705	0.041	-3.908
2-bromo-3,8,9-trichlorodibenzo-*p*-dioxin	4	-3096.440	-1.841	0.090	-3.009
2-bromo-4,6,7-trichlorodibenzo-*p*-dioxin	4	-3096.512	1.585	-0.055	-5.850
2-bromo-4,6,8-trichlorodibenzo-*p*-dioxin	4	-3096.641	0.638	-0.024	-4.801
2-bromo-4,6,9-trichlorodibenzo-*p*-dioxin	4	-3096.559	-0.277	0.027	-4.373
2-bromo-4,7,8-trichlorodibenzo-*p*-dioxin	4	-3096.591	0.793	-0.040	-4.694
2-bromo-4,7,9-trichlorodibenzo-*p*-dioxin	4	-3096.649	0.052	-0.003	-4.213
2-bromo-4,8,9-trichlorodibenzo-*p*-dioxin	4	-3096.519	-1.067	0.036	-3.088
2-bromo-6,7,8-trichlorodibenzo-*p*-dioxin	4	-3096.462	0.694	-0.044	-4.424
2-bromo-6,7,9-trichlorodibenzo-*p*-dioxin	4	-3096.511	-0.017	0.003	-4.261
2-bromo-6,8,9-trichlorodibenzo-*p*-dioxin	4	-3096.511	-0.804	0.051	-3.987
2-bromo-7,8,9-trichlorodibenzo-*p*-dioxin	4	-3096.460	-0.701	0.039	-3.884
1,2-dibromo-3,4-dichlorodibenzo-*p*-dioxin	4	-3069.678	-1.429	0.093	-8.915
1,2-dibromo-3,6-dichlorodibenzo-*p*-dioxin	4	-3069.842	-0.871	0.066	-9.262
1,2-dibromo-3,7-dichlorodibenzo-*p*-dioxin	4	-3069.925	-0.580	0.043	-9.211
1,2-dibromo-3,8-dichlorodibenzo-*p*-dioxin	4	-3069.925	-1.863	0.072	-7.485
1,2-dibromo-3,9-dichlorodibenzo-*p*-dioxin	4	-3069.832	-2.845	0.127	-7.033
1,2-dibromo-4,6-dichlorodibenzo-*p*-dioxin	4	-3069.922	-0.197	0.039	-9.848
1,2-dibromo-4,7-dichlorodibenzo-*p*-dioxin	4	-3070.013	0.133	0.012	-9.767
1,2-dibromo-4,8-dichlorodibenzo-*p*-dioxin	4	-3070.012	-1.137	0.029	-7.755
1,2-dibromo-4,9-dichlorodibenzo-*p*-dioxin	4	-3069.921	-2.115	0.080	-7.207
1,2-dibromo-6,7-dichlorodibenzo-*p*-dioxin	4	-3069.898	0.077	-0.002	-9.317
1,2-dibromo-6,8-dichlorodibenzo-*p*-dioxin	4	-3070.026	-0.860	0.038	-8.521
1,2-dibromo-6,9-dichlorodibenzo-*p*-dioxin	4	-3069.936	-1.737	0.093	-8.272
1,2-dibromo-7,8-dichlorodibenzo-*p*-dioxin	4	-3069.968	-0.760	0.014	-8.102
1,2-dibromo-7,9-dichlorodibenzo-*p*-dioxin	4	-3070.018	-1.457	0.067	-8.122
1,2-dibromo-8,9-dichlorodibenzo-*p*-dioxin	4	-3069.887	-2.602	0.102	-6.844
1,3-dibromo-2,4-dichlorodibenzo-*p*-dioxin	4	-3069.552	-1.439	0.107	-9.283
1,3-dibromo-2,6-dichlorodibenzo-*p*-dioxin	4	-3069.745	-0.888	0.085	-9.740
1,3-dibromo-2,7-dichlorodibenzo-*p*-dioxin	4	-3069.830	-0.596	0.049	-9.354
1,3-dibromo-2,8-dichlorodibenzo-*p*-dioxin	4	-3069.828	-1.877	0.076	-7.580
1,3-dibromo-2,9-dichlorodibenzo-*p*-dioxin	4	-3069.738	-2.862	0.135	-7.226
1,3-dibromo-4,6-dichlorodibenzo-*p*-dioxin	4	-3069.837	0.558	0.024	-10.933
1,3-dibromo-4,7-dichlorodibenzo-*p*-dioxin	4	-3069.928	0.887	-0.010	-10.669
1,3-dibromo-4,8-dichlorodibenzo-*p*-dioxin	4	-3069.927	-0.381	0.023	-9.076
1,3-dibromo-4,9-dichlorodibenzo-*p*-dioxin	4	-3069.837	-1.361	0.076	-8.576
1,3-dibromo-6,7-dichlorodibenzo-*p*-dioxin	4	-3069.958	0.986	-0.033	-10.257
1,3-dibromo-6,8-dichlorodibenzo-*p*-dioxin	4	-3070.087	0.053	0.008	-9.495
1,3-dibromo-6,9-dichlorodibenzo-*p*-dioxin	4	-3069.998	-0.825	0.069	-9.399
1,3-dibromo-7,8-dichlorodibenzo-*p*-dioxin	4	-3070.030	0.160	-0.020	-8.985

1,3-dibromo-7,9-dichlorodibenzo-*p*-dioxin	4	-3070.080	-0.540	0.026	-8.818
1,3-dibromo-8,9-dichlorodibenzo-*p*-dioxin	4	-3069.950	-1.670	0.079	-8.035
1,4-dibromo-2,3-dichlorodibenzo-*p*-dioxin	4	-3069.596	-1.518	0.102	-8.993
1,4-dibromo-2,6-dichlorodibenzo-*p*-dioxin	4	-3069.836	-0.303	0.061	-10.231
1,4-dibromo-2,7-dichlorodibenzo-*p*-dioxin	4	-3069.928	0.033	0.016	-9.694
1,4-dibromo-2,8-dichlorodibenzo-*p*-dioxin	4	-3069.926	-1.243	0.032	-7.644
1,4-dibromo-2,9-dichlorodibenzo-*p*-dioxin	4	-3069.836	-2.222	0.087	-7.198
1,4-dibromo-6,7-dichlorodibenzo-*p*-dioxin	4	-3069.882	1.793	-0.082	-10.558
1,4-dibromo-6,8-dichlorodibenzo-*p*-dioxin	4	-3070.012	0.849	-0.052	-9.489
1,4-dibromo-6,9-dichlorodibenzo-*p*-dioxin	4	-3069.922	-0.030	0.007	-9.340
1,4-dibromo-7,8-dichlorodibenzo-*p*-dioxin	4	-3069.961	1.006	-0.084	-8.971
1,6-dibromo-2,3-dichlorodibenzo-*p*-dioxin	4	-3069.793	-0.908	0.084	-9.667
1,6-dibromo-2,4-dichlorodibenzo-*p*-dioxin	4	-3069.843	-0.254	0.055	-10.168
1,6-dibromo-2,7-dichlorodibenzo-*p*-dioxin	4	-3069.788	0.000	0.006	-9.397
1,6-dibromo-2,8-dichlorodibenzo-*p*-dioxin	4	-3069.939	-0.928	0.055	-8.848
1,6-dibromo-2,9-dichlorodibenzo-*p*-dioxin	4	-3069.857	-1.793	0.109	-8.594
1,6-dibromo-3,4-dichlorodibenzo-*p*-dioxin	4	-3069.867	0.524	0.020	-10.758
1,6-dibromo-3,8-dichlorodibenzo-*p*-dioxin	4	-3070.092	0.000	0.006	-9.340
1,6-dibromo-3,9-dichlorodibenzo-*p*-dioxin	4	-3070.009	-0.861	0.071	-9.380
1,6-dibromo-4,9-dichlorodibenzo-*p*-dioxin	4	-3069.927	0.001	0.014	-9.580
1,7-dibromo-2,3-dichlorodibenzo-*p*-dioxin	4	-3069.868	-0.612	0.045	-9.212
1,7-dibromo-2,4-dichlorodibenzo-*p*-dioxin	4	-3069.925	0.081	0.011	-9.657
1,7-dibromo-2,6-dichlorodibenzo-*p*-dioxin	4	-3069.786	0.026	0.001	-9.318
1,7-dibromo-2,8-dichlorodibenzo-*p*-dioxin	4	-3069.852	-0.800	0.018	-8.151
1,7-dibromo-2,9-dichlorodibenzo-*p*-dioxin	4	-3069.924	-1.513	0.071	-8.136
1,7-dibromo-3,4-dichlorodibenzo-*p*-dioxin	4	-3069.950	0.857	-0.002	-10.814
1,7-dibromo-3,6-dichlorodibenzo-*p*-dioxin	4	-3069.939	0.953	-0.038	-10.067
1,7-dibromo-3,8-dichlorodibenzo-*p*-dioxin	4	-3070.006	0.137	-0.020	-8.945
1,7-dibromo-3,9-dichlorodibenzo-*p*-dioxin	4	-3070.077	-0.578	0.030	-8.849
1,7-dibromo-4,6-dichlorodibenzo-*p*-dioxin	4	-3069.855	1.819	-0.088	-10.458
1,7-dibromo-4,8-dichlorodibenzo-*p*-dioxin	4	-3069.929	1.037	-0.085	-9.011
1,7-dibromo-4,9-dichlorodibenzo-*p*-dioxin	4	-3070.002	0.322	-0.038	-8.836
1,7-dibromo-6,8-dichlorodibenzo-*p*-dioxin	4	-3069.761	0.944	-0.081	-8.967
1,7-dibromo-6,9-dichlorodibenzo-*p*-dioxin	4	-3069.836	0.271	-0.031	-8.951
1,7-dibromo-8,9-dichlorodibenzo-*p*-dioxin	4	-3069.780	-0.411	-0.004	-8.344
1,8-dibromo-2,3-dichlorodibenzo-*p*-dioxin	4	-3069.867	-1.849	0.078	-7.679
1,8-dibromo-2,4-dichlorodibenzo-*p*-dioxin	4	-3069.924	-1.142	0.043	-8.125
1,8-dibromo-2,6-dichlorodibenzo-*p*-dioxin	4	-3069.931	-0.847	0.047	-8.798
1,8-dibromo-2,7-dichlorodibenzo-*p*-dioxin	4	-3069.852	-0.735	0.018	-8.276
1,8-dibromo-2,9-dichlorodibenzo-*p*-dioxin	4	-3069.776	-2.598	0.116	-7.236
1,8-dibromo-3,4-dichlorodibenzo-*p*-dioxin	4	-3069.950	-0.366	0.022	-9.075
1,8-dibromo-3,6-dichlorodibenzo-*p*-dioxin	4	-3070.085	0.078	0.004	-9.440
1,8-dibromo-3,7-dichlorodibenzo-*p*-dioxin	4	-3070.004	0.198	-0.016	-9.167
1,8-dibromo-3,9-dichlorodibenzo-*p*-dioxin	4	-3069.929	-1.651	0.077	-8.024

1,8-dibromo-4,6-dichlorodibenzo-*p*-dioxin	4	-3070.002	0.942	-0.046	-9.826
1,8-dibromo-4,7-dichlorodibenzo-*p*-dioxin	4	-3069.930	1.101	-0.071	-9.498
1,8-dibromo-4,9-dichlorodibenzo-*p*-dioxin	4	-3069.855	-0.737	0.012	-8.116
1,8-dibromo-6,7-dichlorodibenzo-*p*-dioxin	4	-3069.789	1.019	-0.070	-9.392
1,8-dibromo-6,9-dichlorodibenzo-*p*-dioxin	4	-3069.835	-0.450	0.017	-8.804
1,8-dibromo-7,9-dichlorodibenzo-*p*-dioxin	4	-3069.752	-0.345	0.000	-8.581
1,9-dibromo-2,3-dichlorodibenzo-*p*-dioxin	4	-3069.785	-2.780	0.135	-7.376
1,9-dibromo-2,4-dichlorodibenzo-*p*-dioxin	4	-3069.842	-2.062	0.091	-7.610
1,9-dibromo-2,6-dichlorodibenzo-*p*-dioxin	4	-3069.856	-1.684	0.098	-8.519
1,9-dibromo-2,7-dichlorodibenzo-*p*-dioxin	4	-3069.932	-1.378	0.069	-8.343
1,9-dibromo-2,8-dichlorodibenzo-*p*-dioxin	4	-3069.776	-2.526	0.116	-7.375
1,9-dibromo-3,4-dichlorodibenzo-*p*-dioxin	4	-3069.868	-1.284	0.074	-8.667
1,9-dibromo-3,6-dichlorodibenzo-*p*-dioxin	4	-3070.010	-0.752	0.050	-9.045
1,9-dibromo-3,7-dichlorodibenzo-*p*-dioxin	4	-3070.084	-0.440	0.022	-8.906
1,9-dibromo-4,6-dichlorodibenzo-*p*-dioxin	4	-3069.927	0.114	-0.001	-9.409
2,3-dibromo-1,4-dichlorodibenzo-*p*-dioxin	4	-3069.644	-1.464	0.091	-8.802
2,3-dibromo-1,6-dichlorodibenzo-*p*-dioxin	4	-3069.843	-0.930	0.067	-9.174
2,3-dibromo-1,7-dichlorodibenzo-*p*-dioxin	4	-3069.926	-0.632	0.042	-9.085
2,3-dibromo-1,8-dichlorodibenzo-*p*-dioxin	4	-3069.926	-1.911	0.077	-7.522
2,3-dibromo-1,9-dichlorodibenzo-*p*-dioxin	4	-3069.834	-2.893	0.136	-7.174
2,3-dibromo-6,7-dichlorodibenzo-*p*-dioxin	4	-3069.964	0.839	-0.030	-10.050
2,3-dibromo-6,8-dichlorodibenzo-*p*-dioxin	4	-3070.093	-0.103	0.011	-9.270
2,3-dibromo-6,9-dichlorodibenzo-*p*-dioxin	4	-3070.011	-1.017	0.064	-8.896
2,3-dibromo-7,8-dichlorodibenzo-*p*-dioxin	4	-3070.036	0.013	-0.001	-9.193
2,7-dibromo-1,3-dichlorodibenzo-*p*-dioxin	4	-3069.836	-0.641	0.047	-9.214
2,7-dibromo-1,4-dichlorodibenzo-*p*-dioxin	4	-3069.918	0.073	0.008	-9.565
2,7-dibromo-1,6-dichlorodibenzo-*p*-dioxin	4	-3069.786	0.000	0.001	-9.267
2,7-dibromo-1,8-dichlorodibenzo-*p*-dioxin	4	-3069.852	-0.816	0.022	-8.224
2,7-dibromo-1,9-dichlorodibenzo-*p*-dioxin	4	-3069.923	-1.530	0.080	-8.337
2,7-dibromo-3,4-dichlorodibenzo-*p*-dioxin	4	-3069.863	0.742	0.010	-10.920
2,7-dibromo-3,8-dichlorodibenzo-*p*-dioxin	4	-3069.920	0.000	0.001	-9.242
2,7-dibromo-3,9-dichlorodibenzo-*p*-dioxin	4	-3069.998	-0.755	0.048	-8.989
2,7-dibromo-4,9-dichlorodibenzo-*p*-dioxin	4	-3070.078	0.000	0.000	-9.186
2,8-dibromo-1,3-dichlorodibenzo-*p*-dioxin	4	-3069.835	-1.874	0.075	-7.559
2,8-dibromo-1,4-dichlorodibenzo-*p*-dioxin	4	-3069.918	-1.145	0.032	-7.835
2,8-dibromo-1,6-dichlorodibenzo-*p*-dioxin	4	-3069.931	-0.870	0.044	-8.676
2,8-dibromo-1,7-dichlorodibenzo-*p*-dioxin	4	-3069.851	-0.750	0.022	-8.351
2,8-dibromo-1,9-dichlorodibenzo-*p*-dioxin	4	-3069.774	-2.611	0.114	-7.159
2,8-dibromo-3,4-dichlorodibenzo-*p*-dioxin	4	-3069.865	-0.488	0.034	-9.166
2,8-dibromo-3,6-dichlorodibenzo-*p*-dioxin	4	-3069.999	-0.065	0.010	-9.335
2,8-dibromo-3,7-dichlorodibenzo-*p*-dioxin	4	-3069.919	0.061	-0.003	-9.256
2,8-dibromo-4,6-dichlorodibenzo-*p*-dioxin	4	-3070.071	0.652	-0.025	-9.798
1,2,3-tribromo-4-chlorodibenzo-*p*-dioxin	4	-3043.171	-1.439	0.091	-13.829
1,2,3-tribromo-6-chlorodibenzo-*p*-dioxin	4	-3043.366	-0.892	0.068	-14.252

1,2,3-tribromo-7-chlorodibenzo-*p*-dioxin	4	-3043.450	-0.598	0.042	-14.129
1,2,3-tribromo-8-chlorodibenzo-*p*-dioxin	4	-3043.448	-1.875	0.073	-12.467
1,2,3-tribromo-9-chlorodibenzo-*p*-dioxin	4	-3043.357	-2.856	0.127	-11.991
1,2,4-tribromo-3-chlorodibenzo-*p*-dioxin	4	-3043.072	-1.517	0.097	-13.852
1,2,4-tribromo-6-chlorodibenzo-*p*-dioxin	4	-3043.359	-0.292	0.052	-14.997
1,2,4-tribromo-7-chlorodibenzo-*p*-dioxin	4	-3043.450	0.046	0.012	-14.594
1,2,4-tribromo-8-chlorodibenzo-*p*-dioxin	4	-3043.450	-1.225	0.027	-12.528
1,2,4-tribromo-9-chlorodibenzo-*p*-dioxin	4	-3043.359	-2.202	0.078	-11.982
1,2,6-tribromo-3-chlorodibenzo-*p*-dioxin	4	-3043.283	-0.915	0.068	-14.223
1,2,6-tribromo-4-chlorodibenzo-*p*-dioxin	4	-3043.363	-0.243	0.056	-15.194
1,2,6-tribromo-7-chlorodibenzo-*p*-dioxin	4	-3043.311	0.002	0.003	-14.301
1,2,6-tribromo-8-chlorodibenzo-*p*-dioxin	4	-3043.463	-0.921	0.047	-13.632
1,2,6-tribromo-9-chlorodibenzo-*p*-dioxin	4	-3043.380	-1.785	0.103	-13.432
1,2,7-tribromo-3-chlorodibenzo-*p*-dioxin	4	-3043.358	-0.614	0.044	-14.167
1,2,7-tribromo-4-chlorodibenzo-*p*-dioxin	4	-3043.445	0.097	0.008	-14.590
1,2,7-tribromo-6-chlorodibenzo-*p*-dioxin	4	-3043.310	0.030	-0.002	-14.226
1,2,7-tribromo-8-chlorodibenzo-*p*-dioxin	4	-3043.376	-0.789	0.017	-13.124
1,2,7-tribromo-9-chlorodibenzo-*p*-dioxin	4	-3043.447	-1.500	0.071	-13.139
1,2,8-tribromo-3-chlorodibenzo-*p*-dioxin	4	-3043.356	-1.846	0.073	-12.540
1,2,8-tribromo-4-chlorodibenzo-*p*-dioxin	4	-3043.444	-1.122	0.032	-12.858
1,2,8-tribromo-6-chlorodibenzo-*p*-dioxin	4	-3043.455	-0.838	0.041	-13.638
1,2,8-tribromo-7-chlorodibenzo-*p*-dioxin	4	-3043.375	-0.725	0.018	-13.274
1,2,8-tribromo-9-chlorodibenzo-*p*-dioxin	4	-3043.298	-2.584	0.104	-11.930
1,2,9-tribromo-3-chlorodibenzo-*p*-dioxin	4	-3043.274	-2.770	0.129	-12.224
1,2,9-tribromo-4-chlorodibenzo-*p*-dioxin	4	-3043.362	-2.038	0.083	-12.428
1,2,9-tribromo-6-chlorodibenzo-*p*-dioxin	4	-3043.379	-1.672	0.091	-13.339
1,2,9-tribromo-7-chlorodibenzo-*p*-dioxin	4	-3043.454	-1.364	0.071	-13.401
1,2,9-tribromo-8-chlorodibenzo-*p*-dioxin	4	-3043.300	-2.510	0.115	-12.358
1,3,6-tribromo-2-chlorodibenzo-*p*-dioxin	4	-3043.187	-0.930	0.084	-14.627
1,3,6-tribromo-4-chlorodibenzo-*p*-dioxin	4	-3043.278	0.512	0.014	-15.579
1,3,6-tribromo-7-chlorodibenzo-*p*-dioxin	4	-3043.371	0.913	-0.035	-15.063
1,3,6-tribromo-8-chlorodibenzo-*p*-dioxin	4	-3043.523	-0.010	0.008	-14.368
1,3,6-tribromo-9-chlorodibenzo-*p*-dioxin	4	-3043.441	-0.872	0.071	-14.354
1,3,7-tribromo-2-chlorodibenzo-*p*-dioxin	4	-3043.262	-0.630	0.048	-14.258
1,3,7-tribromo-4-chlorodibenzo-*p*-dioxin	4	-3043.360	0.850	-0.006	-15.697
1,3,7-tribromo-6-chlorodibenzo-*p*-dioxin	4	-3043.370	0.937	-0.037	-15.058
1,3,7-tribromo-8-chlorodibenzo-*p*-dioxin	4	-3043.437	0.127	-0.019	-13.948
1,3,7-tribromo-9-chlorodibenzo-*p*-dioxin	4	-3043.509	-0.587	0.029	-13.801
1,3,8-tribromo-2-chlorodibenzo-*p*-dioxin	4	-3043.261	-1.859	0.081	-12.741
1,3,8-tribromo-4-chlorodibenzo-*p*-dioxin	4	-3043.360	-0.369	0.020	-14.017
1,3,8-tribromo-6-chlorodibenzo-*p*-dioxin	4	-3043.516	0.068	0.005	-14.442
1,3,8-tribromo-7-chlorodibenzo-*p*-dioxin	4	-3043.437	0.188	-0.014	-14.196
1,3,8-tribromo-9-chlorodibenzo-*p*-dioxin	4	-3043.362	-1.655	0.076	-12.986
1,3,9-tribromo-2-chlorodibenzo-*p*-dioxin	4	-3043.179	-2.790	0.136	-12.385

1,3,9-tribromo-4-chlorodibenzo-*p*-dioxin	4	-3043.278	-1.286	0.070	-13.559
1,3,9-tribromo-6-chlorodibenzo-*p*-dioxin	4	-3043.441	-0.763	0.054	-14.123
1,3,9-tribromo-8-chlorodibenzo-*p*-dioxin	4	-3043.364	-1.579	0.077	-13.158
1,4,6-tribromo-2-chlorodibenzo-*p*-dioxin	4	-3043.278	-0.337	0.065	-15.263
1,4,6-tribromo-3-chlorodibenzo-*p*-dioxin	4	-3043.278	0.445	0.033	-15.943
1,4,6-tribromo-7-chlorodibenzo-*p*-dioxin	4	-3043.296	1.727	-0.086	-15.326
1,4,6-tribromo-8-chlorodibenzo-*p*-dioxin	4	-3043.449	0.796	-0.051	-14.407
1,4,6-tribromo-9-chlorodibenzo-*p*-dioxin	4	-3043.366	-0.068	0.010	-14.338
1,4,7-tribromo-2-chlorodibenzo-*p*-dioxin	4	-3043.360	-0.005	0.016	-14.616
1,4,7-tribromo-3-chlorodibenzo-*p*-dioxin	4	-3043.359	0.777	-0.003	-15.634
1,4,7-tribromo-6-chlorodibenzo-*p*-dioxin	4	-3043.294	1.742	-0.079	-15.537
1,4,7-tribromo-8-chlorodibenzo-*p*-dioxin	4	-3043.368	0.968	-0.087	-13.820
1,4,7-tribromo-9-chlorodibenzo-*p*-dioxin	4	-3043.441	0.252	-0.038	-13.695
1,7,8-tribromo-2-chlorodibenzo-*p*-dioxin	4	-3043.376	-0.771	0.020	-13.237
1,7,8-tribromo-3-chlorodibenzo-*p*-dioxin	4	-3043.529	0.161	-0.019	-13.996
1,7,8-tribromo-4-chlorodibenzo-*p*-dioxin	4	-3043.454	1.060	-0.082	-14.112
1,7,8-tribromo-6-chlorodibenzo-*p*-dioxin	4	-3043.283	0.975	-0.065	-14.421
1,7,8-tribromo-9-chlorodibenzo-*p*-dioxin	4	-3043.275	-0.383	0.008	-13.694
2,3,7-tribromo-1-chlorodibenzo-*p*-dioxin	4	-3043.359	-0.668	0.046	-14.114
2,3,7-tribromo-4-chlorodibenzo-*p*-dioxin	4	-3043.357	0.719	0.007	-15.782
2,3,7-tribromo-6-chlorodibenzo-*p*-dioxin	4	-3043.375	0.786	-0.028	-14.999
2,3,7-tribromo-8-chlorodibenzo-*p*-dioxin	4	-3043.443	-0.022	0.001	-14.178
2,3,7-tribromo-9-chlorodibenzo-*p*-dioxin	4	-3043.522	-0.776	0.049	-13.953
1,2,3,4-tetrabromodibenzo-*p*-dioxin	4	-3016.693	-1.521	0.096	-18.779
1,2,3,6-tetrabromodibenzo-*p*-dioxin	4	-3016.807	-0.935	0.086	-19.630
1,2,3,7-tetrabromodibenzo-*p*-dioxin	4	-3016.882	-0.632	0.048	-19.215
1,2,3,8-tetrabromodibenzo-*p*-dioxin	4	-3016.881	-1.858	0.073	-17.495
1,2,3,9-tetrabromodibenzo-*p*-dioxin	4	-3016.798	-2.782	0.129	-17.180
1,2,4,6-tetrabromodibenzo-*p*-dioxin	4	-3016.801	-0.328	0.065	-20.259
1,2,4,7-tetrabromodibenzo-*p*-dioxin	4	-3016.883	0.009	0.016	-19.622
1,2,4,8-tetrabromodibenzo-*p*-dioxin	4	-3016.882	-1.208	0.030	-17.634
1,2,4,9-tetrabromodibenzo-*p*-dioxin	4	-3016.800	-2.125	0.078	-17.124
1,2,6,7-tetrabromodibenzo-*p*-dioxin	4	-3016.835	0.000	0.001	-19.224
1,2,6,8-tetrabromodibenzo-*p*-dioxin	4	-3016.895	-0.905	0.056	-18.892
1,2,6,9-tetrabromodibenzo-*p*-dioxin	4	-3016.819	-1.716	0.101	-18.508
1,2,7,8-tetrabromodibenzo-*p*-dioxin	4	-3016.899	-0.760	0.020	-18.237
1,2,7,9-tetrabromodibenzo-*p*-dioxin	4	-3016.886	-1.408	0.075	-18.416
1,2,8,9-tetrabromodibenzo-*p*-dioxin	4	-3016.823	-2.495	0.113	-17.314
1,3,6,8-tetrabromodibenzo-*p*-dioxin	4	-3016.955	0.001	0.010	-19.437
1,3,6,9-tetrabromodibenzo-*p*-dioxin	4	-3016.881	-0.806	0.060	-19.190
1,3,7,8-tetrabromodibenzo-*p*-dioxin	4	-3016.961	0.150	-0.020	-18.945
1,3,7,9-tetrabromodibenzo-*p*-dioxin	4	-3016.949	-0.497	0.027	-18.918
1,4,7,8-tetrabromodibenzo-*p*-dioxin	4	-3016.892	0.992	-0.081	-19.001
1,2,3,4,6-pentachlorodibenzo-*p*-dioxin	3	-3363.725	-0.322	0.063	-1.028

1,2,3,6,7-pentachlorodibenzo-*p*-dioxin	3	-3363.828	0.104	0.010	-0.456
1,2,3,6,8-pentachlorodibenzo-*p*-dioxin	3	-3363.958	-0.820	0.047	0.393
1,2,3,6,9-pentachlorodibenzo-*p*-dioxin	3	-3363.869	-1.684	0.103	0.591
1,2,3,7,8-pentachlorodibenzo-*p*-dioxin	3	-3363.899	-0.704	0.030	0.599
1,2,3,7,9-pentachlorodibenzo-*p*-dioxin	3	-3363.949	-1.391	0.076	0.742
1,2,3,8,9-pentachlorodibenzo-*p*-dioxin	3	-3363.821	-2.516	0.117	1.826
1,2,4,6,7-pentachlorodibenzo-*p*-dioxin	3	-3363.872	0.759	-0.019	-0.960
1,2,4,6,8-pentachlorodibenzo-*p*-dioxin	3	-3364.000	-0.163	0.010	0.092
1,2,4,7,8-pentachlorodibenzo-*p*-dioxin	3	-3363.949	-0.009	-0.017	0.486
1,2,4,7,9-pentachlorodibenzo-*p*-dioxin	3	-3364.002	-0.695	0.028	0.653
1,2,4,8,9-pentachlorodibenzo-*p*-dioxin	3	-3363.872	-1.800	0.060	1.933
1-bromo-2,3,4,6-tetrachlorodibenzo-*p*-dioxin	3	-3337.133	-0.246	0.074	-6.461
1-bromo-2,3,4,7-tetrachlorodibenzo-*p*-dioxin	3	-3337.223	0.086	0.039	-6.177
1-bromo-2,3,4,8-tetrachlorodibenzo-*p*-dioxin	3	-3337.222	-1.174	0.052	-4.080
1-bromo-2,3,4,9-tetrachlorodibenzo-*p*-dioxin	3	-3337.133	-2.143	0.102	-3.523
1-bromo-2,3,6,7-tetrachlorodibenzo-*p*-dioxin	3	-3337.235	0.194	0.008	-5.578
1-bromo-2,3,6,8-tetrachlorodibenzo-*p*-dioxin	3	-3337.364	-0.731	0.042	-4.650
1-bromo-2,3,6,9-tetrachlorodibenzo-*p*-dioxin	3	-3337.275	-1.594	0.093	-4.323
1-bromo-2,3,7,8-tetrachlorodibenzo-*p*-dioxin	3	-3337.306	-0.620	0.024	-4.408
1-bromo-2,3,7,9-tetrachlorodibenzo-*p*-dioxin	3	-3337.357	-1.305	0.067	-4.191
1-bromo-2,3,8,9-tetrachlorodibenzo-*p*-dioxin	3	-3337.227	-2.433	0.106	-3.049
1-bromo-2,4,6,7-tetrachlorodibenzo-*p*-dioxin	3	-3337.287	0.829	-0.020	-6.069
1-bromo-2,4,6,8-tetrachlorodibenzo-*p*-dioxin	3	-3337.413	-0.095	0.010	-5.038
1-bromo-2,4,6,9-tetrachlorodibenzo-*p*-dioxin	3	-3337.326	-0.954	0.055	-4.564
1-bromo-2,4,7,8-tetrachlorodibenzo-*p*-dioxin	3	-3337.363	0.055	-0.018	-4.611
1-bromo-2,4,7,9-tetrachlorodibenzo-*p*-dioxin	3	-3337.416	-0.628	0.023	-4.346
1-bromo-2,4,8,9-tetrachlorodibenzo-*p*-dioxin	3	-3337.285	-1.737	0.057	-3.110
1-bromo-2,6,7,8-tetrachlorodibenzo-*p*-dioxin	3	-3337.240	-0.030	-0.010	-4.677
1-bromo-2,6,7,9-tetrachlorodibenzo-*p*-dioxin	3	-3337.285	-0.679	0.037	-4.635
1-bromo-2,6,8,9-tetrachlorodibenzo-*p*-dioxin	3	-3337.283	-1.454	0.077	-4.177
1-bromo-2,7,8,9-tetrachlorodibenzo-*p*-dioxin	3	-3337.233	-1.358	0.054	-3.775
1-bromo-3,4,6,7-tetrachlorodibenzo-*p*-dioxin	3	-3337.311	1.590	-0.039	-7.041
1-bromo-3,4,6,8-tetrachlorodibenzo-*p*-dioxin	3	-3337.440	0.666	-0.020	-5.725
1-bromo-3,4,6,9-tetrachlorodibenzo-*p*-dioxin	3	-3337.351	-0.199	0.032	-5.420
1-bromo-3,4,7,8-tetrachlorodibenzo-*p*-dioxin	3	-3337.388	0.819	-0.041	-5.485
1-bromo-3,4,7,9-tetrachlorodibenzo-*p*-dioxin	3	-3337.439	0.127	-0.001	-5.177
1-bromo-3,4,8,9-tetrachlorodibenzo-*p*-dioxin	3	-3337.310	-0.974	0.044	-4.242
1-bromo-3,6,7,8-tetrachlorodibenzo-*p*-dioxin	3	-3337.393	0.884	-0.055	-5.246
1-bromo-3,6,7,9-tetrachlorodibenzo-*p*-dioxin	3	-3337.435	0.230	-0.013	-5.065
1-bromo-3,6,8,9-tetrachlorodibenzo-*p*-dioxin	3	-3337.437	-0.540	0.029	-4.668
1-bromo-3,7,8,9-tetrachlorodibenzo-*p*-dioxin	3	-3337.385	-0.432	0.012	-4.445
1-bromo-4,6,7,8-tetrachlorodibenzo-*p*-dioxin	3	-3337.311	1.732	-0.114	-5.368
1-bromo-4,6,7,9-tetrachlorodibenzo-*p*-dioxin	3	-3337.356	1.076	-0.067	-5.312
1-bromo-4,6,8,9-tetrachlorodibenzo-*p*-dioxin	3	-3337.355	0.309	-0.030	-4.791

1-bromo-4,7,8,9-tetrachlorodibenzo-*p*-dioxin	3	-3337.311	0.456	-0.056	-4.408
1-bromo-6,7,8,9-tetrachlorodibenzo-*p*-dioxin	3	-3337.165	0.369	-0.030	-4.943
2-bromo-1,3,4,6-tetrachlorodibenzo-*p*-dioxin	3	-3337.095	-0.268	0.057	-5.984
2-bromo-1,3,4,7-tetrachlorodibenzo-*p*-dioxin	3	-3337.183	0.068	0.030	-5.916
2-bromo-1,3,4,8-tetrachlorodibenzo-*p*-dioxin	3	-3337.184	-1.187	0.049	-3.984
2-bromo-1,3,4,9-tetrachlorodibenzo-*p*-dioxin	3	-3337.094	-2.152	0.096	-3.357
2-bromo-1,3,6,7-tetrachlorodibenzo-*p*-dioxin	3	-3337.203	0.157	0.007	-5.487
2-bromo-1,3,6,8-tetrachlorodibenzo-*p*-dioxin	3	-3337.333	-0.765	0.042	-4.590
2-bromo-1,3,6,9-tetrachlorodibenzo-*p*-dioxin	3	-3337.243	-1.627	0.091	-4.214
2-bromo-1,3,7,8-tetrachlorodibenzo-*p*-dioxin	3	-3337.273	-0.648	0.026	-4.412
2-bromo-1,3,7,9-tetrachlorodibenzo-*p*-dioxin	3	-3337.323	-1.331	0.071	-4.251
2-bromo-1,3,8,9-tetrachlorodibenzo-*p*-dioxin	3	-3337.195	-2.457	0.105	-2.983
2-bromo-1,4,6,7-tetrachlorodibenzo-*p*-dioxin	3	-3337.279	0.813	-0.020	-6.039
2-bromo-1,4,6,8-tetrachlorodibenzo-*p*-dioxin	3	-3337.408	-0.106	0.006	-4.914
2-bromo-1,4,6,9-tetrachlorodibenzo-*p*-dioxin	3	-3337.320	-0.966	0.055	-4.542
2-bromo-1,4,7,8-tetrachlorodibenzo-*p*-dioxin	3	-3337.356	0.051	-0.018	-4.604
2-bromo-1,4,7,9-tetrachlorodibenzo-*p*-dioxin	3	-3337.408	-0.633	0.022	-4.312
2-bromo-1,4,8,9-tetrachlorodibenzo-*p*-dioxin	3	-3337.278	-1.738	0.056	-3.084
2-bromo-1,6,7,8-tetrachlorodibenzo-*p*-dioxin	3	-3337.240	-0.052	-0.005	-4.765
2-bromo-1,6,7,9-tetrachlorodibenzo-*p*-dioxin	3	-3337.283	-0.703	0.043	-4.745
2-bromo-1,6,8,9-tetrachlorodibenzo-*p*-dioxin	3	-3337.283	-1.477	0.083	-4.288
2-bromo-1,7,8,9-tetrachlorodibenzo-*p*-dioxin	3	-3337.231	-1.372	0.063	-3.982
2-bromo-3,4,6,7-tetrachlorodibenzo-*p*-dioxin	3	-3337.223	1.468	-0.032	-7.003
2-bromo-3,4,6,8-tetrachlorodibenzo-*p*-dioxin	3	-3337.353	0.534	-0.005	-5.876
2-bromo-3,4,6,9-tetrachlorodibenzo-*p*-dioxin	3	-3337.271	-0.367	0.038	-5.267
2-bromo-3,4,7,8-tetrachlorodibenzo-*p*-dioxin	3	-3337.301	0.693	-0.020	-5.803
2-bromo-3,4,7,9-tetrachlorodibenzo-*p*-dioxin	3	-3337.359	-0.036	0.014	-5.267
2-bromo-3,4,8,9-tetrachlorodibenzo-*p*-dioxin	3	-3337.231	-1.140	0.051	-4.118
2-bromo-3,6,7,8-tetrachlorodibenzo-*p*-dioxin	3	-3337.306	0.738	-0.039	-5.396
2-bromo-3,6,7,9-tetrachlorodibenzo-*p*-dioxin	3	-3337.355	0.046	-0.001	-5.036
2-bromo-3,6,8,9-tetrachlorodibenzo-*p*-dioxin	3	-3337.356	-0.727	0.043	-4.685
2-bromo-3,7,8,9-tetrachlorodibenzo-*p*-dioxin	3	-3337.305	-0.613	0.031	-4.603
2-bromo-4,6,7,8-tetrachlorodibenzo-*p*-dioxin	3	-3337.379	1.441	-0.073	-5.857
2-bromo-4,6,7,9-tetrachlorodibenzo-*p*-dioxin	3	-3337.430	0.746	-0.035	-5.492
2-bromo-4,6,8,9-tetrachlorodibenzo-*p*-dioxin	3	-3337.429	-0.020	0.002	-4.973
2-bromo-4,7,8,9-tetrachlorodibenzo-*p*-dioxin	3	-3337.386	0.134	-0.017	-4.785
2-bromo-6,7,8,9-tetrachlorodibenzo-*p*-dioxin	3	-3337.246	0.028	-0.001	-5.022
1,2-dibromo-3,4,6-trichlorodibenzo-*p*-dioxin	3	-3310.619	-0.245	0.058	-11.033
1,2-dibromo-3,4,7-trichlorodibenzo-*p*-dioxin	3	-3310.708	0.088	0.029	-10.907
1,2-dibromo-3,4,8-trichlorodibenzo-*p*-dioxin	3	-3310.708	-1.166	0.047	-8.951
1,2-dibromo-3,4,9-trichlorodibenzo-*p*-dioxin	3	-3310.618	-2.131	0.098	-8.428
1,2-dibromo-3,6,7-trichlorodibenzo-*p*-dioxin	3	-3310.724	0.190	0.008	-10.556
1,2-dibromo-3,6,8-trichlorodibenzo-*p*-dioxin	3	-3310.854	-0.729	0.040	-9.587
1,2-dibromo-3,6,9-trichlorodibenzo-*p*-dioxin	3	-3310.764	-1.589	0.089	-9.214

1,2-dibromo-3,7,8-trichlorodibenzo-*p*-dioxin	3	-3310.794	-0.618	0.024	-9.397
1,2-dibromo-3,7,9-trichlorodibenzo-*p*-dioxin	3	-3310.844	-1.299	0.065	-9.136
1,2-dibromo-3,8,9-trichlorodibenzo-*p*-dioxin	3	-3310.716	-2.424	0.099	-7.870
1,2-dibromo-4,6,7-trichlorodibenzo-*p*-dioxin	3	-3310.806	0.843	-0.020	-11.075
1,2-dibromo-4,6,8-trichlorodibenzo-*p*-dioxin	3	-3310.933	-0.075	0.005	-9.926
1,2-dibromo-4,6,9-trichlorodibenzo-*p*-dioxin	3	-3310.845	-0.933	0.050	-9.454
1,2-dibromo-4,7,8-trichlorodibenzo-*p*-dioxin	3	-3310.882	0.077	-0.018	-9.632
1,2-dibromo-4,7,9-trichlorodibenzo-*p*-dioxin	3	-3310.934	-0.605	0.023	-9.370
1,2-dibromo-4,8,9-trichlorodibenzo-*p*-dioxin	3	-3310.804	-1.711	0.050	-7.958
1,2-dibromo-6,7,8-trichlorodibenzo-*p*-dioxin	3	-3310.764	-0.020	-0.008	-9.727
1,2-dibromo-6,7,9-trichlorodibenzo-*p*-dioxin	3	-3310.807	-0.668	0.034	-9.557
1,2-dibromo-6,8,9-trichlorodibenzo-*p*-dioxin	3	-3310.806	-1.440	0.075	-9.131
1,2-dibromo-7,8,9-trichlorodibenzo-*p*-dioxin	3	-3310.755	-1.343	0.053	-8.756
1,3-dibromo-2,4,6-trichlorodibenzo-*p*-dioxin	3	-3310.492	-0.261	0.070	-11.338
1,3-dibromo-2,4,7-trichlorodibenzo-*p*-dioxin	3	-3310.583	0.075	0.035	-11.061
1,3-dibromo-2,4,8-trichlorodibenzo-*p*-dioxin	3	-3310.580	-1.179	0.053	-9.106
1,3-dibromo-2,4,9-trichlorodibenzo-*p*-dioxin	3	-3310.493	-2.147	0.102	-8.525
1,3-dibromo-2,6,7-trichlorodibenzo-*p*-dioxin	3	-3310.629	0.172	0.013	-10.669
1,3-dibromo-2,6,8-trichlorodibenzo-*p*-dioxin	3	-3310.756	-0.746	0.042	-9.624
1,3-dibromo-2,6,9-trichlorodibenzo-*p*-dioxin	3	-3310.668	-1.608	0.096	-9.377
1,3-dibromo-2,7,8-trichlorodibenzo-*p*-dioxin	3	-3310.699	-0.636	0.025	-9.406
1,3-dibromo-2,7,9-trichlorodibenzo-*p*-dioxin	3	-3310.751	-1.319	0.070	-9.245
1,3-dibromo-2,8,9-trichlorodibenzo-*p*-dioxin	3	-3310.620	-2.440	0.104	-7.987
1,3-dibromo-4,6,7-trichlorodibenzo-*p*-dioxin	3	-3310.721	1.578	-0.043	-11.914
1,3-dibromo-4,6,8-trichlorodibenzo-*p*-dioxin	3	-3310.849	0.657	-0.013	-10.889
1,3-dibromo-4,6,9-trichlorodibenzo-*p*-dioxin	3	-3310.761	-0.206	0.028	-10.303
1,3-dibromo-4,7,8-trichlorodibenzo-*p*-dioxin	3	-3310.798	0.812	-0.035	-10.627
1,3-dibromo-4,7,9-trichlorodibenzo-*p*-dioxin	3	-3310.850	0.122	0.005	-10.323
1,3-dibromo-4,8,9-trichlorodibenzo-*p*-dioxin	3	-3310.720	-0.976	0.042	-9.186
1,3-dibromo-6,7,8-trichlorodibenzo-*p*-dioxin	3	-3310.824	0.871	-0.056	-10.191
1,3-dibromo-6,7,9-trichlorodibenzo-*p*-dioxin	3	-3310.868	0.218	-0.013	-10.038
1,3-dibromo-6,8,9-trichlorodibenzo-*p*-dioxin	3	-3310.868	-0.547	0.027	-9.598
1,3-dibromo-7,8,9-trichlorodibenzo-*p*-dioxin	3	-3310.817	-0.440	0.011	-9.399
1,4-dibromo-2,3,6-trichlorodibenzo-*p*-dioxin	3	-3310.537	-0.338	0.085	-11.570
1,4-dibromo-2,3,7-trichlorodibenzo-*p*-dioxin	3	-3310.627	0.005	0.036	-10.944
1,4-dibromo-2,6,7-trichlorodibenzo-*p*-dioxin	3	-3310.720	0.739	-0.007	-11.228
1,4-dibromo-2,6,8-trichlorodibenzo-*p*-dioxin	3	-3310.848	-0.186	0.009	-9.832
1,4-dibromo-2,6,9-trichlorodibenzo-*p*-dioxin	3	-3310.760	-1.046	0.060	-9.511
1,4-dibromo-2,7,8-trichlorodibenzo-*p*-dioxin	3	-3310.797	-0.028	-0.018	-9.446
1,4-dibromo-2,7,9-trichlorodibenzo-*p*-dioxin	3	-3310.850	-0.711	0.032	-9.414
1,4-dibromo-2,8,9-trichlorodibenzo-*p*-dioxin	3	-3310.720	-1.822	0.050	-7.760
1,4-dibromo-6,7,8-trichlorodibenzo-*p*-dioxin	3	-3310.750	1.659	-0.108	-10.377
1,4-dibromo-6,7,9-trichlorodibenzo-*p*-dioxin	3	-3310.794	1.001	-0.068	-10.136
1,6-dibromo-2,3,4-trichlorodibenzo-*p*-dioxin	3	-3310.574	-0.292	0.068	-11.211

1,6-dibromo-2,3,7-trichlorodibenzo-*p*-dioxin	3	-3310.649	0.119	0.010	-10.485
1,6-dibromo-2,3,8-trichlorodibenzo-*p*-dioxin	3	-3310.800	-0.794	0.048	-9.679
1,6-dibromo-2,3,9-trichlorodibenzo-*p*-dioxin	3	-3310.719	-1.642	0.100	-9.406
1,6-dibromo-2,4,7-trichlorodibenzo-*p*-dioxin	3	-3310.700	0.751	-0.019	-10.943
1,6-dibromo-2,4,8-trichlorodibenzo-*p*-dioxin	3	-3310.850	-0.161	0.011	-9.932
1,6-dibromo-2,4,9-trichlorodibenzo-*p*-dioxin	3	-3310.770	-1.005	0.063	-9.666
1,6-dibromo-2,8,9-trichlorodibenzo-*p*-dioxin	3	-3310.723	-1.510	0.095	-9.530
1,6-dibromo-3,4,8-trichlorodibenzo-*p*-dioxin	3	-3310.875	0.599	-0.018	-10.642
1,6-dibromo-3,4,9-trichlorodibenzo-*p*-dioxin	3	-3310.794	-0.249	0.043	-10.603
1,7-dibromo-2,3,4-trichlorodibenzo-*p*-dioxin	3	-3310.655	0.047	0.032	-10.916
1,7-dibromo-2,3,6-trichlorodibenzo-*p*-dioxin	3	-3310.647	0.143	0.011	-10.557
1,7-dibromo-2,3,8-trichlorodibenzo-*p*-dioxin	3	-3310.713	-0.653	0.027	-9.422
1,7-dibromo-2,3,9-trichlorodibenzo-*p*-dioxin	3	-3310.785	-1.353	0.073	-9.251
1,7-dibromo-2,4,6-trichlorodibenzo-*p*-dioxin	3	-3310.697	0.776	-0.019	-10.992
1,7-dibromo-2,4,8-trichlorodibenzo-*p*-dioxin	3	-3310.769	0.019	-0.019	-9.516
1,7-dibromo-2,4,9-trichlorodibenzo-*p*-dioxin	3	-3310.844	-0.678	0.028	-9.375
1,7-dibromo-2,6,8-trichlorodibenzo-*p*-dioxin	3	-3310.615	-0.080	-0.007	-9.665
1,7-dibromo-2,6,9-trichlorodibenzo-*p*-dioxin	3	-3310.692	-0.733	0.041	-9.635
1,7-dibromo-2,8,9-trichlorodibenzo-*p*-dioxin	3	-3310.635	-1.394	0.062	-8.914
1,7-dibromo-3,4,6-trichlorodibenzo-*p*-dioxin	3	-3310.722	1.537	-0.044	-11.808
1,7-dibromo-3,4,8-trichlorodibenzo-*p*-dioxin	3	-3310.795	0.782	-0.043	-10.362
1,7-dibromo-3,4,9-trichlorodibenzo-*p*-dioxin	3	-3310.867	0.077	0.002	-10.155
1,7-dibromo-3,6,8-trichlorodibenzo-*p*-dioxin	3	-3310.768	0.831	-0.054	-10.176
1,7-dibromo-3,6,9-trichlorodibenzo-*p*-dioxin	3	-3310.843	0.174	-0.009	-10.061
1,7-dibromo-3,8,9-trichlorodibenzo-*p*-dioxin	3	-3310.788	-0.472	0.016	-9.472
1,7-dibromo-4,6,8-trichlorodibenzo-*p*-dioxin	3	-3310.686	1.676	-0.112	-10.318
1,7-dibromo-4,6,9-trichlorodibenzo-*p*-dioxin	3	-3310.762	1.018	-0.070	-10.123
1,7-dibromo-4,8,9-trichlorodibenzo-*p*-dioxin	3	-3310.713	0.413	-0.056	-9.327
1,7-dibromo-6,8,9-trichlorodibenzo-*p*-dioxin	3	-3310.535	0.318	-0.033	-9.774
1,8-dibromo-2,3,4-trichlorodibenzo-*p*-dioxin	3	-3310.655	-1.160	0.057	-9.233
1,8-dibromo-2,3,6-trichlorodibenzo-*p*-dioxin	3	-3310.792	-0.712	0.042	-9.683
1,8-dibromo-2,3,7-trichlorodibenzo-*p*-dioxin	3	-3310.712	-0.589	0.025	-9.494
1,8-dibromo-2,3,9-trichlorodibenzo-*p*-dioxin	3	-3310.638	-2.415	0.112	-8.239
1,8-dibromo-2,4,6-trichlorodibenzo-*p*-dioxin	3	-3310.842	-0.077	0.008	-10.018
1,8-dibromo-2,4,7-trichlorodibenzo-*p*-dioxin	3	-3310.771	0.084	-0.016	-9.719
1,8-dibromo-2,4,9-trichlorodibenzo-*p*-dioxin	3	-3310.697	-1.722	0.063	-8.295
1,8-dibromo-2,6,7-trichlorodibenzo-*p*-dioxin	3	-3310.643	-0.003	0.001	-10.016
1,8-dibromo-2,6,9-trichlorodibenzo-*p*-dioxin	3	-3310.691	-1.433	0.083	-9.374
1,8-dibromo-2,7,9-trichlorodibenzo-*p*-dioxin	3	-3310.608	-1.328	0.064	-9.099
1,8-dibromo-3,4,6-trichlorodibenzo-*p*-dioxin	3	-3310.868	0.679	-0.015	-10.876
1,8-dibromo-3,4,7-trichlorodibenzo-*p*-dioxin	3	-3310.794	0.841	-0.037	-10.632
1,8-dibromo-3,4,9-trichlorodibenzo-*p*-dioxin	3	-3310.722	-0.963	0.040	-9.159
1,8-dibromo-3,6,7-trichlorodibenzo-*p*-dioxin	3	-3310.794	0.903	-0.054	-10.310
1,8-dibromo-3,6,9-trichlorodibenzo-*p*-dioxin	3	-3310.843	-0.522	0.035	-9.859

1,8-dibromo-3,7,9-trichlorodibenzo-*p*-dioxin	3	-3310.759	-0.407	0.013	-9.526
1,8-dibromo-4,6,7-trichlorodibenzo-*p*-dioxin	3	-3310.714	1.752	-0.110	-10.511
1,8-dibromo-4,6,9-trichlorodibenzo-*p*-dioxin	3	-3310.762	0.325	-0.021	-10.056
1,8-dibromo-4,7,9-trichlorodibenzo-*p*-dioxin	3	-3310.687	0.479	-0.044	-9.771
1,8-dibromo-6,7,9-trichlorodibenzo-*p*-dioxin	3	-3310.534	0.389	-0.033	-9.911
1,9-dibromo-2,3,4-trichlorodibenzo-*p*-dioxin	3	-3310.574	-2.070	0.099	-8.580
1,9-dibromo-2,3,6-trichlorodibenzo-*p*-dioxin	3	-3310.719	-1.534	0.092	-9.407
1,9-dibromo-2,3,7-trichlorodibenzo-*p*-dioxin	3	-3310.793	-1.216	0.067	-9.358
1,9-dibromo-2,3,8-trichlorodibenzo-*p*-dioxin	3	-3310.641	-2.346	0.111	-8.346
1,9-dibromo-2,4,6-trichlorodibenzo-*p*-dioxin	3	-3310.769	-0.893	0.054	-9.649
1,9-dibromo-2,4,7-trichlorodibenzo-*p*-dioxin	3	-3310.852	-0.539	0.021	-9.461
1,9-dibromo-2,4,8-trichlorodibenzo-*p*-dioxin	3	-3310.698	-1.648	0.062	-8.412
1,9-dibromo-2,6,7-trichlorodibenzo-*p*-dioxin	3	-3310.724	-0.603	0.036	-9.751
1,9-dibromo-3,4,6-trichlorodibenzo-*p*-dioxin	3	-3310.795	-0.136	0.026	-10.380
1,9-dibromo-3,4,7-trichlorodibenzo-*p*-dioxin	3	-3310.875	0.218	-0.007	-10.192
2,3-dibromo-1,4,6-trichlorodibenzo-*p*-dioxin	3	-3310.584	-0.297	0.058	-10.939
2,3-dibromo-1,4,7-trichlorodibenzo-*p*-dioxin	3	-3310.673	0.043	0.027	-10.775
2,3-dibromo-1,6,7-trichlorodibenzo-*p*-dioxin	3	-3310.724	0.126	0.009	-10.458
2,3-dibromo-1,6,8-trichlorodibenzo-*p*-dioxin	3	-3310.854	-0.791	0.044	-9.571
2,3-dibromo-1,6,9-trichlorodibenzo-*p*-dioxin	3	-3310.765	-1.652	0.094	-9.222
2,3-dibromo-1,7,8-trichlorodibenzo-*p*-dioxin	3	-3310.795	-0.673	0.027	-9.368
2,3-dibromo-1,7,9-trichlorodibenzo-*p*-dioxin	3	-3310.846	-1.356	0.070	-9.156
2,3-dibromo-1,8,9-trichlorodibenzo-*p*-dioxin	3	-3310.717	-2.475	0.106	-7.953
2,3-dibromo-6,7,8-trichlorodibenzo-*p*-dioxin	3	-3310.829	0.712	-0.038	-10.350
2,3-dibromo-6,7,9-trichlorodibenzo-*p*-dioxin	3	-3310.879	0.022	0.003	-10.072
2,7-dibromo-1,3,4-trichlorodibenzo-*p*-dioxin	3	-3310.615	0.029	0.033	-10.914
2,7-dibromo-1,3,6-trichlorodibenzo-*p*-dioxin	3	-3310.615	0.105	0.008	-10.412
2,7-dibromo-1,3,8-trichlorodibenzo-*p*-dioxin	3	-3310.681	-0.682	0.029	-9.424
2,7-dibromo-1,3,9-trichlorodibenzo-*p*-dioxin	3	-3310.752	-1.381	0.075	-9.255
2,7-dibromo-1,4,6-trichlorodibenzo-*p*-dioxin	3	-3310.690	0.760	-0.018	-10.988
2,7-dibromo-1,4,8-trichlorodibenzo-*p*-dioxin	3	-3310.763	0.013	-0.018	-9.531
2,7-dibromo-1,4,9-trichlorodibenzo-*p*-dioxin	3	-3310.836	-0.684	0.028	-9.365
2,7-dibromo-1,8,9-trichlorodibenzo-*p*-dioxin	3	-3310.635	-1.410	0.071	-9.117
2,7-dibromo-3,4,8-trichlorodibenzo-*p*-dioxin	3	-3310.708	0.654	-0.020	-10.728
2,7-dibromo-3,4,9-trichlorodibenzo-*p*-dioxin	3	-3310.787	-0.090	0.017	-10.237
2,8-dibromo-1,3,4-trichlorodibenzo-*p*-dioxin	3	-3310.616	-1.174	0.049	-9.005
2,8-dibromo-1,3,6-trichlorodibenzo-*p*-dioxin	3	-3310.761	-0.747	0.042	-9.621
2,8-dibromo-1,3,7-trichlorodibenzo-*p*-dioxin	3	-3310.679	-0.618	0.025	-9.444
2,8-dibromo-1,4,6-trichlorodibenzo-*p*-dioxin	3	-3310.836	-0.090	0.006	-9.942
2,8-dibromo-1,4,7-trichlorodibenzo-*p*-dioxin	3	-3310.763	0.079	-0.017	-9.685
2,8-dibromo-1,4,9-trichlorodibenzo-*p*-dioxin	3	-3310.690	-1.724	0.056	-8.110
2,8-dibromo-1,6,7-trichlorodibenzo-*p*-dioxin	3	-3310.642	-0.027	-0.003	-9.866
2,8-dibromo-3,4,6-trichlorodibenzo-*p*-dioxin	3	-3310.782	0.547	-0.006	-10.871
2,8-dibromo-3,4,7-trichlorodibenzo-*p*-dioxin	3	-3310.708	0.715	-0.022	-10.794

1,2,3-tribromo-4,6-dichlorodibenzo-*p*-dioxin	3	-3284.110	-0.262	0.063	-16.115
1,2,3-tribromo-4,7-dichlorodibenzo-*p*-dioxin	3	-3284.200	0.075	0.029	-15.866
1,2,3-tribromo-4,8-dichlorodibenzo-*p*-dioxin	3	-3284.199	-1.175	0.047	-13.919
1,2,3-tribromo-4,9-dichlorodibenzo-*p*-dioxin	3	-3284.110	-2.139	0.094	-13.293
1,2,3-tribromo-6,7-dichlorodibenzo-*p*-dioxin	3	-3284.248	0.168	0.009	-15.518
1,2,3-tribromo-6,8-dichlorodibenzo-*p*-dioxin	3	-3284.376	-0.746	0.040	-14.533
1,2,3-tribromo-6,9-dichlorodibenzo-*p*-dioxin	3	-3284.287	-1.605	0.089	-14.162
1,2,3-tribromo-7,8-dichlorodibenzo-*p*-dioxin	3	-3284.318	-0.634	0.024	-14.344
1,2,3-tribromo-7,9-dichlorodibenzo-*p*-dioxin	3	-3284.369	-1.314	0.067	-14.138
1,2,3-tribromo-8,9-dichlorodibenzo-*p*-dioxin	3	-3284.239	-2.434	0.098	-12.803
1,2,4-tribromo-3,6-dichlorodibenzo-*p*-dioxin	3	-3284.013	-0.339	0.069	-16.140
1,2,4-tribromo-3,7-dichlorodibenzo-*p*-dioxin	3	-3284.102	0.005	0.036	-15.931
1,2,4-tribromo-3,8-dichlorodibenzo-*p*-dioxin	3	-3284.103	-1.252	0.047	-13.788
1,2,4-tribromo-3,9-dichlorodibenzo-*p*-dioxin	3	-3284.012	-2.218	0.094	-13.159
1,2,4-tribromo-6,7-dichlorodibenzo-*p*-dioxin	3	-3284.243	0.752	-0.008	-16.206
1,2,4-tribromo-6,8-dichlorodibenzo-*p*-dioxin	3	-3284.372	-0.168	0.007	-14.793
1,2,4-tribromo-6,9-dichlorodibenzo-*p*-dioxin	3	-3284.283	-1.027	0.056	-14.423
1,2,4-tribromo-7,9-dichlorodibenzo-*p*-dioxin	3	-3284.371	-0.690	0.024	-14.226
1,2,4-tribromo-8,9-dichlorodibenzo-*p*-dioxin	3	-3284.242	-1.798	0.047	-12.707
1,2,6-tribromo-3,4-dichlorodibenzo-*p*-dioxin	3	-3284.060	-0.292	0.070	-16.248
1,2,6-tribromo-3,7-dichlorodibenzo-*p*-dioxin	3	-3284.137	0.113	0.008	-15.406
1,2,6-tribromo-3,8-dichlorodibenzo-*p*-dioxin	3	-3284.290	-0.795	0.045	-14.584
1,2,6-tribromo-3,9-dichlorodibenzo-*p*-dioxin	3	-3284.207	-1.639	0.094	-14.241
1,2,6-tribromo-4,7-dichlorodibenzo-*p*-dioxin	3	-3284.219	0.764	-0.019	-15.948
1,2,6-tribromo-4,8-dichlorodibenzo-*p*-dioxin	3	-3284.370	-0.143	0.007	-14.842
1,2,6-tribromo-4,9-dichlorodibenzo-*p*-dioxin	3	-3284.289	-0.986	0.056	-14.501
1,2,6-tribromo-7,8-dichlorodibenzo-*p*-dioxin	3	-3284.172	-0.110	0.000	-14.761
1,2,6-tribromo-7,9-dichlorodibenzo-*p*-dioxin	3	-3284.222	-0.739	0.044	-14.679
1,2,6-tribromo-8,9-dichlorodibenzo-*p*-dioxin	3	-3284.246	-1.498	0.089	-14.376
1,2,7-tribromo-3,4-dichlorodibenzo-*p*-dioxin	3	-3284.140	0.050	0.038	-16.063
1,2,7-tribromo-3,6-dichlorodibenzo-*p*-dioxin	3	-3284.137	0.140	0.012	-15.562
1,2,7-tribromo-3,8-dichlorodibenzo-*p*-dioxin	3	-3284.202	-0.650	0.025	-14.361
1,2,7-tribromo-3,9-dichlorodibenzo-*p*-dioxin	3	-3284.273	-1.347	0.070	-14.170
1,2,7-tribromo-4,6-dichlorodibenzo-*p*-dioxin	3	-3284.217	0.791	-0.015	-16.104
1,2,7-tribromo-4,8-dichlorodibenzo-*p*-dioxin	3	-3284.289	0.041	-0.018	-14.564
1,2,7-tribromo-4,9-dichlorodibenzo-*p*-dioxin	3	-3284.362	-0.655	0.026	-14.347
1,2,7-tribromo-6,8-dichlorodibenzo-*p*-dioxin	3	-3284.139	-0.070	-0.006	-14.689
1,2,7-tribromo-6,9-dichlorodibenzo-*p*-dioxin	3	-3284.214	-0.722	0.038	-14.557
1,2,7-tribromo-8,9-dichlorodibenzo-*p*-dioxin	3	-3284.158	-1.378	0.061	-13.898
1,2,8-tribromo-3,4-dichlorodibenzo-*p*-dioxin	3	-3284.140	-1.152	0.047	-13.974
1,2,8-tribromo-3,6-dichlorodibenzo-*p*-dioxin	3	-3284.281	-0.711	0.039	-14.592
1,2,8-tribromo-3,7-dichlorodibenzo-*p*-dioxin	3	-3284.200	-0.587	0.024	-14.457
1,2,8-tribromo-3,9-dichlorodibenzo-*p*-dioxin	3	-3284.126	-2.406	0.100	-12.930
1,2,8-tribromo-4,6-dichlorodibenzo-*p*-dioxin	3	-3284.362	-0.057	0.007	-15.010

1,2,8-tribromo-4,7-dichlorodibenzo-*p*-dioxin	3	-3284.289	0.105	-0.018	-14.687
1,2,8-tribromo-4,9-dichlorodibenzo-*p*-dioxin	3	-3284.216	-1.696	0.055	-13.117
1,2,8-tribromo-6,7-dichlorodibenzo-*p*-dioxin	3	-3284.166	0.006	-0.008	-14.779
1,2,8-tribromo-6,9-dichlorodibenzo-*p*-dioxin	3	-3284.213	-1.419	0.076	-14.198
1,2,8-tribromo-7,9-dichlorodibenzo-*p*-dioxin	3	-3284.130	-1.313	0.056	-13.899
1,2,9-tribromo-3,4-dichlorodibenzo-*p*-dioxin	3	-3284.059	-2.056	0.092	-13.411
1,2,9-tribromo-3,6-dichlorodibenzo-*p*-dioxin	3	-3284.208	-1.527	0.084	-14.198
1,2,9-tribromo-3,7-dichlorodibenzo-*p*-dioxin	3	-3284.281	-1.209	0.063	-14.253
1,2,9-tribromo-3,8-dichlorodibenzo-*p*-dioxin	3	-3284.130	-2.335	0.105	-13.197
1,2,9-tribromo-4,6-dichlorodibenzo-*p*-dioxin	3	-3284.289	-0.870	0.047	-14.491
1,2,9-tribromo-4,7-dichlorodibenzo-*p*-dioxin	3	-3284.370	-0.514	0.022	-14.515
1,2,9-tribromo-4,8-dichlorodibenzo-*p*-dioxin	3	-3284.217	-1.621	0.057	-13.313
1,2,9-tribromo-6,7-dichlorodibenzo-*p*-dioxin	3	-3284.246	-0.590	0.035	-14.729
1,2,9-tribromo-6,8-dichlorodibenzo-*p*-dioxin	3	-3284.221	-1.367	0.076	-14.297
1,2,9-tribromo-7,8-dichlorodibenzo-*p*-dioxin	3	-3284.163	-1.249	0.060	-14.120
1,3,6-tribromo-2,4-dichlorodibenzo-*p*-dioxin	3	-3283.933	-0.307	0.065	-16.113
1,3,6-tribromo-2,7-dichlorodibenzo-*p*-dioxin	3	-3284.042	0.096	0.020	-15.703
1,3,6-tribromo-2,8-dichlorodibenzo-*p*-dioxin	3	-3284.192	-0.811	0.048	-14.649
1,3,6-tribromo-2,9-dichlorodibenzo-*p*-dioxin	3	-3284.112	-1.658	0.104	-14.482
1,3,6-tribromo-4,7-dichlorodibenzo-*p*-dioxin	3	-3284.134	1.500	-0.051	-16.555
1,3,6-tribromo-4,8-dichlorodibenzo-*p*-dioxin	3	-3284.285	0.590	-0.016	-15.677
1,3,6-tribromo-4,9-dichlorodibenzo-*p*-dioxin	3	-3284.204	-0.257	0.041	-15.536
1,3,6-tribromo-7,8-dichlorodibenzo-*p*-dioxin	3	-3284.232	0.780	-0.046	-15.275
1,3,6-tribromo-7,9-dichlorodibenzo-*p*-dioxin	3	-3284.282	0.149	0.001	-15.267
1,3,6-tribromo-8,9-dichlorodibenzo-*p*-dioxin	3	-3284.307	-0.606	0.043	-14.894
1,3,7-tribromo-2,4-dichlorodibenzo-*p*-dioxin	3	-3284.014	0.037	0.039	-16.088
1,3,7-tribromo-2,6-dichlorodibenzo-*p*-dioxin	3	-3284.040	0.121	0.013	-15.570
1,3,7-tribromo-2,8-dichlorodibenzo-*p*-dioxin	3	-3284.105	-0.668	0.028	-14.423
1,3,7-tribromo-2,9-dichlorodibenzo-*p*-dioxin	3	-3284.179	-1.367	0.075	-14.279
1,3,7-tribromo-4,6-dichlorodibenzo-*p*-dioxin	3	-3284.132	1.524	-0.048	-16.679
1,3,7-tribromo-4,8-dichlorodibenzo-*p*-dioxin	3	-3284.204	0.774	-0.044	-15.321
1,3,7-tribromo-4,9-dichlorodibenzo-*p*-dioxin	3	-3284.278	0.071	0.000	-15.091
1,3,7-tribromo-6,8-dichlorodibenzo-*p*-dioxin	3	-3284.199	0.817	-0.054	-15.145
1,3,7-tribromo-6,9-dichlorodibenzo-*p*-dioxin	3	-3284.275	0.162	-0.010	-15.008
1,3,7-tribromo-8,9-dichlorodibenzo-*p*-dioxin	3	-3284.220	-0.479	0.014	-14.403
1,3,8-tribromo-2,4-dichlorodibenzo-*p*-dioxin	3	-3284.013	-1.165	0.055	-14.181
1,3,8-tribromo-2,6-dichlorodibenzo-*p*-dioxin	3	-3284.184	-0.728	0.041	-14.629
1,3,8-tribromo-2,7-dichlorodibenzo-*p*-dioxin	3	-3284.106	-0.605	0.023	-14.414
1,3,8-tribromo-2,9-dichlorodibenzo-*p*-dioxin	3	-3284.032	-2.423	0.108	-13.123
1,3,8-tribromo-4,6-dichlorodibenzo-*p*-dioxin	3	-3284.277	0.670	-0.014	-15.885
1,3,8-tribromo-4,7-dichlorodibenzo-*p*-dioxin	3	-3284.205	0.835	-0.038	-15.594
1,3,8-tribromo-4,9-dichlorodibenzo-*p*-dioxin	3	-3284.132	-0.964	0.038	-14.105
1,3,8-tribromo-6,7-dichlorodibenzo-*p*-dioxin	3	-3284.226	0.890	-0.054	-15.281
1,3,8-tribromo-6,9-dichlorodibenzo-*p*-dioxin	3	-3284.274	-0.530	0.034	-14.813

1,3,8-tribromo-7,9-dichlorodibenzo-*p*-dioxin	3	-3284.192	-0.416	0.012	-14.478
1,3,9-tribromo-2,4-dichlorodibenzo-*p*-dioxin	3	-3283.934	-2.075	0.100	-13.606
1,3,9-tribromo-2,6-dichlorodibenzo-*p*-dioxin	3	-3284.112	-1.550	0.092	-14.379
1,3,9-tribromo-2,7-dichlorodibenzo-*p*-dioxin	3	-3284.187	-1.232	0.067	-14.330
1,3,9-tribromo-2,8-dichlorodibenzo-*p*-dioxin	3	-3284.034	-2.354	0.111	-13.334
1,3,9-tribromo-4,6-dichlorodibenzo-*p*-dioxin	3	-3284.204	-0.144	0.027	-15.390
1,3,9-tribromo-4,7-dichlorodibenzo-*p*-dioxin	3	-3284.286	0.212	-0.007	-15.180
1,3,9-tribromo-4,8-dichlorodibenzo-*p*-dioxin	3	-3284.134	-0.886	0.043	-14.386
1,3,9-tribromo-6,7-dichlorodibenzo-*p*-dioxin	3	-3284.307	0.294	-0.016	-15.101
1,3,9-tribromo-6,8-dichlorodibenzo-*p*-dioxin	3	-3284.283	-0.475	0.030	-14.814
1,3,9-tribromo-7,8-dichlorodibenzo-*p*-dioxin	3	-3284.225	-0.348	0.014	-14.655
1,4,6-tribromo-2,3-dichlorodibenzo-*p*-dioxin	3	-3283.979	-0.372	0.080	-16.369
1,4,6-tribromo-2,7-dichlorodibenzo-*p*-dioxin	3	-3284.135	0.672	-0.012	-15.967
1,4,6-tribromo-2,8-dichlorodibenzo-*p*-dioxin	3	-3284.285	-0.241	0.010	-14.746
1,4,6-tribromo-2,9-dichlorodibenzo-*p*-dioxin	3	-3284.205	-1.085	0.068	-14.637
1,4,6-tribromo-3,7-dichlorodibenzo-*p*-dioxin	3	-3284.134	1.441	-0.032	-16.934
1,4,6-tribromo-3,8-dichlorodibenzo-*p*-dioxin	3	-3284.287	0.525	-0.007	-15.785
1,4,6-tribromo-3,9-dichlorodibenzo-*p*-dioxin	3	-3284.204	-0.325	0.052	-15.690
1,4,6-tribromo-7,8-dichlorodibenzo-*p*-dioxin	3	-3284.159	1.578	-0.110	-15.169
1,4,6-tribromo-7,9-dichlorodibenzo-*p*-dioxin	3	-3284.209	0.940	-0.068	-15.017
1,4,6-tribromo-8,9-dichlorodibenzo-*p*-dioxin	3	-3284.234	0.184	-0.015	-14.928
1,4,7-tribromo-2,3-dichlorodibenzo-*p*-dioxin	3	-3284.059	-0.035	0.039	-15.940
1,4,7-tribromo-2,6-dichlorodibenzo-*p*-dioxin	3	-3284.131	0.685	-0.008	-16.097
1,4,7-tribromo-2,8-dichlorodibenzo-*p*-dioxin	3	-3284.204	-0.065	-0.018	-14.375
1,4,7-tribromo-2,9-dichlorodibenzo-*p*-dioxin	3	-3284.278	-0.762	0.028	-14.209
1,4,7-tribromo-3,6-dichlorodibenzo-*p*-dioxin	3	-3284.132	1.453	-0.032	-16.958
1,4,7-tribromo-3,8-dichlorodibenzo-*p*-dioxin	3	-3284.205	0.705	-0.038	-15.343
1,4,7-tribromo-3,9-dichlorodibenzo-*p*-dioxin	3	-3284.277	-0.001	0.004	-15.056
1,4,7-tribromo-6,8-dichlorodibenzo-*p*-dioxin	3	-3284.125	1.601	-0.111	-15.194
1,4,7-tribromo-6,9-dichlorodibenzo-*p*-dioxin	3	-3284.201	0.941	-0.065	-15.099
1,4,7-tribromo-8,9-dichlorodibenzo-*p*-dioxin	3	-3284.152	0.344	-0.061	-14.058
1,7,8-tribromo-2,3-dichlorodibenzo-*p*-dioxin	3	-3284.236	-0.626	0.028	-14.479
1,7,8-tribromo-2,4-dichlorodibenzo-*p*-dioxin	3	-3284.293	0.044	-0.017	-14.594
1,7,8-tribromo-2,6-dichlorodibenzo-*p*-dioxin	3	-3284.137	-0.048	-0.004	-14.784
1,7,8-tribromo-2,9-dichlorodibenzo-*p*-dioxin	3	-3284.130	-1.366	0.063	-13.978
1,7,8-tribromo-3,4-dichlorodibenzo-*p*-dioxin	3	-3284.318	0.801	-0.036	-15.559
1,7,8-tribromo-3,6-dichlorodibenzo-*p*-dioxin	3	-3284.289	0.857	-0.054	-15.205
1,7,8-tribromo-3,9-dichlorodibenzo-*p*-dioxin	3	-3284.281	-0.447	0.015	-14.479
1,7,8-tribromo-4,6-dichlorodibenzo-*p*-dioxin	3	-3284.208	1.702	-0.110	-15.399
1,7,8-tribromo-4,9-dichlorodibenzo-*p*-dioxin	3	-3284.208	0.436	-0.054	-14.407
1,7,8-tribromo-6,9-dichlorodibenzo-*p*-dioxin	3	-3284.024	0.348	-0.027	-14.973
2,3,7-tribromo-1,4-dichlorodibenzo-*p*-dioxin	3	-3284.105	0.003	0.029	-15.745
2,3,7-tribromo-1,6-dichlorodibenzo-*p*-dioxin	3	-3284.136	0.074	0.011	-15.409
2,3,7-tribromo-1,8-dichlorodibenzo-*p*-dioxin	3	-3284.202	-0.707	0.030	-14.381

2,3,7-tribromo-1,9-dichlorodibenzo-*p*-dioxin	3	-3284.275	-1.406	0.077	-14.237
2,3,7-tribromo-4,6-dichlorodibenzo-*p*-dioxin	3	-3284.128	1.383	-0.030	-16.875
2,3,7-tribromo-4,8-dichlorodibenzo-*p*-dioxin	3	-3284.201	0.632	-0.021	-15.644
2,3,7-tribromo-4,9-dichlorodibenzo-*p*-dioxin	3	-3284.281	-0.109	0.018	-15.210
2,3,7-tribromo-6,8-dichlorodibenzo-*p*-dioxin	3	-3284.203	0.656	-0.035	-15.326
2,3,7-tribromo-6,9-dichlorodibenzo-*p*-dioxin	3	-3284.286	-0.038	0.003	-14.957
2,3,7-tribromo-8,9-dichlorodibenzo-*p*-dioxin	3	-3284.231	-0.672	0.037	-14.625
1,2,3,4-tetrabromo-6-chlorodibenzo-*p*-dioxin	3	-3257.632	-0.350	0.072	-21.157
1,2,3,4-tetrabromo-7-chlorodibenzo-*p*-dioxin	3	-3257.722	-0.006	0.036	-20.870
1,2,3,6-tetrabromo-4-chlorodibenzo-*p*-dioxin	3	-3257.552	-0.311	0.068	-21.144
1,2,3,6-tetrabromo-7-chlorodibenzo-*p*-dioxin	3	-3257.661	0.091	0.010	-20.394
1,2,3,6-tetrabromo-8-chlorodibenzo-*p*-dioxin	3	-3257.812	-0.812	0.045	-19.530
1,2,3,6-tetrabromo-9-chlorodibenzo-*p*-dioxin	3	-3257.731	-1.656	0.096	-19.239
1,2,3,7-tetrabromo-4-chlorodibenzo-*p*-dioxin	3	-3257.632	0.037	0.034	-20.919
1,2,3,7-tetrabromo-6-chlorodibenzo-*p*-dioxin	3	-3257.660	0.118	0.013	-20.525
1,2,3,7-tetrabromo-8-chlorodibenzo-*p*-dioxin	3	-3257.725	-0.666	0.026	-19.335
1,2,3,7-tetrabromo-9-chlorodibenzo-*p*-dioxin	3	-3257.798	-1.363	0.071	-19.143
1,2,3,8-tetrabromo-4-chlorodibenzo-*p*-dioxin	3	-3257.632	-1.161	0.048	-18.967
1,2,3,8-tetrabromo-6-chlorodibenzo-*p*-dioxin	3	-3257.804	-0.727	0.041	-19.592
1,2,3,8-tetrabromo-7-chlorodibenzo-*p*-dioxin	3	-3257.725	-0.603	0.025	-19.431
1,2,3,8-tetrabromo-9-chlorodibenzo-*p*-dioxin	3	-3257.651	-2.416	0.100	-17.889
1,2,3,9-tetrabromo-4-chlorodibenzo-*p*-dioxin	3	-3257.551	-2.065	0.092	-18.378
1,2,3,9-tetrabromo-6-chlorodibenzo-*p*-dioxin	3	-3257.731	-1.544	0.085	-19.170
1,2,3,9-tetrabromo-7-chlorodibenzo-*p*-dioxin	3	-3257.805	-1.225	0.064	-19.227
1,2,3,9-tetrabromo-8-chlorodibenzo-*p*-dioxin	3	-3257.653	-2.346	0.106	-18.180
1,2,4,6-tetrabromo-3-chlorodibenzo-*p*-dioxin	3	-3257.455	-0.375	0.081	-21.376
1,2,4,6-tetrabromo-7-chlorodibenzo-*p*-dioxin	3	-3257.657	0.683	-0.012	-20.967
1,2,4,6-tetrabromo-8-chlorodibenzo-*p*-dioxin	3	-3257.808	-0.225	0.010	-19.756
1,2,4,6-tetrabromo-9-chlorodibenzo-*p*-dioxin	3	-3257.727	-1.068	0.061	-19.467
1,2,4,7-tetrabromo-3-chlorodibenzo-*p*-dioxin	3	-3257.534	-0.034	0.039	-20.930
1,2,4,7-tetrabromo-6-chlorodibenzo-*p*-dioxin	3	-3257.655	0.698	-0.007	-21.127
1,2,4,7-tetrabromo-8-chlorodibenzo-*p*-dioxin	3	-3257.727	-0.045	-0.018	-19.392
1,2,4,7-tetrabromo-9-chlorodibenzo-*p*-dioxin	3	-3257.799	-0.741	0.026	-19.176
1,2,4,8-tetrabromo-3-chlorodibenzo-*p*-dioxin	3	-3257.534	-1.237	0.048	-18.839
1,2,4,8-tetrabromo-6-chlorodibenzo-*p*-dioxin	3	-3257.800	-0.150	0.009	-19.876
1,2,4,8-tetrabromo-7-chlorodibenzo-*p*-dioxin	3	-3257.727	0.021	-0.017	-19.546
1,2,4,8-tetrabromo-9-chlorodibenzo-*p*-dioxin	3	-3257.654	-1.782	0.048	-17.763
1,2,4,9-tetrabromo-3-chlorodibenzo-*p*-dioxin	3	-3257.454	-2.143	0.093	-18.272
1,2,4,9-tetrabromo-6-chlorodibenzo-*p*-dioxin	3	-3257.727	-0.964	0.051	-19.408
1,2,4,9-tetrabromo-7-chlorodibenzo-*p*-dioxin	3	-3257.808	-0.599	0.020	-19.293
1,2,4,9-tetrabromo-8-chlorodibenzo-*p*-dioxin	3	-3257.656	-1.708	0.051	-17.984
1,2,6,7-tetrabromo-3-chlorodibenzo-*p*-dioxin	3	-3257.661	0.105	0.009	-20.395
1,2,6,7-tetrabromo-4-chlorodibenzo-*p*-dioxin	3	-3257.741	0.753	-0.020	-20.879
1,2,6,8-tetrabromo-3-chlorodibenzo-*p*-dioxin	3	-3257.720	-0.781	0.046	-19.633

1,2,6,8-tetrabromo-4-chlorodibenzo-*p*-dioxin	3	-3257.802	-0.130	0.010	-19.941
1,2,6,8-tetrabromo-7-chlorodibenzo-*p*-dioxin	3	-3257.565	-0.087	0.010	-20.068
1,2,6,8-tetrabromo-9-chlorodibenzo-*p*-dioxin	3	-3257.656	-1.485	0.090	-19.427
1,2,6,9-tetrabromo-3-chlorodibenzo-*p*-dioxin	3	-3257.647	-1.574	0.092	-19.309
1,2,6,9-tetrabromo-4-chlorodibenzo-*p*-dioxin	3	-3257.728	-0.919	0.052	-19.521
1,2,6,9-tetrabromo-7-chlorodibenzo-*p*-dioxin	3	-3257.657	-0.658	0.041	-19.753
1,2,6,9-tetrabromo-8-chlorodibenzo-*p*-dioxin	3	-3257.657	-1.424	0.093	-19.623
1,2,7,8-tetrabromo-3-chlorodibenzo-*p*-dioxin	3	-3257.725	-0.624	0.027	-19.442
1,2,7,8-tetrabromo-4-chlorodibenzo-*p*-dioxin	3	-3257.812	0.066	-0.018	-19.590
1,2,7,8-tetrabromo-6-chlorodibenzo-*p*-dioxin	3	-3257.660	-0.038	-0.006	-19.730
1,2,7,8-tetrabromo-9-chlorodibenzo-*p*-dioxin	3	-3257.652	-1.349	0.061	-18.938
1,2,7,9-tetrabromo-3-chlorodibenzo-*p*-dioxin	3	-3257.713	-1.256	0.069	-19.314
1,2,7,9-tetrabromo-4-chlorodibenzo-*p*-dioxin	3	-3257.801	-0.564	0.024	-19.466
1,2,7,9-tetrabromo-6-chlorodibenzo-*p*-dioxin	3	-3257.656	-0.645	0.040	-19.752
1,2,7,9-tetrabromo-8-chlorodibenzo-*p*-dioxin	3	-3257.556	-1.283	0.065	-19.187
1,2,8,9-tetrabromo-3-chlorodibenzo-*p*-dioxin	3	-3257.651	-2.320	0.104	-18.179
1,2,8,9-tetrabromo-4-chlorodibenzo-*p*-dioxin	3	-3257.740	-1.610	0.058	-18.339
1,3,6,8-tetrabromo-2-chlorodibenzo-*p*-dioxin	3	-3257.624	-0.797	0.046	-19.620
1,3,6,8-tetrabromo-4-chlorodibenzo-*p*-dioxin	3	-3257.717	0.598	-0.015	-20.714
1,3,6,9-tetrabromo-2-chlorodibenzo-*p*-dioxin	3	-3257.552	-1.595	0.100	-19.494
1,3,6,9-tetrabromo-4-chlorodibenzo-*p*-dioxin	3	-3257.644	-0.192	0.037	-20.552
1,3,6,9-tetrabromo-7-chlorodibenzo-*p*-dioxin	3	-3257.717	0.227	-0.013	-20.049
1,3,6,9-tetrabromo-8-chlorodibenzo-*p*-dioxin	3	-3257.718	-0.533	0.038	-19.905
1,3,7,8-tetrabromo-2-chlorodibenzo-*p*-dioxin	3	-3257.629	-0.642	0.028	-19.451
1,3,7,8-tetrabromo-4-chlorodibenzo-*p*-dioxin	3	-3257.728	0.794	-0.042	-20.390
1,3,7,8-tetrabromo-6-chlorodibenzo-*p*-dioxin	3	-3257.720	0.844	-0.054	-20.176
1,3,7,8-tetrabromo-9-chlorodibenzo-*p*-dioxin	3	-3257.713	-0.455	0.014	-19.433
1,3,7,9-tetrabromo-2-chlorodibenzo-*p*-dioxin	3	-3257.618	-1.279	0.072	-19.365
1,3,7,9-tetrabromo-4-chlorodibenzo-*p*-dioxin	3	-3257.717	0.161	-0.005	-20.129
1,4,6,9-tetrabromo-2-chlorodibenzo-*p*-dioxin	3	-3257.645	-1.021	0.068	-19.755
1,4,7,8-tetrabromo-2-chlorodibenzo-*p*-dioxin	3	-3257.728	-0.039	-0.017	-19.429
1,4,7,8-tetrabromo-6-chlorodibenzo-*p*-dioxin	3	-3257.647	1.628	-0.105	-20.380
2,3,7,8-tetrabromo-1-chlorodibenzo-*p*-dioxin	3	-3257.725	-0.682	0.029	-19.382
1,2,3,4,6-pentabromodibenzo-*p*-dioxin	3	-3231.074	-0.388	0.078	-26.234
1,2,3,4,7-pentabromodibenzo-*p*-dioxin	3	-3231.154	-0.046	0.039	-25.867
1,2,3,6,7-pentabromodibenzo-*p*-dioxin	3	-3231.184	0.083	0.011	-25.383
1,2,3,6,8-pentabromodibenzo-*p*-dioxin	3	-3231.244	-0.798	0.046	-24.579
1,2,3,6,9-pentabromodibenzo-*p*-dioxin	3	-3231.171	-1.591	0.093	-24.281
1,2,3,7,8-pentabromodibenzo-*p*-dioxin	3	-3231.248	-0.640	0.026	-24.364
1,2,3,7,9-pentabromodibenzo-*p*-dioxin	3	-3231.237	-1.272	0.071	-24.313
1,2,3,8,9-pentabromodibenzo-*p*-dioxin	3	-3231.175	-2.332	0.104	-23.134
1,2,4,6,7-pentabromodibenzo-*p*-dioxin	3	-3231.180	0.671	-0.012	-25.923
1,2,4,6,8-pentabromodibenzo-*p*-dioxin	3	-3231.240	-0.211	0.010	-24.779
1,2,4,6,9-pentabromodibenzo-*p*-dioxin	3	-3231.167	-1.002	0.057	-24.485

1,2,4,7,9-pentabromodibenzo-*p*-dioxin	3	-3231.239	-0.649	0.025	-24.322
1,2,4,8,9-pentabromodibenzo-*p*-dioxin	3	-3231.178	-1.696	0.052	-23.012
1,2,3,4,6,7-hexachlorodibenzo-*p*-dioxin	2	-3604.606	0.703	0.002	-2.175
1,2,3,4,7,8-hexachlorodibenzo-*p*-dioxin	2	-3604.681	-0.047	0.003	-0.738
1,2,3,6,7,8-hexachlorodibenzo-*p*-dioxin	2	-3604.692	0.000	0.000	-0.749
1,2,3,6,7,9-hexachlorodibenzo-*p*-dioxin	2	-3604.736	-0.638	0.041	-0.573
1,2,3,6,8,9-hexachlorodibenzo-*p*-dioxin	2	-3604.736	-1.399	0.080	-0.115
1,2,3,7,8,9-hexachlorodibenzo-*p*-dioxin	2	-3604.685	-1.284	0.063	0.095
1,2,4,6,7,9-hexachlorodibenzo-*p*-dioxin	2	-3604.781	0.000	0.003	-0.810
1,2,4,6,8,9-hexachlorodibenzo-*p*-dioxin	2	-3604.780	-0.752	0.033	-0.136
1-bromo-2,3,4,6,7-pentachlorodibenzo-*p*-dioxin	2	-3578.015	0.783	0.000	-7.278
1-bromo-2,3,4,6,8-pentachlorodibenzo-*p*-dioxin	2	-3578.142	-0.128	0.024	-6.117
1-bromo-2,3,4,6,9-pentachlorodibenzo-*p*-dioxin	2	-3578.055	-0.978	0.069	-5.660
1-bromo-2,3,4,7,8-pentachlorodibenzo-*p*-dioxin	2	-3578.091	0.027	0.001	-5.829
1-bromo-2,3,4,7,9-pentachlorodibenzo-*p*-dioxin	2	-3578.143	-0.647	0.039	-5.504
1-bromo-2,3,4,8,9-pentachlorodibenzo-*p*-dioxin	2	-3578.014	-1.744	0.068	-4.161
1-bromo-2,3,6,7,8-pentachlorodibenzo-*p*-dioxin	2	-3578.099	0.092	-0.004	-5.823
1-bromo-2,3,6,7,9-pentachlorodibenzo-*p*-dioxin	2	-3578.143	-0.543	0.035	-5.601
1-bromo-2,3,6,8,9-pentachlorodibenzo-*p*-dioxin	2	-3578.143	-1.305	0.072	-5.089
1-bromo-2,3,7,8,9-pentachlorodibenzo-*p*-dioxin	2	-3578.092	-1.196	0.056	-4.894
1-bromo-2,4,6,7,8-pentachlorodibenzo-*p*-dioxin	2	-3578.150	0.710	-0.039	-6.099
1-bromo-2,4,6,7,9-pentachlorodibenzo-*p*-dioxin	2	-3578.196	0.075	-0.002	-5.824
1-bromo-2,4,6,8,9-pentachlorodibenzo-*p*-dioxin	2	-3578.193	-0.679	0.030	-5.199
1-bromo-2,4,7,8,9-pentachlorodibenzo-*p*-dioxin	2	-3578.151	-0.532	0.006	-4.868
1-bromo-2,6,7,8,9-pentachlorodibenzo-*p*-dioxin	2	-3578.018	-0.622	0.030	-5.342
1-bromo-3,4,6,7,8-pentachlorodibenzo-*p*-dioxin	2	-3578.175	1.460	-0.072	-6.686
1-bromo-3,4,6,7,9-pentachlorodibenzo-*p*-dioxin	2	-3578.219	0.814	-0.033	-6.443
1-bromo-3,4,6,8,9-pentachlorodibenzo-*p*-dioxin	2	-3578.219	0.062	0.000	-5.846
1-bromo-3,4,7,8,9-pentachlorodibenzo-*p*-dioxin	2	-3578.174	0.215	-0.014	-5.787
1-bromo-3,6,7,8,9-pentachlorodibenzo-*p*-dioxin	2	-3578.169	0.276	-0.022	-5.698
1-bromo-4,6,7,8,9-pentachlorodibenzo-*p*-dioxin	2	-3578.089	1.111	-0.086	-5.665
2-bromo-1,3,4,6,7-pentachlorodibenzo-*p*-dioxin	2	-3577.975	0.757	-0.001	-7.209
2-bromo-1,3,4,6,8-pentachlorodibenzo-*p*-dioxin	2	-3578.104	-0.150	0.020	-5.978
2-bromo-1,3,4,6,9-pentachlorodibenzo-*p*-dioxin	2	-3578.016	-0.999	0.068	-5.600
2-bromo-1,3,4,7,8-pentachlorodibenzo-*p*-dioxin	2	-3578.051	0.011	0.000	-5.780
2-bromo-1,3,4,7,9-pentachlorodibenzo-*p*-dioxin	2	-3578.102	-0.663	0.038	-5.455
2-bromo-1,3,4,8,9-pentachlorodibenzo-*p*-dioxin	2	-3577.975	-1.756	0.067	-4.120
2-bromo-1,3,6,7,8-pentachlorodibenzo-*p*-dioxin	2	-3578.067	0.055	-0.003	-5.784
2-bromo-1,3,6,7,9-pentachlorodibenzo-*p*-dioxin	2	-3578.109	-0.580	0.035	-5.536
2-bromo-1,3,6,8,9-pentachlorodibenzo-*p*-dioxin	2	-3578.111	-1.340	0.073	-5.054
2-bromo-1,3,7,8,9-pentachlorodibenzo-*p*-dioxin	2	-3578.058	-1.223	0.057	-4.874
2-bromo-1,4,6,7,8-pentachlorodibenzo-*p*-dioxin	2	-3578.143	0.698	-0.039	-6.077
2-bromo-1,4,6,7,9-pentachlorodibenzo-*p*-dioxin	2	-3578.187	0.061	-0.003	-5.773
2-bromo-1,4,6,8,9-pentachlorodibenzo-*p*-dioxin	2	-3578.187	-0.691	0.029	-5.151

2-bromo-1,4,7,8,9-pentachlorodibenzo-*p*-dioxin	2	-3578.142	-0.536	0.008	-4.913
2-bromo-1,6,7,8,9-pentachlorodibenzo-*p*-dioxin	2	-3578.017	-0.645	0.036	-5.453
2-bromo-3,4,6,7,8-pentachlorodibenzo-*p*-dioxin	2	-3578.087	1.325	-0.052	-6.961
2-bromo-3,4,6,7,9-pentachlorodibenzo-*p*-dioxin	2	-3578.138	0.642	-0.020	-6.463
2-bromo-3,4,6,8,9-pentachlorodibenzo-*p*-dioxin	2	-3578.138	-0.114	0.013	-5.859
2-bromo-3,4,7,8,9-pentachlorodibenzo-*p*-dioxin	2	-3578.093	0.045	-0.002	-5.785
2-bromo-3,6,7,8,9-pentachlorodibenzo-*p*-dioxin	2	-3578.088	0.085	-0.003	-5.838
2-bromo-4,6,7,8,9-pentachlorodibenzo-*p*-dioxin	2	-3578.162	0.777	-0.047	-6.018
1,2-dibromo-3,4,6,7-tetrachlorodibenzo-*p*-dioxin	2	-3551.499	0.784	0.001	-12.292
1,2-dibromo-3,4,6,8-tetrachlorodibenzo-*p*-dioxin	2	-3551.628	-0.121	0.021	-11.038
1,2-dibromo-3,4,6,9-tetrachlorodibenzo-*p*-dioxin	2	-3551.539	-0.968	0.063	-10.509
1,2-dibromo-3,4,7,8-tetrachlorodibenzo-*p*-dioxin	2	-3551.575	0.035	0.002	-10.856
1,2-dibromo-3,4,7,9-tetrachlorodibenzo-*p*-dioxin	2	-3551.626	-0.637	0.038	-10.483
1,2-dibromo-3,4,8,9-tetrachlorodibenzo-*p*-dioxin	2	-3551.498	-1.729	0.063	-9.047
1,2-dibromo-3,6,7,8-tetrachlorodibenzo-*p*-dioxin	2	-3551.588	0.093	-0.003	-10.836
1,2-dibromo-3,6,7,9-tetrachlorodibenzo-*p*-dioxin	2	-3551.630	-0.539	0.036	-10.620
1,2-dibromo-3,6,8,9-tetrachlorodibenzo-*p*-dioxin	2	-3551.631	-1.298	0.068	-9.984
1,2-dibromo-3,7,8,9-tetrachlorodibenzo-*p*-dioxin	2	-3551.579	-1.188	0.054	-9.843
1,2-dibromo-4,6,7,8-tetrachlorodibenzo-*p*-dioxin	2	-3551.669	0.731	-0.038	-11.145
1,2-dibromo-4,6,7,9-tetrachlorodibenzo-*p*-dioxin	2	-3551.714	0.097	-0.004	-10.794
1,2-dibromo-4,6,8,9-tetrachlorodibenzo-*p*-dioxin	2	-3551.712	-0.655	0.026	-10.121
1,2-dibromo-4,7,8,9-tetrachlorodibenzo-*p*-dioxin	2	-3551.669	-0.505	0.003	-9.821
1,2-dibromo-6,7,8,9-tetrachlorodibenzo-*p*-dioxin	2	-3551.540	-0.607	0.033	-10.427
1,3-dibromo-2,4,6,7-tetrachlorodibenzo-*p*-dioxin	2	-3551.373	0.768	0.007	-12.440
1,3-dibromo-2,4,6,8-tetrachlorodibenzo-*p*-dioxin	2	-3551.499	-0.140	0.021	-11.026
1,3-dibromo-2,4,6,9-tetrachlorodibenzo-*p*-dioxin	2	-3551.414	-0.988	0.070	-10.676
1,3-dibromo-2,4,7,8-tetrachlorodibenzo-*p*-dioxin	2	-3551.449	0.018	0.002	-10.847
1,3-dibromo-2,4,7,9-tetrachlorodibenzo-*p*-dioxin	2	-3551.503	-0.655	0.038	-10.472
1,3-dibromo-2,6,7,8-tetrachlorodibenzo-*p*-dioxin	2	-3551.491	0.073	-0.003	-10.816
1,3-dibromo-2,6,7,9-tetrachlorodibenzo-*p*-dioxin	2	-3551.536	-0.561	0.037	-10.621
1,3-dibromo-2,6,8,9-tetrachlorodibenzo-*p*-dioxin	2	-3551.535	-1.317	0.070	-10.017
1,3-dibromo-2,7,8,9-tetrachlorodibenzo-*p*-dioxin	2	-3551.485	-1.209	0.054	-9.820
1,3-dibromo-4,6,7,8-tetrachlorodibenzo-*p*-dioxin	2	-3551.584	1.448	-0.075	-11.585
1,3-dibromo-4,6,7,9-tetrachlorodibenzo-*p*-dioxin	2	-3551.629	0.805	-0.033	-11.425
1,3-dibromo-4,6,8,9-tetrachlorodibenzo-*p*-dioxin	2	-3551.628	0.056	0.005	-10.965
1,3-dibromo-4,7,8,9-tetrachlorodibenzo-*p*-dioxin	2	-3551.585	0.209	-0.016	-10.723
1,3-dibromo-6,7,8,9-tetrachlorodibenzo-*p*-dioxin	2	-3551.600	0.266	-0.021	-10.701
1,4-dibromo-2,3,6,7-tetrachlorodibenzo-*p*-dioxin	2	-3551.418	0.697	0.012	-12.425
1,4-dibromo-2,3,6,8-tetrachlorodibenzo-*p*-dioxin	2	-3551.547	-0.218	0.027	-11.022
1,4-dibromo-2,3,6,9-tetrachlorodibenzo-*p*-dioxin	2	-3551.459	-1.068	0.076	-10.669
1,4-dibromo-2,3,7,8-tetrachlorodibenzo-*p*-dioxin	2	-3551.495	-0.051	0.005	-10.783
1,4-dibromo-2,6,7,8-tetrachlorodibenzo-*p*-dioxin	2	-3551.584	0.622	-0.037	-10.976
1,4-dibromo-2,6,7,9-tetrachlorodibenzo-*p*-dioxin	2	-3551.629	-0.014	0.001	-10.725
1,4-dibromo-2,6,8,9-tetrachlorodibenzo-*p*-dioxin	2	-3551.628	-0.771	0.030	-10.016

1,4-dibromo-2,7,8,9-tetrachlorodibenzo-*p*-dioxin	2	-3551.585	-0.615	0.002	-9.599
1,4-dibromo-6,7,8,9-tetrachlorodibenzo-*p*-dioxin	2	-3551.528	1.037	-0.085	-10.542
1,6-dibromo-2,3,4,7-tetrachlorodibenzo-*p*-dioxin	2	-3551.428	0.705	0.007	-12.308
1,6-dibromo-2,3,4,8-tetrachlorodibenzo-*p*-dioxin	2	-3551.578	-0.196	0.024	-10.981
1,6-dibromo-2,3,4,9-tetrachlorodibenzo-*p*-dioxin	2	-3551.499	-1.030	0.076	-10.734
1,6-dibromo-2,3,7,8-tetrachlorodibenzo-*p*-dioxin	2	-3551.507	0.000	0.000	-10.750
1,6-dibromo-2,3,7,9-tetrachlorodibenzo-*p*-dioxin	2	-3551.558	-0.615	0.041	-10.616
1,6-dibromo-2,3,8,9-tetrachlorodibenzo-*p*-dioxin	2	-3551.582	-1.365	0.079	-10.149
1,6-dibromo-2,4,7,9-tetrachlorodibenzo-*p*-dioxin	2	-3551.611	0.000	0.005	-10.860
1,6-dibromo-2,4,8,9-tetrachlorodibenzo-*p*-dioxin	2	-3551.633	-0.741	0.037	-10.255
1,6-dibromo-3,4,8,9-tetrachlorodibenzo-*p*-dioxin	2	-3551.658	0.001	0.010	-10.983
1,7-dibromo-2,3,4,6-tetrachlorodibenzo-*p*-dioxin	2	-3551.426	0.728	0.003	-12.249
1,7-dibromo-2,3,4,8-tetrachlorodibenzo-*p*-dioxin	2	-3551.497	-0.009	0.002	-10.786
1,7-dibromo-2,3,4,9-tetrachlorodibenzo-*p*-dioxin	2	-3551.571	-0.699	0.044	-10.530
1,7-dibromo-2,3,6,8-tetrachlorodibenzo-*p*-dioxin	2	-3551.473	0.039	-0.001	-10.805
1,7-dibromo-2,3,6,9-tetrachlorodibenzo-*p*-dioxin	2	-3551.550	-0.602	0.039	-10.591
1,7-dibromo-2,3,8,9-tetrachlorodibenzo-*p*-dioxin	2	-3551.494	-1.235	0.059	-9.898
1,7-dibromo-2,4,6,8-tetrachlorodibenzo-*p*-dioxin	2	-3551.523	0.654	-0.039	-10.998
1,7-dibromo-2,4,6,9-tetrachlorodibenzo-*p*-dioxin	2	-3551.601	0.015	0.001	-10.787
1,7-dibromo-2,4,8,9-tetrachlorodibenzo-*p*-dioxin	2	-3551.551	-0.573	0.009	-9.868
1,7-dibromo-2,6,8,9-tetrachlorodibenzo-*p*-dioxin	2	-3551.387	-0.675	0.034	-10.351
1,7-dibromo-3,4,6,8-tetrachlorodibenzo-*p*-dioxin	2	-3551.549	1.403	-0.071	-11.609
1,7-dibromo-3,4,6,9-tetrachlorodibenzo-*p*-dioxin	2	-3551.626	0.755	-0.032	-11.355
1,7-dibromo-3,4,8,9-tetrachlorodibenzo-*p*-dioxin	2	-3551.576	0.172	-0.022	-10.498
1,7-dibromo-3,6,8,9-tetrachlorodibenzo-*p*-dioxin	2	-3551.539	0.221	-0.021	-10.625
1,7-dibromo-4,6,8,9-tetrachlorodibenzo-*p*-dioxin	2	-3551.458	1.052	-0.086	-10.558
1,8-dibromo-2,3,4,6-tetrachlorodibenzo-*p*-dioxin	2	-3551.571	-0.112	0.020	-11.041
1,8-dibromo-2,3,4,7-tetrachlorodibenzo-*p*-dioxin	2	-3551.497	0.054	0.002	-10.908
1,8-dibromo-2,3,4,9-tetrachlorodibenzo-*p*-dioxin	2	-3551.426	-1.729	0.073	-9.320
1,8-dibromo-2,3,6,7-tetrachlorodibenzo-*p*-dioxin	2	-3551.500	0.115	-0.005	-10.843
1,8-dibromo-2,3,6,9-tetrachlorodibenzo-*p*-dioxin	2	-3551.549	-1.285	0.070	-10.077
1,8-dibromo-2,3,7,9-tetrachlorodibenzo-*p*-dioxin	2	-3551.466	-1.168	0.055	-9.929
1,8-dibromo-2,4,6,7-tetrachlorodibenzo-*p*-dioxin	2	-3551.552	0.732	-0.039	-11.143
1,8-dibromo-2,4,6,9-tetrachlorodibenzo-*p*-dioxin	2	-3551.601	-0.661	0.038	-10.442
1,8-dibromo-2,4,7,9-tetrachlorodibenzo-*p*-dioxin	2	-3551.526	-0.507	0.007	-9.948
1,8-dibromo-2,6,7,9-tetrachlorodibenzo-*p*-dioxin	2	-3551.388	-0.600	0.037	-10.574
1,8-dibromo-3,4,6,7-tetrachlorodibenzo-*p*-dioxin	2	-3551.577	1.474	-0.074	-11.663
1,8-dibromo-3,4,6,9-tetrachlorodibenzo-*p*-dioxin	2	-3551.626	0.077	0.007	-11.058
1,8-dibromo-3,4,7,9-tetrachlorodibenzo-*p*-dioxin	2	-3551.548	0.235	-0.016	-10.780
1,8-dibromo-3,6,7,9-tetrachlorodibenzo-*p*-dioxin	2	-3551.537	0.293	-0.024	-10.687
1,8-dibromo-4,6,7,9-tetrachlorodibenzo-*p*-dioxin	2	-3551.459	1.127	-0.074	-11.015
1,9-dibromo-2,3,4,6-tetrachlorodibenzo-*p*-dioxin	2	-3551.499	-0.918	0.064	-10.639
1,9-dibromo-2,3,4,7-tetrachlorodibenzo-*p*-dioxin	2	-3551.580	-0.559	0.034	-10.539
1,9-dibromo-2,3,4,8-tetrachlorodibenzo-*p*-dioxin	2	-3551.428	-1.657	0.070	-9.381

1,9-dibromo-2,3,6,7-tetrachlorodibenzo-*p*-dioxin	2	-3551.582	-0.470	0.031	-10.632
1,9-dibromo-2,3,6,8-tetrachlorodibenzo-*p*-dioxin	2	-3551.558	-1.237	0.070	-10.168
1,9-dibromo-2,3,7,8-tetrachlorodibenzo-*p*-dioxin	2	-3551.500	-1.107	0.056	-10.066
1,9-dibromo-2,4,6,7-tetrachlorodibenzo-*p*-dioxin	2	-3551.635	0.149	-0.009	-10.780
1,9-dibromo-2,4,6,8-tetrachlorodibenzo-*p*-dioxin	2	-3551.608	-0.609	0.029	-10.307
1,9-dibromo-3,4,6,7-tetrachlorodibenzo-*p*-dioxin	2	-3551.658	0.891	-0.042	-11.352
2,3-dibromo-1,4,6,7-tetrachlorodibenzo-*p*-dioxin	2	-3551.464	0.726	-0.001	-12.134
2,3-dibromo-1,4,6,8-tetrachlorodibenzo-*p*-dioxin	2	-3551.592	-0.179	0.020	-10.907
2,3-dibromo-1,4,6,9-tetrachlorodibenzo-*p*-dioxin	2	-3551.505	-1.025	0.065	-10.457
2,3-dibromo-1,4,7,8-tetrachlorodibenzo-*p*-dioxin	2	-3551.540	-0.015	0.001	-10.740
2,3-dibromo-1,6,7,8-tetrachlorodibenzo-*p*-dioxin	2	-3551.587	0.026	-0.001	-10.759
2,3-dibromo-1,6,7,9-tetrachlorodibenzo-*p*-dioxin	2	-3551.631	-0.608	0.037	-10.512
2,3-dibromo-1,6,8,9-tetrachlorodibenzo-*p*-dioxin	2	-3551.632	-1.363	0.073	-9.988
2,3-dibromo-1,7,8,9-tetrachlorodibenzo-*p*-dioxin	2	-3551.580	-1.247	0.057	-9.807
2,3-dibromo-6,7,8,9-tetrachlorodibenzo-*p*-dioxin	2	-3551.611	0.063	-0.003	-10.774
2,7-dibromo-1,3,4,6-tetrachlorodibenzo-*p*-dioxin	2	-3551.386	0.702	0.001	-12.155
2,7-dibromo-1,3,4,8-tetrachlorodibenzo-*p*-dioxin	2	-3551.458	-0.027	0.002	-10.759
2,7-dibromo-1,3,4,9-tetrachlorodibenzo-*p*-dioxin	2	-3551.530	-0.716	0.042	-10.452
2,7-dibromo-1,3,6,8-tetrachlorodibenzo-*p*-dioxin	2	-3551.442	0.000	0.001	-10.788
2,7-dibromo-1,3,6,9-tetrachlorodibenzo-*p*-dioxin	2	-3551.517	-0.639	0.040	-10.552
2,7-dibromo-1,3,8,9-tetrachlorodibenzo-*p*-dioxin	2	-3551.462	-1.264	0.063	-9.952
2,7-dibromo-1,4,6,9-tetrachlorodibenzo-*p*-dioxin	2	-3551.594	0.000	0.002	-10.785
2,7-dibromo-1,4,8,9-tetrachlorodibenzo-*p*-dioxin	2	-3551.544	-0.579	0.013	-9.961
2,7-dibromo-3,4,8,9-tetrachlorodibenzo-*p*-dioxin	2	-3551.495	0.000	0.001	-10.778
2,8-dibromo-1,3,4,6-tetrachlorodibenzo-*p*-dioxin	2	-3551.532	-0.135	0.018	-10.952
2,8-dibromo-1,3,4,7-tetrachlorodibenzo-*p*-dioxin	2	-3551.456	0.036	0.000	-10.829
2,8-dibromo-1,3,4,9-tetrachlorodibenzo-*p*-dioxin	2	-3551.385	-1.742	0.068	-9.172
2,8-dibromo-1,3,6,7-tetrachlorodibenzo-*p*-dioxin	2	-3551.467	0.077	-0.003	-10.828
2,8-dibromo-1,3,6,9-tetrachlorodibenzo-*p*-dioxin	2	-3551.516	-1.320	0.073	-10.093
2,8-dibromo-1,3,7,9-tetrachlorodibenzo-*p*-dioxin	2	-3551.431	-1.197	0.056	-9.905
2,8-dibromo-1,4,6,7-tetrachlorodibenzo-*p*-dioxin	2	-3551.545	0.718	-0.039	-11.117
2,8-dibromo-1,4,6,9-tetrachlorodibenzo-*p*-dioxin	2	-3551.594	-0.673	0.033	-10.290
2,8-dibromo-3,4,6,7-tetrachlorodibenzo-*p*-dioxin	2	-3551.489	1.340	-0.055	-11.914
1,2,3-tribromo-4,6,7-trichlorodibenzo-*p*-dioxin	2	-3524.991	0.766	0.012	-17.527
1,2,3-tribromo-4,6,8-trichlorodibenzo-*p*-dioxin	2	-3525.118	-0.137	0.019	-15.940
1,2,3-tribromo-4,6,9-trichlorodibenzo-*p*-dioxin	2	-3525.031	-0.982	0.066	-15.545
1,2,3-tribromo-4,7,8-trichlorodibenzo-*p*-dioxin	2	-3525.066	0.022	0.002	-15.816
1,2,3-tribromo-4,7,9-trichlorodibenzo-*p*-dioxin	2	-3525.119	-0.648	0.038	-15.446
1,2,3-tribromo-4,8,9-trichlorodibenzo-*p*-dioxin	2	-3524.990	-1.737	0.063	-14.016
1,2,3-tribromo-6,7,8-trichlorodibenzo-*p*-dioxin	2	-3525.110	0.073	-0.003	-15.776
1,2,3-tribromo-6,7,9-trichlorodibenzo-*p*-dioxin	2	-3525.154	-0.558	0.035	-15.536
1,2,3-tribromo-6,8,9-trichlorodibenzo-*p*-dioxin	2	-3525.154	-1.312	0.068	-14.936
1,2,3-tribromo-7,8,9-trichlorodibenzo-*p*-dioxin	2	-3525.103	-1.202	0.051	-14.716
1,2,4-tribromo-3,6,7-trichlorodibenzo-*p*-dioxin	2	-3524.893	0.697	0.014	-17.464

1,2,4-tribromo-3,6,8-trichlorodibenzo-*p*-dioxin	2	-3525.023	-0.213	0.025	-15.967
1,2,4-tribromo-3,6,9-trichlorodibenzo-*p*-dioxin	2	-3524.934	-1.062	0.069	-15.486
1,2,4-tribromo-3,7,8-trichlorodibenzo-*p*-dioxin	2	-3524.969	-0.046	0.004	-15.755
1,2,4-tribromo-3,7,9-trichlorodibenzo-*p*-dioxin	2	-3525.020	-0.718	0.039	-15.356
1,2,4-tribromo-3,8,9-trichlorodibenzo-*p*-dioxin	2	-3524.893	-1.816	0.068	-14.011
1,2,4-tribromo-6,7,8-trichlorodibenzo-*p*-dioxin	2	-3525.107	0.641	-0.037	-15.992
1,2,4-tribromo-6,7,9-trichlorodibenzo-*p*-dioxin	2	-3525.151	0.006	0.002	-15.769
1,2,4-tribromo-6,8,9-trichlorodibenzo-*p*-dioxin	2	-3525.150	-0.749	0.026	-14.934
1,2,4-tribromo-7,8,9-trichlorodibenzo-*p*-dioxin	2	-3525.106	-0.590	0.001	-14.600
1,2,6-tribromo-3,4,7-trichlorodibenzo-*p*-dioxin	2	-3524.912	0.703	0.000	-17.109
1,2,6-tribromo-3,4,8-trichlorodibenzo-*p*-dioxin	2	-3525.063	-0.191	0.025	-16.002
1,2,6-tribromo-3,4,9-trichlorodibenzo-*p*-dioxin	2	-3524.983	-1.023	0.068	-15.526
1,2,6-tribromo-3,7,8-trichlorodibenzo-*p*-dioxin	2	-3524.995	-0.001	0.001	-15.759
1,2,6-tribromo-3,7,9-trichlorodibenzo-*p*-dioxin	2	-3525.045	-0.613	0.037	-15.502
1,2,6-tribromo-3,8,9-trichlorodibenzo-*p*-dioxin	2	-3525.070	-1.360	0.075	-15.040
1,2,6-tribromo-4,7,8-trichlorodibenzo-*p*-dioxin	2	-3525.077	0.634	-0.038	-15.958
1,2,6-tribromo-4,7,9-trichlorodibenzo-*p*-dioxin	2	-3525.129	0.020	-0.001	-15.722
1,2,6-tribromo-4,8,9-trichlorodibenzo-*p*-dioxin	2	-3525.152	-0.718	0.033	-15.175
1,2,6-tribromo-7,8,9-trichlorodibenzo-*p*-dioxin	2	-3524.950	-0.688	0.042	-15.505
1,2,7-tribromo-3,4,6-trichlorodibenzo-*p*-dioxin	2	-3524.911	0.731	0.005	-17.293
1,2,7-tribromo-3,4,8-trichlorodibenzo-*p*-dioxin	2	-3524.981	-0.001	0.004	-15.839
1,2,7-tribromo-3,4,9-trichlorodibenzo-*p*-dioxin	2	-3525.054	-0.689	0.040	-15.431
1,2,7-tribromo-3,6,8-trichlorodibenzo-*p*-dioxin	2	-3524.963	0.040	-0.002	-15.766
1,2,7-tribromo-3,6,9-trichlorodibenzo-*p*-dioxin	2	-3525.038	-0.597	0.037	-15.534
1,2,7-tribromo-3,8,9-trichlorodibenzo-*p*-dioxin	2	-3524.982	-1.227	0.057	-14.847
1,2,7-tribromo-4,6,8-trichlorodibenzo-*p*-dioxin	2	-3525.043	0.675	-0.039	-16.017
1,2,7-tribromo-4,6,9-trichlorodibenzo-*p*-dioxin	2	-3525.120	0.037	0.000	-15.783
1,2,7-tribromo-4,8,9-trichlorodibenzo-*p*-dioxin	2	-3525.070	-0.547	0.007	-14.846
1,2,7-tribromo-6,8,9-trichlorodibenzo-*p*-dioxin	2	-3524.909	-0.660	0.033	-15.333
1,2,8-tribromo-3,4,6-trichlorodibenzo-*p*-dioxin	2	-3525.056	-0.105	0.019	-16.014
1,2,8-tribromo-3,4,7-trichlorodibenzo-*p*-dioxin	2	-3524.981	0.061	0.001	-15.881
1,2,8-tribromo-3,4,9-trichlorodibenzo-*p*-dioxin	2	-3524.909	-1.715	0.068	-14.203
1,2,8-tribromo-3,6,7-trichlorodibenzo-*p*-dioxin	2	-3524.989	0.116	-0.003	-15.882
1,2,8-tribromo-3,6,9-trichlorodibenzo-*p*-dioxin	2	-3525.037	-1.278	0.068	-15.023
1,2,8-tribromo-3,7,9-trichlorodibenzo-*p*-dioxin	2	-3524.953	-1.160	0.052	-14.852
1,2,8-tribromo-4,6,7-trichlorodibenzo-*p*-dioxin	2	-3525.071	0.752	-0.038	-16.187
1,2,8-tribromo-4,6,9-trichlorodibenzo-*p*-dioxin	2	-3525.120	-0.636	0.027	-15.184
1,2,8-tribromo-4,7,9-trichlorodibenzo-*p*-dioxin	2	-3525.043	-0.480	0.007	-14.980
1,2,8-tribromo-6,7,9-trichlorodibenzo-*p*-dioxin	2	-3524.909	-0.585	0.032	-15.452
1,2,9-tribromo-3,4,6-trichlorodibenzo-*p*-dioxin	2	-3524.983	-0.906	0.057	-15.467
1,2,9-tribromo-3,4,7-trichlorodibenzo-*p*-dioxin	2	-3525.062	-0.547	0.032	-15.496
1,2,9-tribromo-3,4,8-trichlorodibenzo-*p*-dioxin	2	-3524.912	-1.640	0.067	-14.322
1,2,9-tribromo-3,6,7-trichlorodibenzo-*p*-dioxin	2	-3525.070	-0.464	0.030	-15.603
1,2,9-tribromo-3,6,8-trichlorodibenzo-*p*-dioxin	2	-3525.047	-1.228	0.066	-15.066

1,2,9-tribromo-3,7,8-trichlorodibenzo-*p*-dioxin	2	-3524.988	-1.097	0.054	-15.019
1,2,9-tribromo-4,6,7-trichlorodibenzo-*p*-dioxin	2	-3525.153	0.173	-0.006	-15.884
1,2,9-tribromo-4,6,8-trichlorodibenzo-*p*-dioxin	2	-3525.127	-0.583	0.025	-15.233
1,2,9-tribromo-4,7,8-trichlorodibenzo-*p*-dioxin	2	-3525.076	-0.413	0.008	-15.129
1,2,9-tribromo-6,7,8-trichlorodibenzo-*p*-dioxin	2	-3524.950	-0.532	0.033	-15.572
1,3,6-tribromo-2,4,7-trichlorodibenzo-*p*-dioxin	2	-3524.787	0.688	0.004	-17.207
1,3,6-tribromo-2,4,8-trichlorodibenzo-*p*-dioxin	2	-3524.936	-0.208	0.023	-15.941
1,3,6-tribromo-2,4,9-trichlorodibenzo-*p*-dioxin	2	-3524.858	-1.041	0.075	-15.697
1,3,6-tribromo-2,7,8-trichlorodibenzo-*p*-dioxin	2	-3524.899	-0.021	0.001	-15.738
1,3,6-tribromo-2,7,9-trichlorodibenzo-*p*-dioxin	2	-3524.952	-0.634	0.041	-15.583
1,3,6-tribromo-2,8,9-trichlorodibenzo-*p*-dioxin	2	-3524.975	-1.378	0.078	-15.102
1,3,6-tribromo-4,7,8-trichlorodibenzo-*p*-dioxin	2	-3524.992	1.353	-0.073	-16.454
1,3,6-tribromo-4,7,9-trichlorodibenzo-*p*-dioxin	2	-3525.044	0.731	-0.027	-16.437
1,3,6-tribromo-4,8,9-trichlorodibenzo-*p*-dioxin	2	-3525.068	-0.006	0.009	-15.943
1,3,6-tribromo-7,8,9-trichlorodibenzo-*p*-dioxin	2	-3525.010	0.185	-0.019	-15.596
1,3,7-tribromo-2,4,6-trichlorodibenzo-*p*-dioxin	2	-3524.784	0.713	0.005	-17.282
1,3,7-tribromo-2,4,8-trichlorodibenzo-*p*-dioxin	2	-3524.854	-0.019	0.002	-15.777
1,3,7-tribromo-2,4,9-trichlorodibenzo-*p*-dioxin	2	-3524.930	-0.708	0.042	-15.470
1,3,7-tribromo-2,6,8-trichlorodibenzo-*p*-dioxin	2	-3524.865	0.020	-0.001	-15.772
1,3,7-tribromo-2,6,9-trichlorodibenzo-*p*-dioxin	2	-3524.942	-0.619	0.043	-15.665
1,3,7-tribromo-2,8,9-trichlorodibenzo-*p*-dioxin	2	-3524.887	-1.247	0.059	-14.878
1,3,7-tribromo-4,6,8-trichlorodibenzo-*p*-dioxin	2	-3524.958	1.391	-0.071	-16.586
1,3,7-tribromo-4,6,9-trichlorodibenzo-*p*-dioxin	2	-3525.035	0.745	-0.029	-16.414
1,3,7-tribromo-4,8,9-trichlorodibenzo-*p*-dioxin	2	-3524.986	0.166	-0.020	-15.538
1,3,7-tribromo-6,8,9-trichlorodibenzo-*p*-dioxin	2	-3524.970	0.210	-0.019	-15.652
1,3,8-tribromo-2,4,6-trichlorodibenzo-*p*-dioxin	2	-3524.928	-0.122	0.030	-16.291
1,3,8-tribromo-2,4,7-trichlorodibenzo-*p*-dioxin	2	-3524.856	0.045	0.000	-15.848
1,3,8-tribromo-2,4,9-trichlorodibenzo-*p*-dioxin	2	-3524.785	-1.732	0.071	-14.272
1,3,8-tribromo-2,6,9-trichlorodibenzo-*p*-dioxin	2	-3524.942	-1.297	0.070	-15.057
1,3,8-tribromo-2,7,9-trichlorodibenzo-*p*-dioxin	2	-3524.860	-1.182	0.053	-14.853
1,3,8-tribromo-4,6,7-trichlorodibenzo-*p*-dioxin	2	-3524.986	1.464	-0.064	-16.903
1,3,8-tribromo-4,6,9-trichlorodibenzo-*p*-dioxin	2	-3525.035	0.071	0.006	-16.020
1,3,8-tribromo-4,7,9-trichlorodibenzo-*p*-dioxin	2	-3524.959	0.230	-0.017	-15.745
1,3,8-tribromo-6,7,9-trichlorodibenzo-*p*-dioxin	2	-3524.969	0.282	-0.023	-15.687
1,3,9-tribromo-2,4,6-trichlorodibenzo-*p*-dioxin	2	-3524.857	-0.929	0.064	-15.628
1,3,9-tribromo-2,4,7-trichlorodibenzo-*p*-dioxin	2	-3524.939	-0.568	0.035	-15.557
1,3,9-tribromo-2,4,8-trichlorodibenzo-*p*-dioxin	2	-3524.786	-1.661	0.071	-14.409
1,3,9-tribromo-2,6,7-trichlorodibenzo-*p*-dioxin	2	-3524.975	-0.488	0.032	-15.627
1,3,9-tribromo-2,6,8-trichlorodibenzo-*p*-dioxin	2	-3524.950	-1.250	0.070	-15.146
1,3,9-tribromo-2,7,8-trichlorodibenzo-*p*-dioxin	2	-3524.893	-1.122	0.055	-15.014
1,3,9-tribromo-4,6,7-trichlorodibenzo-*p*-dioxin	2	-3525.068	0.881	-0.042	-16.333
1,3,9-tribromo-4,6,8-trichlorodibenzo-*p*-dioxin	2	-3525.043	0.128	-0.004	-15.869
1,3,9-tribromo-4,7,8-trichlorodibenzo-*p*-dioxin	2	-3524.992	0.302	-0.018	-15.851
1,3,9-tribromo-6,7,8-trichlorodibenzo-*p*-dioxin	2	-3525.010	0.340	-0.026	-15.714

1,4,6-tribromo-2,3,7-trichlorodibenzo-*p*-dioxin	2	-3524.833	0.631	0.018	-17.452
1,4,6-tribromo-2,3,8-trichlorodibenzo-*p*-dioxin	2	-3524.984	-0.273	0.028	-15.937
1,4,6-tribromo-2,3,9-trichlorodibenzo-*p*-dioxin	2	-3524.904	-1.108	0.079	-15.663
1,4,6-tribromo-2,7,8-trichlorodibenzo-*p*-dioxin	2	-3524.993	0.539	-0.035	-15.868
1,4,6-tribromo-2,7,9-trichlorodibenzo-*p*-dioxin	2	-3525.046	-0.077	0.006	-15.732
1,4,6-tribromo-2,8,9-trichlorodibenzo-*p*-dioxin	2	-3525.069	-0.821	0.035	-15.044
1,4,6-tribromo-3,7,8-trichlorodibenzo-*p*-dioxin	2	-3524.994	1.297	-0.068	-16.475
1,4,6-tribromo-3,7,9-trichlorodibenzo-*p*-dioxin	2	-3525.044	0.671	-0.029	-16.269
1,4,6-tribromo-3,8,9-trichlorodibenzo-*p*-dioxin	2	-3525.069	-0.074	0.015	-15.967
1,4,6-tribromo-7,8,9-trichlorodibenzo-*p*-dioxin	2	-3524.939	0.966	-0.082	-15.483
1,4,7-tribromo-2,3,6-trichlorodibenzo-*p*-dioxin	2	-3524.830	0.641	0.015	-17.394
1,4,7-tribromo-2,3,8-trichlorodibenzo-*p*-dioxin	2	-3524.901	-0.089	0.006	-15.736
1,4,7-tribromo-2,3,9-trichlorodibenzo-*p*-dioxin	2	-3524.975	-0.780	0.045	-15.400
1,4,7-tribromo-2,6,8-trichlorodibenzo-*p*-dioxin	2	-3524.958	0.564	-0.034	-15.949
1,4,7-tribromo-2,6,9-trichlorodibenzo-*p*-dioxin	2	-3525.036	-0.075	0.005	-15.712
1,4,7-tribromo-2,8,9-trichlorodibenzo-*p*-dioxin	2	-3524.986	-0.657	0.005	-14.597
1,4,7-tribromo-3,6,8-trichlorodibenzo-*p*-dioxin	2	-3524.960	1.320	-0.064	-16.630
1,4,7-tribromo-3,6,9-trichlorodibenzo-*p*-dioxin	2	-3525.035	0.671	-0.024	-16.401
1,4,7-tribromo-3,8,9-trichlorodibenzo-*p*-dioxin	2	-3524.986	0.095	-0.018	-15.453
1,4,7-tribromo-6,8,9-trichlorodibenzo-*p*-dioxin	2	-3524.897	0.976	-0.087	-15.380
1,7,8-tribromo-2,3,4-trichlorodibenzo-*p*-dioxin	2	-3525.020	0.014	0.004	-15.861
1,7,8-tribromo-2,3,6-trichlorodibenzo-*p*-dioxin	2	-3524.994	0.068	-0.002	-15.814
1,7,8-tribromo-2,3,9-trichlorodibenzo-*p*-dioxin	2	-3524.987	-1.208	0.059	-14.934
1,7,8-tribromo-2,4,6-trichlorodibenzo-*p*-dioxin	2	-3525.045	0.682	-0.039	-16.030
1,7,8-tribromo-2,4,9-trichlorodibenzo-*p*-dioxin	2	-3525.046	-0.549	0.007	-14.846
1,7,8-tribromo-2,6,9-trichlorodibenzo-*p*-dioxin	2	-3524.877	-0.643	0.033	-15.372
1,7,8-tribromo-3,4,6-trichlorodibenzo-*p*-dioxin	2	-3525.070	1.426	-0.072	-16.606
1,7,8-tribromo-3,4,9-trichlorodibenzo-*p*-dioxin	2	-3525.070	0.193	-0.019	-15.600
1,7,8-tribromo-3,6,9-trichlorodibenzo-*p*-dioxin	2	-3525.028	0.249	-0.021	-15.665
1,7,8-tribromo-4,6,9-trichlorodibenzo-*p*-dioxin	2	-3524.948	1.079	-0.081	-15.725
2,3,7-tribromo-1,4,6-trichlorodibenzo-*p*-dioxin	2	-3524.875	0.670	0.002	-17.104
2,3,7-tribromo-1,4,8-trichlorodibenzo-*p*-dioxin	2	-3524.946	-0.053	0.002	-15.694
2,3,7-tribromo-1,4,9-trichlorodibenzo-*p*-dioxin	2	-3525.020	-0.740	0.043	-15.417
2,3,7-tribromo-1,6,8-trichlorodibenzo-*p*-dioxin	2	-3524.962	-0.029	0.002	-15.737
2,3,7-tribromo-1,6,9-trichlorodibenzo-*p*-dioxin	2	-3525.038	-0.667	0.042	-15.529
2,3,7-tribromo-1,8,9-trichlorodibenzo-*p*-dioxin	2	-3524.983	-1.287	0.064	-14.912
2,3,7-tribromo-4,6,8-trichlorodibenzo-*p*-dioxin	2	-3524.954	1.238	-0.053	-16.758
2,3,7-tribromo-4,6,9-trichlorodibenzo-*p*-dioxin	2	-3525.038	0.555	-0.016	-16.384
2,3,7-tribromo-4,8,9-trichlorodibenzo-*p*-dioxin	2	-3524.989	-0.019	0.002	-15.751
2,3,7-tribromo-6,8,9-trichlorodibenzo-*p*-dioxin	2	-3524.980	0.005	0.000	-15.747
1,2,3,4-tetrabromo-6,7-dichlorodibenzo-*p*-dioxin	2	-3498.513	0.681	0.012	-22.342
1,2,3,4-tetrabromo-6,8-dichlorodibenzo-*p*-dioxin	2	-3498.641	-0.225	0.026	-20.931
1,2,3,4-tetrabromo-6,9-dichlorodibenzo-*p*-dioxin	2	-3498.553	-1.069	0.069	-20.433
1,2,3,4-tetrabromo-7,8-dichlorodibenzo-*p*-dioxin	2	-3498.589	-0.058	0.005	-20.718

1,2,3,6-tetrabromo-4,7-dichlorodibenzo-*p*-dioxin	2	-3498.405	0.685	0.008	-22.266
1,2,3,6-tetrabromo-4,8-dichlorodibenzo-*p*-dioxin	2	-3498.554	-0.207	0.022	-20.878
1,2,3,6-tetrabromo-4,9-dichlorodibenzo-*p*-dioxin	2	-3498.475	-1.038	0.069	-20.508
1,2,3,6-tetrabromo-7,8-dichlorodibenzo-*p*-dioxin	2	-3498.518	-0.021	0.000	-20.673
1,2,3,6-tetrabromo-7,9-dichlorodibenzo-*p*-dioxin	2	-3498.569	-0.633	0.039	-20.494
1,2,3,6-tetrabromo-8,9-dichlorodibenzo-*p*-dioxin	2	-3498.593	-1.374	0.076	-20.018
1,2,3,7-tetrabromo-4,6-dichlorodibenzo-*p*-dioxin	2	-3498.402	0.711	0.004	-22.213
1,2,3,7-tetrabromo-4,8-dichlorodibenzo-*p*-dioxin	2	-3498.472	-0.015	0.002	-20.745
1,2,3,7-tetrabromo-4,9-dichlorodibenzo-*p*-dioxin	2	-3498.547	-0.701	0.041	-20.418
1,2,3,7-tetrabromo-6,8-dichlorodibenzo-*p*-dioxin	2	-3498.485	0.020	-0.001	-20.732
1,2,3,7-tetrabromo-6,9-dichlorodibenzo-*p*-dioxin	2	-3498.561	-0.616	0.039	-20.528
1,2,3,7-tetrabromo-8,9-dichlorodibenzo-*p*-dioxin	2	-3498.505	-1.241	0.058	-19.824
1,2,3,8-tetrabromo-4,6-dichlorodibenzo-*p*-dioxin	2	-3498.546	-0.121	0.020	-20.994
1,2,3,8-tetrabromo-4,7-dichlorodibenzo-*p*-dioxin	2	-3498.473	0.049	0.000	-20.817
1,2,3,8-tetrabromo-4,9-dichlorodibenzo-*p*-dioxin	2	-3498.401	-1.723	0.071	-19.250
1,2,3,8-tetrabromo-6,7-dichlorodibenzo-*p*-dioxin	2	-3498.512	0.096	-0.004	-20.796
1,2,3,8-tetrabromo-6,9-dichlorodibenzo-*p*-dioxin	2	-3498.560	-1.292	0.067	-19.949
1,2,3,8-tetrabromo-7,9-dichlorodibenzo-*p*-dioxin	2	-3498.477	-1.175	0.051	-19.775
1,2,3,9-tetrabromo-4,6-dichlorodibenzo-*p*-dioxin	2	-3498.475	-0.921	0.059	-20.474
1,2,3,9-tetrabromo-4,7-dichlorodibenzo-*p*-dioxin	2	-3498.555	-0.559	0.032	-20.457
1,2,3,9-tetrabromo-4,8-dichlorodibenzo-*p*-dioxin	2	-3498.403	-1.649	0.066	-19.263
1,2,3,9-tetrabromo-6,7-dichlorodibenzo-*p*-dioxin	2	-3498.593	-0.483	0.030	-20.545
1,2,3,9-tetrabromo-6,8-dichlorodibenzo-*p*-dioxin	2	-3498.569	-1.243	0.067	-20.042
1,2,3,9-tetrabromo-7,8-dichlorodibenzo-*p*-dioxin	2	-3498.511	-1.112	0.052	-19.917
1,2,4,6-tetrabromo-3,7-dichlorodibenzo-*p*-dioxin	2	-3498.308	0.628	0.010	-22.226
1,2,4,6-tetrabromo-3,8-dichlorodibenzo-*p*-dioxin	2	-3498.459	-0.271	0.026	-20.876
1,2,4,6-tetrabromo-3,9-dichlorodibenzo-*p*-dioxin	2	-3498.378	-1.103	0.075	-20.556
1,2,4,6-tetrabromo-7,8-dichlorodibenzo-*p*-dioxin	2	-3498.516	0.556	-0.036	-20.853
1,2,4,6-tetrabromo-7,9-dichlorodibenzo-*p*-dioxin	2	-3498.567	-0.058	0.003	-20.670
1,2,4,6-tetrabromo-8,9-dichlorodibenzo-*p*-dioxin	2	-3498.590	-0.800	0.032	-19.985
1,2,4,7-tetrabromo-3,6-dichlorodibenzo-*p*-dioxin	2	-3498.305	0.641	0.018	-22.459
1,2,4,7-tetrabromo-3,8-dichlorodibenzo-*p*-dioxin	2	-3498.376	-0.084	0.006	-20.734
1,2,4,7-tetrabromo-3,9-dichlorodibenzo-*p*-dioxin	2	-3498.449	-0.772	0.043	-20.352
1,2,4,7-tetrabromo-6,8-dichlorodibenzo-*p*-dioxin	2	-3498.481	0.583	-0.035	-20.938
1,2,4,7-tetrabromo-6,9-dichlorodibenzo-*p*-dioxin	2	-3498.557	-0.054	0.004	-20.706
1,2,4,7-tetrabromo-8,9-dichlorodibenzo-*p*-dioxin	2	-3498.508	-0.633	0.005	-19.622
1,2,4,8-tetrabromo-3,6-dichlorodibenzo-*p*-dioxin	2	-3498.450	-0.196	0.025	-20.997
1,2,4,8-tetrabromo-3,7-dichlorodibenzo-*p*-dioxin	2	-3498.375	-0.018	0.004	-20.809
1,2,4,8-tetrabromo-3,9-dichlorodibenzo-*p*-dioxin	2	-3498.304	-1.800	0.061	-18.860
1,2,4,8-tetrabromo-6,7-dichlorodibenzo-*p*-dioxin	2	-3498.508	0.663	-0.036	-21.062
1,2,4,8-tetrabromo-6,9-dichlorodibenzo-*p*-dioxin	2	-3498.557	-0.729	0.029	-20.051
1,2,4,8-tetrabromo-7,9-dichlorodibenzo-*p*-dioxin	2	-3498.481	-0.564	0.004	-19.735
1,2,4,9-tetrabromo-3,6-dichlorodibenzo-*p*-dioxin	2	-3498.379	-0.999	0.064	-20.471
1,2,4,9-tetrabromo-3,7-dichlorodibenzo-*p*-dioxin	2	-3498.457	-0.628	0.035	-20.420

1,2,4,9-tetrabromo-3,8-dichlorodibenzo-*p*-dioxin	2	-3498.308	-1.727	0.063	-19.052
1,2,4,9-tetrabromo-6,7-dichlorodibenzo-*p*-dioxin	2	-3498.591	0.083	0.000	-20.860
1,2,4,9-tetrabromo-6,8-dichlorodibenzo-*p*-dioxin	2	-3498.566	-0.677	0.026	-20.072
1,2,4,9-tetrabromo-7,8-dichlorodibenzo-*p*-dioxin	2	-3498.515	-0.498	0.003	-19.830
1,2,6,7-tetrabromo-3,4-dichlorodibenzo-*p*-dioxin	2	-3498.435	0.690	0.000	-22.062
1,2,6,7-tetrabromo-3,8-dichlorodibenzo-*p*-dioxin	2	-3498.484	0.000	0.001	-20.746
1,2,6,7-tetrabromo-3,9-dichlorodibenzo-*p*-dioxin	2	-3498.564	-0.633	0.041	-20.546
1,2,6,7-tetrabromo-4,9-dichlorodibenzo-*p*-dioxin	2	-3498.647	0.000	0.001	-20.715
1,2,6,8-tetrabromo-3,4-dichlorodibenzo-*p*-dioxin	2	-3498.495	-0.180	0.021	-20.915
1,2,6,8-tetrabromo-3,7-dichlorodibenzo-*p*-dioxin	2	-3498.387	0.019	-0.001	-20.749
1,2,6,8-tetrabromo-3,9-dichlorodibenzo-*p*-dioxin	2	-3498.479	-1.348	0.076	-20.090
1,2,6,8-tetrabromo-4,7-dichlorodibenzo-*p*-dioxin	2	-3498.470	0.653	-0.036	-21.049
1,2,6,8-tetrabromo-4,9-dichlorodibenzo-*p*-dioxin	2	-3498.562	-0.707	0.037	-20.300
1,2,6,8-tetrabromo-7,9-dichlorodibenzo-*p*-dioxin	2	-3498.309	-0.672	0.043	-20.571
1,2,6,9-tetrabromo-3,4-dichlorodibenzo-*p*-dioxin	2	-3498.422	-0.957	0.062	-20.492
1,2,6,9-tetrabromo-3,7-dichlorodibenzo-*p*-dioxin	2	-3498.481	-0.535	0.032	-20.518
1,2,6,9-tetrabromo-3,8-dichlorodibenzo-*p*-dioxin	2	-3498.482	-1.289	0.075	-20.177
1,2,6,9-tetrabromo-4,7-dichlorodibenzo-*p*-dioxin	2	-3498.564	0.100	-0.003	-20.820
1,2,6,9-tetrabromo-4,8-dichlorodibenzo-*p*-dioxin	2	-3498.563	-0.646	0.032	-20.288
1,2,6,9-tetrabromo-7,8-dichlorodibenzo-*p*-dioxin	2	-3498.355	-0.613	0.040	-20.598
1,2,7,8-tetrabromo-3,4-dichlorodibenzo-*p*-dioxin	2	-3498.504	0.021	0.003	-20.835
1,2,7,8-tetrabromo-3,6-dichlorodibenzo-*p*-dioxin	2	-3498.483	0.069	-0.003	-20.775
1,2,7,8-tetrabromo-3,9-dichlorodibenzo-*p*-dioxin	2	-3498.475	-1.200	0.057	-19.883
1,2,7,8-tetrabromo-4,6-dichlorodibenzo-*p*-dioxin	2	-3498.564	0.703	-0.040	-21.024
1,2,7,8-tetrabromo-4,9-dichlorodibenzo-*p*-dioxin	2	-3498.564	-0.522	0.008	-19.904
1,2,7,8-tetrabromo-6,9-dichlorodibenzo-*p*-dioxin	2	-3498.399	-0.628	0.035	-20.431
1,2,7,9-tetrabromo-3,4-dichlorodibenzo-*p*-dioxin	2	-3498.494	-0.598	0.037	-20.523
1,2,7,9-tetrabromo-3,6-dichlorodibenzo-*p*-dioxin	2	-3498.481	-0.522	0.032	-20.543
1,2,7,9-tetrabromo-3,8-dichlorodibenzo-*p*-dioxin	2	-3498.381	-1.133	0.059	-20.082
1,2,7,9-tetrabromo-4,6-dichlorodibenzo-*p*-dioxin	2	-3498.562	0.112	-0.004	-20.818
1,2,7,9-tetrabromo-4,8-dichlorodibenzo-*p*-dioxin	2	-3498.468	-0.452	0.010	-20.109
1,2,7,9-tetrabromo-6,8-dichlorodibenzo-*p*-dioxin	2	-3498.309	-0.586	0.035	-20.529
1,2,8,9-tetrabromo-3,4-dichlorodibenzo-*p*-dioxin	2	-3498.434	-1.630	0.066	-19.294
1,2,8,9-tetrabromo-3,6-dichlorodibenzo-*p*-dioxin	2	-3498.564	-1.212	0.066	-20.077
1,2,8,9-tetrabromo-3,7-dichlorodibenzo-*p*-dioxin	2	-3498.475	-1.071	0.053	-20.029
1,2,8,9-tetrabromo-4,6-dichlorodibenzo-*p*-dioxin	2	-3498.646	-0.570	0.025	-20.237
1,3,6,8-tetrabromo-2,4-dichlorodibenzo-*p*-dioxin	2	-3498.369	-0.196	0.023	-20.960
1,3,6,8-tetrabromo-2,7-dichlorodibenzo-*p*-dioxin	2	-3498.292	0.000	0.002	-20.808
1,3,6,8-tetrabromo-2,9-dichlorodibenzo-*p*-dioxin	2	-3498.385	-1.366	0.088	-20.384
1,3,6,8-tetrabromo-4,9-dichlorodibenzo-*p*-dioxin	2	-3498.478	0.001	0.009	-20.957
1,3,6,9-tetrabromo-2,4-dichlorodibenzo-*p*-dioxin	2	-3498.297	-0.978	0.070	-20.683
1,3,6,9-tetrabromo-2,7-dichlorodibenzo-*p*-dioxin	2	-3498.387	-0.559	0.037	-20.619
1,3,6,9-tetrabromo-2,8-dichlorodibenzo-*p*-dioxin	2	-3498.386	-1.310	0.077	-20.207
1,3,6,9-tetrabromo-4,7-dichlorodibenzo-*p*-dioxin	2	-3498.479	0.810	-0.035	-21.377

1,3,6,9-tetrabromo-4,8-dichlorodibenzo-*p*-dioxin	2	-3498.479	0.066	0.003	-20.926
1,3,6,9-tetrabromo-7,8-dichlorodibenzo-*p*-dioxin	2	-3498.415	0.260	-0.020	-20.716
1,3,7,8-tetrabromo-2,4-dichlorodibenzo-*p*-dioxin	2	-3498.379	0.004	0.001	-20.773
1,3,7,8-tetrabromo-2,6-dichlorodibenzo-*p*-dioxin	2	-3498.386	0.049	-0.002	-20.781
1,3,7,8-tetrabromo-2,9-dichlorodibenzo-*p*-dioxin	2	-3498.381	-1.221	0.058	-19.886
1,3,7,8-tetrabromo-4,6-dichlorodibenzo-*p*-dioxin	2	-3498.479	1.414	-0.073	-21.557
1,3,7,8-tetrabromo-4,9-dichlorodibenzo-*p*-dioxin	2	-3498.480	0.187	-0.012	-20.770
1,3,7,8-tetrabromo-6,9-dichlorodibenzo-*p*-dioxin	2	-3498.459	0.238	-0.021	-20.639
1,3,7,9-tetrabromo-2,4-dichlorodibenzo-*p*-dioxin	2	-3498.369	-0.619	0.041	-20.610
1,3,7,9-tetrabromo-2,6-dichlorodibenzo-*p*-dioxin	2	-3498.385	-0.547	0.036	-20.617
1,3,7,9-tetrabromo-2,8-dichlorodibenzo-*p*-dioxin	2	-3498.286	-1.157	0.060	-20.080
1,4,6,9-tetrabromo-2,3-dichlorodibenzo-*p*-dioxin	2	-3498.344	-1.045	0.076	-20.701
1,4,6,9-tetrabromo-2,7-dichlorodibenzo-*p*-dioxin	2	-3498.482	0.000	0.000	-20.720
1,4,6,9-tetrabromo-2,8-dichlorodibenzo-*p*-dioxin	2	-3498.480	-0.751	0.036	-20.204
1,4,7,8-tetrabromo-2,3-dichlorodibenzo-*p*-dioxin	2	-3498.424	-0.065	0.006	-20.761
1,4,7,8-tetrabromo-2,6-dichlorodibenzo-*p*-dioxin	2	-3498.480	0.594	-0.036	-20.934
1,4,7,8-tetrabromo-2,9-dichlorodibenzo-*p*-dioxin	2	-3498.481	-0.632	0.008	-19.707
1,4,7,8-tetrabromo-6,9-dichlorodibenzo-*p*-dioxin	2	-3498.387	1.004	-0.083	-20.523
2,3,7,8-tetrabromo-1,4-dichlorodibenzo-*p*-dioxin	2	-3498.469	-0.031	0.002	-20.715
2,3,7,8-tetrabromo-1,6-dichlorodibenzo-*p*-dioxin	2	-3498.483	0.000	0.001	-20.746
2,3,7,8-tetrabromo-1,9-dichlorodibenzo-*p*-dioxin	2	-3498.476	-1.261	0.064	-19.947
1,2,3,4,6-pentabromo-7-chlorodibenzo-*p*-dioxin	2	-3471.927	0.610	0.009	-27.126
1,2,3,4,6-pentabromo-8-chlorodibenzo-*p*-dioxin	2	-3472.077	-0.283	0.027	-25.840
1,2,3,4,6-pentabromo-9-chlorodibenzo-*p*-dioxin	2	-3471.997	-1.113	0.077	-25.549
1,2,3,4,7-pentabromo-6-chlorodibenzo-*p*-dioxin	2	-3471.924	0.625	0.015	-27.311
1,2,3,4,7-pentabromo-8-chlorodibenzo-*p*-dioxin	2	-3471.995	-0.096	0.004	-25.619
1,2,3,4,7-pentabromo-9-chlorodibenzo-*p*-dioxin	2	-3472.069	-0.781	0.046	-25.372
1,2,3,6,7-pentabromo-4-chlorodibenzo-*p*-dioxin	2	-3471.926	0.670	0.001	-27.034
1,2,3,6,7-pentabromo-8-chlorodibenzo-*p*-dioxin	2	-3472.006	-0.020	0.001	-25.686
1,2,3,6,7-pentabromo-9-chlorodibenzo-*p*-dioxin	2	-3472.088	-0.652	0.042	-25.514
1,2,3,6,8-pentabromo-4-chlorodibenzo-*p*-dioxin	2	-3471.986	-0.196	0.020	-25.843
1,2,3,6,8-pentabromo-7-chlorodibenzo-*p*-dioxin	2	-3471.911	-0.002	0.000	-25.713
1,2,3,6,8-pentabromo-9-chlorodibenzo-*p*-dioxin	2	-3472.003	-1.362	0.076	-25.041
1,2,3,6,9-pentabromo-4-chlorodibenzo-*p*-dioxin	2	-3471.915	-0.973	0.066	-25.550
1,2,3,6,9-pentabromo-7-chlorodibenzo-*p*-dioxin	2	-3472.004	-0.555	0.034	-25.510
1,2,3,6,9-pentabromo-8-chlorodibenzo-*p*-dioxin	2	-3472.005	-1.305	0.074	-25.099
1,2,3,7,8-pentabromo-4-chlorodibenzo-*p*-dioxin	2	-3471.996	0.008	0.002	-25.768
1,2,3,7,8-pentabromo-6-chlorodibenzo-*p*-dioxin	2	-3472.006	0.049	-0.002	-25.742
1,2,3,7,8-pentabromo-9-chlorodibenzo-*p*-dioxin	2	-3471.999	-1.214	0.055	-24.783
1,2,3,7,9-pentabromo-4-chlorodibenzo-*p*-dioxin	2	-3471.986	-0.611	0.035	-25.431
1,2,3,7,9-pentabromo-6-chlorodibenzo-*p*-dioxin	2	-3472.003	-0.543	0.033	-25.507
1,2,3,7,9-pentabromo-8-chlorodibenzo-*p*-dioxin	2	-3471.904	-1.148	0.061	-25.084
1,2,3,8,9-pentabromo-4-chlorodibenzo-*p*-dioxin	2	-3471.926	-1.638	0.072	-24.419
1,2,3,8,9-pentabromo-6-chlorodibenzo-*p*-dioxin	2	-3472.087	-1.227	0.066	-25.027

1,2,3,8,9-pentabromo-7-chlorodibenzo-*p*-dioxin	2	-3471.999	-1.087	0.052	-24.950
1,2,4,6,7-pentabromo-3-chlorodibenzo-*p*-dioxin	2	-3471.830	0.613	0.013	-27.254
1,2,4,6,7-pentabromo-8-chlorodibenzo-*p*-dioxin	2	-3472.003	0.552	-0.034	-25.883
1,2,4,6,7-pentabromo-9-chlorodibenzo-*p*-dioxin	2	-3472.085	-0.081	0.006	-25.683
1,2,4,6,8-pentabromo-3-chlorodibenzo-*p*-dioxin	2	-3471.890	-0.258	0.027	-25.923
1,2,4,6,8-pentabromo-7-chlorodibenzo-*p*-dioxin	2	-3471.908	0.576	-0.034	-25.947
1,2,4,6,8-pentabromo-9-chlorodibenzo-*p*-dioxin	2	-3472.000	-0.789	0.037	-25.136
1,2,4,6,9-pentabromo-3-chlorodibenzo-*p*-dioxin	2	-3471.819	-1.038	0.069	-25.520
1,2,4,6,9-pentabromo-7-chlorodibenzo-*p*-dioxin	2	-3472.003	0.020	0.001	-25.764
1,2,4,6,9-pentabromo-8-chlorodibenzo-*p*-dioxin	2	-3472.002	-0.728	0.031	-25.098
1,2,4,7,8-pentabromo-3-chlorodibenzo-*p*-dioxin	2	-3471.899	-0.060	0.005	-25.733
1,2,4,7,8-pentabromo-6-chlorodibenzo-*p*-dioxin	2	-3472.002	0.612	-0.036	-25.947
1,2,4,7,8-pentabromo-9-chlorodibenzo-*p*-dioxin	2	-3472.002	-0.607	0.007	-24.709
1,2,4,7,9-pentabromo-3-chlorodibenzo-*p*-dioxin	2	-3471.889	-0.680	0.042	-25.497
1,2,4,7,9-pentabromo-6-chlorodibenzo-*p*-dioxin	2	-3472.000	0.021	0.002	-25.792
1,2,4,7,9-pentabromo-8-chlorodibenzo-*p*-dioxin	2	-3471.907	-0.538	0.007	-24.860
1,2,4,8,9-pentabromo-3-chlorodibenzo-*p*-dioxin	2	-3471.829	-1.715	0.062	-24.028
1,2,4,8,9-pentabromo-6-chlorodibenzo-*p*-dioxin	2	-3472.085	-0.663	0.028	-25.130
1,2,4,8,9-pentabromo-7-chlorodibenzo-*p*-dioxin	2	-3472.002	-0.475	0.003	-24.860
1,2,3,4,6,7-hexabromodibenzo-*p*-dioxin	2	-3445.449	0.595	0.011	-32.128
1,2,3,4,6,8-hexabromodibenzo-*p*-dioxin	2	-3445.509	-0.270	0.027	-30.861
1,2,3,4,6,9-hexabromodibenzo-*p*-dioxin	2	-3445.438	-1.049	0.070	-30.485
1,2,3,4,7,8-hexabromodibenzo-*p*-dioxin	2	-3445.518	-0.072	0.007	-30.722
1,2,3,6,7,8-hexabromodibenzo-*p*-dioxin	2	-3445.529	0.000	0.000	-30.677
1,2,3,6,7,9-hexabromodibenzo-*p*-dioxin	2	-3445.526	-0.572	0.036	-30.508
1,2,3,6,8,9-hexabromodibenzo-*p*-dioxin	2	-3445.526	-1.293	0.075	-30.127
1,2,4,6,7,9-hexabromodibenzo-*p*-dioxin	2	-3445.524	0.000	0.001	-30.704
1,2,4,6,8,9-hexabromodibenzo-*p*-dioxin	2	-3445.524	-0.718	0.031	-30.096
1,2,3,4,6,7,8-heptachlorodibenzo-*p*-dioxin	1	-3845.467	0.586	-0.020	-2.132
1-bromo-2,3,4,6,7,8-hexachlorodibenzo-*p*-dioxin	1	-3818.876	0.669	-0.022	-7.240
1-bromo-2,3,4,6,7,9-hexachlorodibenzo-*p*-dioxin	1	-3818.922	0.041	0.011	-6.875
1-bromo-2,3,4,6,8,9-hexachlorodibenzo-*p*-dioxin	1	-3818.921	-0.704	0.039	-6.163
1-bromo-2,3,4,7,8,9-hexachlorodibenzo-*p*-dioxin	1	-3818.876	-0.550	0.026	-6.132
1-bromo-2,3,6,7,8,9-hexachlorodibenzo-*p*-dioxin	1	-3818.874	-0.489	0.026	-6.250
1-bromo-2,4,6,7,8,9-hexachlorodibenzo-*p*-dioxin	1	-3818.926	0.120	-0.015	-6.352
1-bromo-3,4,6,7,8,9-hexachlorodibenzo-*p*-dioxin	1	-3818.951	0.851	-0.052	-6.799
2-bromo-1,3,4,6,7,8-hexachlorodibenzo-*p*-dioxin	1	-3818.836	0.643	-0.024	-7.146
2-bromo-1,3,4,6,7,9-hexachlorodibenzo-*p*-dioxin	1	-3818.880	0.016	0.011	-6.835
2-bromo-1,3,4,6,8,9-hexachlorodibenzo-*p*-dioxin	1	-3818.882	-0.727	0.039	-6.126
2-bromo-1,3,4,7,8,9-hexachlorodibenzo-*p*-dioxin	1	-3818.835	-0.566	0.021	-5.979
2-bromo-1,3,6,7,8,9-hexachlorodibenzo-*p*-dioxin	1	-3818.841	-0.526	0.030	-6.288
2-bromo-1,4,6,7,8,9-hexachlorodibenzo-*p*-dioxin	1	-3818.918	0.107	-0.013	-6.380
2-bromo-3,4,6,7,8,9-hexachlorodibenzo-*p*-dioxin	1	-3818.869	0.672	-0.032	-6.988
1,2-dibromo-3,4,6,7,8-pentachlorodibenzo-*p*-dioxin	1	-3792.361	0.675	-0.022	-12.238

1,2-dibromo-3,4,6,7,9-pentachlorodibenzo-*p*-dioxin	1	-3792.404	0.050	0.011	-11.879
1,2-dibromo-3,4,6,8,9-pentachlorodibenzo-*p*-dioxin	1	-3792.405	-0.692	0.036	-11.095
1,2-dibromo-3,4,7,8,9-pentachlorodibenzo-*p*-dioxin	1	-3792.359	-0.537	0.019	-10.961
1,2-dibromo-3,6,7,8,9-pentachlorodibenzo-*p*-dioxin	1	-3792.362	-0.482	0.024	-11.197
1,2-dibromo-4,6,7,8,9-pentachlorodibenzo-*p*-dioxin	1	-3792.444	0.145	-0.015	-11.380
1,3-dibromo-2,4,6,7,8-pentachlorodibenzo-*p*-dioxin	1	-3792.234	0.655	-0.023	-12.197
1,3-dibromo-2,4,6,7,9-pentachlorodibenzo-*p*-dioxin	1	-3792.280	0.029	0.012	-11.888
1,3-dibromo-2,4,6,8,9-pentachlorodibenzo-*p*-dioxin	1	-3792.278	-0.712	0.041	-11.210
1,3-dibromo-2,4,7,8,9-pentachlorodibenzo-*p*-dioxin	1	-3792.235	-0.557	0.025	-11.102
1,3-dibromo-2,6,7,8,9-pentachlorodibenzo-*p*-dioxin	1	-3792.266	-0.504	0.026	-11.224
1,3-dibromo-4,6,7,8,9-pentachlorodibenzo-*p*-dioxin	1	-3792.360	0.842	-0.042	-12.041
1,4-dibromo-2,3,6,7,8-pentachlorodibenzo-*p*-dioxin	1	-3792.280	0.585	-0.017	-12.209
1,4-dibromo-2,3,6,7,9-pentachlorodibenzo-*p*-dioxin	1	-3792.325	-0.043	0.020	-11.948
1,4-dibromo-2,6,7,8,9-pentachlorodibenzo-*p*-dioxin	1	-3792.361	0.031	-0.016	-11.149
1,6-dibromo-2,3,4,7,8-pentachlorodibenzo-*p*-dioxin	1	-3792.284	0.572	-0.023	-12.027
1,6-dibromo-2,3,4,7,9-pentachlorodibenzo-*p*-dioxin	1	-3792.337	-0.034	0.014	-11.807
1,6-dibromo-2,3,4,8,9-pentachlorodibenzo-*p*-dioxin	1	-3792.360	-0.767	0.045	-11.192
1,7-dibromo-2,3,4,6,8-pentachlorodibenzo-*p*-dioxin	1	-3792.250	0.611	-0.021	-12.161
1,7-dibromo-2,3,4,6,9-pentachlorodibenzo-*p*-dioxin	1	-3792.328	-0.021	0.016	-11.886
1,7-dibromo-2,3,4,8,9-pentachlorodibenzo-*p*-dioxin	1	-3792.277	-0.593	0.023	-10.972
1,7-dibromo-2,3,6,8,9-pentachlorodibenzo-*p*-dioxin	1	-3792.243	-0.545	0.031	-11.279
1,7-dibromo-2,4,6,8,9-pentachlorodibenzo-*p*-dioxin	1	-3792.294	0.061	-0.014	-11.272
1,7-dibromo-3,4,6,8,9-pentachlorodibenzo-*p*-dioxin	1	-3792.320	0.792	-0.049	-11.771
1,8-dibromo-2,3,4,6,7-pentachlorodibenzo-*p*-dioxin	1	-3792.278	0.688	-0.025	-12.201
1,8-dibromo-2,3,4,6,9-pentachlorodibenzo-*p*-dioxin	1	-3792.328	-0.686	0.039	-11.199
1,8-dibromo-2,3,4,7,9-pentachlorodibenzo-*p*-dioxin	1	-3792.251	-0.526	0.024	-11.133
1,8-dibromo-2,3,6,7,9-pentachlorodibenzo-*p*-dioxin	1	-3792.243	-0.469	0.026	-11.296
1,8-dibromo-2,4,6,7,9-pentachlorodibenzo-*p*-dioxin	1	-3792.296	0.138	-0.013	-11.446
1,8-dibromo-3,4,6,7,9-pentachlorodibenzo-*p*-dioxin	1	-3792.319	0.864	-0.044	-12.040
1,9-dibromo-2,3,4,6,7-pentachlorodibenzo-*p*-dioxin	1	-3792.361	0.115	0.005	-11.857
1,9-dibromo-2,3,4,6,8-pentachlorodibenzo-*p*-dioxin	1	-3792.336	-0.635	0.038	-11.269
1,9-dibromo-2,3,4,7,8-pentachlorodibenzo-*p*-dioxin	1	-3792.285	-0.461	0.021	-11.174
2,3-dibromo-1,4,6,7,8-pentachlorodibenzo-*p*-dioxin	1	-3792.325	0.613	-0.022	-12.125
2,3-dibromo-1,4,6,7,9-pentachlorodibenzo-*p*-dioxin	1	-3792.370	-0.013	0.011	-11.764
2,3-dibromo-1,6,7,8,9-pentachlorodibenzo-*p*-dioxin	1	-3792.362	-0.552	0.029	-11.191
2,7-dibromo-1,3,4,6,8-pentachlorodibenzo-*p*-dioxin	1	-3792.211	0.585	-0.021	-12.118
2,7-dibromo-1,3,4,6,9-pentachlorodibenzo-*p*-dioxin	1	-3792.287	-0.046	0.016	-11.845
2,7-dibromo-1,3,4,8,9-pentachlorodibenzo-*p*-dioxin	1	-3792.237	-0.610	0.024	-10.973
2,8-dibromo-1,3,4,6,9-pentachlorodibenzo-*p*-dioxin	1	-3792.287	-0.709	0.038	-11.136
2,8-dibromo-1,3,4,7,9-pentachlorodibenzo-*p*-dioxin	1	-3792.208	-0.544	0.020	-11.002
1,2,3-tribromo-4,6,7,8-tetrachlorodibenzo-*p*-dioxin	1	-3765.851	0.657	-0.022	-17.188
1,2,3-tribromo-4,6,7,9-tetrachlorodibenzo-*p*-dioxin	1	-3765.897	0.033	0.010	-16.805
1,2,3-tribromo-4,6,8,9-tetrachlorodibenzo-*p*-dioxin	1	-3765.895	-0.705	0.037	-16.080
1,2,3-tribromo-4,7,8,9-tetrachlorodibenzo-*p*-dioxin	1	-3765.851	-0.549	0.020	-15.948

1,2,3-tribromo-6,7,8,9-tetrachlorodibenzo-*p*-dioxin	1	-3765.884	-0.500	0.024	-16.141
1,2,4-tribromo-3,6,7,8-tetrachlorodibenzo-*p*-dioxin	1	-3765.755	0.589	-0.014	-17.282
1,2,4-tribromo-3,6,7,9-tetrachlorodibenzo-*p*-dioxin	1	-3765.799	-0.037	0.017	-16.870
1,2,4-tribromo-3,6,8,9-tetrachlorodibenzo-*p*-dioxin	1	-3765.800	-0.784	0.039	-15.997
1,2,4-tribromo-3,7,8,9-tetrachlorodibenzo-*p*-dioxin	1	-3765.754	-0.618	0.018	-15.781
1,2,4-tribromo-6,7,8,9-tetrachlorodibenzo-*p*-dioxin	1	-3765.882	0.055	-0.015	-16.201
1,2,6-tribromo-3,4,7,8-tetrachlorodibenzo-*p*-dioxin	1	-3765.768	0.577	-0.022	-17.049
1,2,6-tribromo-3,4,7,9-tetrachlorodibenzo-*p*-dioxin	1	-3765.819	-0.028	0.015	-16.831
1,2,6-tribromo-3,4,8,9-tetrachlorodibenzo-*p*-dioxin	1	-3765.844	-0.758	0.043	-16.143
1,2,6-tribromo-3,7,8,9-tetrachlorodibenzo-*p*-dioxin	1	-3765.771	-0.567	0.033	-16.266
1,2,6-tribromo-4,7,8,9-tetrachlorodibenzo-*p*-dioxin	1	-3765.855	0.059	-0.010	-16.343
1,2,7-tribromo-3,4,6,8-tetrachlorodibenzo-*p*-dioxin	1	-3765.735	0.618	-0.021	-17.160
1,2,7-tribromo-3,4,6,9-tetrachlorodibenzo-*p*-dioxin	1	-3765.811	-0.011	0.015	-16.866
1,2,7-tribromo-3,4,8,9-tetrachlorodibenzo-*p*-dioxin	1	-3765.761	-0.579	0.022	-15.959
1,2,7-tribromo-3,6,8,9-tetrachlorodibenzo-*p*-dioxin	1	-3765.731	-0.538	0.028	-16.200
1,2,7-tribromo-4,6,8,9-tetrachlorodibenzo-*p*-dioxin	1	-3765.813	0.087	-0.016	-16.250
1,2,8-tribromo-3,4,6,7-tetrachlorodibenzo-*p*-dioxin	1	-3765.762	0.694	-0.023	-17.250
1,2,8-tribromo-3,4,6,9-tetrachlorodibenzo-*p*-dioxin	1	-3765.811	-0.674	0.037	-16.156
1,2,8-tribromo-3,4,7,9-tetrachlorodibenzo-*p*-dioxin	1	-3765.733	-0.514	0.020	-16.038
1,2,8-tribromo-3,6,7,9-tetrachlorodibenzo-*p*-dioxin	1	-3765.730	-0.463	0.025	-16.267
1,2,8-tribromo-4,6,7,9-tetrachlorodibenzo-*p*-dioxin	1	-3765.814	0.163	-0.012	-16.500
1,2,9-tribromo-3,4,6,7-tetrachlorodibenzo-*p*-dioxin	1	-3765.844	0.126	0.003	-16.812
1,2,9-tribromo-3,4,6,8-tetrachlorodibenzo-*p*-dioxin	1	-3765.820	-0.621	0.036	-16.231
1,2,9-tribromo-3,4,7,8-tetrachlorodibenzo-*p*-dioxin	1	-3765.768	-0.445	0.019	-16.139
1,2,9-tribromo-3,6,7,8-tetrachlorodibenzo-*p*-dioxin	1	-3765.772	-0.409	0.024	-16.338
1,2,9-tribromo-4,6,7,8-tetrachlorodibenzo-*p*-dioxin	1	-3765.855	0.219	-0.017	-16.471
1,3,6-tribromo-2,4,7,8-tetrachlorodibenzo-*p*-dioxin	1	-3765.642	0.557	-0.022	-17.034
1,3,6-tribromo-2,4,7,9-tetrachlorodibenzo-*p*-dioxin	1	-3765.696	-0.047	0.022	-16.999
1,3,6-tribromo-2,4,8,9-tetrachlorodibenzo-*p*-dioxin	1	-3765.718	-0.776	0.043	-16.132
1,3,6-tribromo-2,7,8,9-tetrachlorodibenzo-*p*-dioxin	1	-3765.677	-0.588	0.034	-16.269
1,3,6-tribromo-4,7,8,9-tetrachlorodibenzo-*p*-dioxin	1	-3765.770	0.758	-0.041	-16.905
1,3,7-tribromo-2,4,6,8-tetrachlorodibenzo-*p*-dioxin	1	-3765.607	0.597	-0.022	-17.118
1,3,7-tribromo-2,4,6,9-tetrachlorodibenzo-*p*-dioxin	1	-3765.686	-0.033	0.014	-16.821
1,3,7-tribromo-2,4,8,9-tetrachlorodibenzo-*p*-dioxin	1	-3765.636	-0.600	0.023	-15.968
1,3,7-tribromo-2,6,8,9-tetrachlorodibenzo-*p*-dioxin	1	-3765.635	-0.560	0.030	-16.228
1,3,7-tribromo-4,6,8,9-tetrachlorodibenzo-*p*-dioxin	1	-3765.729	0.783	-0.048	-16.779
1,3,8-tribromo-2,4,6,7-tetrachlorodibenzo-*p*-dioxin	1	-3765.636	0.674	-0.025	-17.183
1,3,8-tribromo-2,4,6,9-tetrachlorodibenzo-*p*-dioxin	1	-3765.686	-0.694	0.041	-16.245
1,3,8-tribromo-2,4,7,9-tetrachlorodibenzo-*p*-dioxin	1	-3765.610	-0.534	0.024	-16.127
1,3,8-tribromo-2,6,7,9-tetrachlorodibenzo-*p*-dioxin	1	-3765.636	-0.485	0.026	-16.268
1,3,8-tribromo-4,6,7,9-tetrachlorodibenzo-*p*-dioxin	1	-3765.729	0.856	-0.043	-17.050
1,3,9-tribromo-2,4,6,7-tetrachlorodibenzo-*p*-dioxin	1	-3765.720	0.102	0.004	-16.815
1,3,9-tribromo-2,4,6,8-tetrachlorodibenzo-*p*-dioxin	1	-3765.693	-0.645	0.036	-16.208
1,3,9-tribromo-2,4,7,8-tetrachlorodibenzo-*p*-dioxin	1	-3765.643	-0.470	0.021	-16.166

1,3,9-tribromo-2,6,7,8-tetrachlorodibenzo-*p*-dioxin	1	-3765.677	-0.435	0.024	-16.305
1,3,9-tribromo-4,6,7,8-tetrachlorodibenzo-*p*-dioxin	1	-3765.770	0.917	-0.046	-17.082
1,4,6-tribromo-2,3,7,8-tetrachlorodibenzo-*p*-dioxin	1	-3765.690	0.501	-0.018	-17.021
1,4,6-tribromo-2,3,7,9-tetrachlorodibenzo-*p*-dioxin	1	-3765.742	-0.106	0.021	-16.851
1,4,6-tribromo-2,3,8,9-tetrachlorodibenzo-*p*-dioxin	1	-3765.766	-0.844	0.046	-16.070
1,4,6-tribromo-2,7,8,9-tetrachlorodibenzo-*p*-dioxin	1	-3765.772	-0.042	-0.010	-16.164
1,4,6-tribromo-3,7,8,9-tetrachlorodibenzo-*p*-dioxin	1	-3765.772	0.699	-0.034	-16.972
1,4,7-tribromo-2,3,6,8-tetrachlorodibenzo-*p*-dioxin	1	-3765.655	0.525	-0.015	-17.152
1,4,7-tribromo-2,3,6,9-tetrachlorodibenzo-*p*-dioxin	1	-3765.732	-0.107	0.024	-16.929
1,4,7-tribromo-2,3,8,9-tetrachlorodibenzo-*p*-dioxin	1	-3765.682	-0.673	0.024	-15.845
1,4,7-tribromo-2,6,8,9-tetrachlorodibenzo-*p*-dioxin	1	-3765.729	-0.029	-0.014	-16.093
1,4,7-tribromo-3,6,8,9-tetrachlorodibenzo-*p*-dioxin	1	-3765.730	0.709	-0.045	-16.714
1,7,8-tribromo-2,3,4,6-tetrachlorodibenzo-*p*-dioxin	1	-3765.771	0.638	-0.023	-17.140
1,7,8-tribromo-2,3,4,9-tetrachlorodibenzo-*p*-dioxin	1	-3765.771	-0.569	0.025	-16.055
1,7,8-tribromo-2,3,6,9-tetrachlorodibenzo-*p*-dioxin	1	-3765.732	-0.514	0.028	-16.246
1,7,8-tribromo-2,4,6,9-tetrachlorodibenzo-*p*-dioxin	1	-3765.784	0.090	-0.014	-16.313
1,7,8-tribromo-3,4,6,9-tetrachlorodibenzo-*p*-dioxin	1	-3765.809	0.818	-0.049	-16.806
2,3,7-tribromo-1,4,6,8-tetrachlorodibenzo-*p*-dioxin	1	-3765.699	0.553	-0.020	-17.068
2,3,7-tribromo-1,4,6,9-tetrachlorodibenzo-*p*-dioxin	1	-3765.776	-0.076	0.014	-16.721
2,3,7-tribromo-1,4,8,9-tetrachlorodibenzo-*p*-dioxin	1	-3765.726	-0.634	0.026	-15.963
2,3,7-tribromo-1,6,8,9-tetrachlorodibenzo-*p*-dioxin	1	-3765.731	-0.610	0.034	-16.217
2,3,7-tribromo-4,6,8,9-tetrachlorodibenzo-*p*-dioxin	1	-3765.730	0.587	-0.029	-16.894
1,2,3,4-tetrabromo-6,7,8-trichlorodibenzo-*p*-dioxin	1	-3739.373	0.572	-0.013	-22.236
1,2,3,4-tetrabromo-6,7,9-trichlorodibenzo-*p*-dioxin	1	-3739.418	-0.051	0.017	-21.803
1,2,3,6-tetrabromo-4,7,8-trichlorodibenzo-*p*-dioxin	1	-3739.260	0.558	-0.020	-22.049
1,2,3,6-tetrabromo-4,7,9-trichlorodibenzo-*p*-dioxin	1	-3739.312	-0.045	0.015	-21.783
1,2,3,6-tetrabromo-4,8,9-trichlorodibenzo-*p*-dioxin	1	-3739.335	-0.771	0.042	-21.077
1,2,3,6-tetrabromo-7,8,9-trichlorodibenzo-*p*-dioxin	1	-3739.295	-0.585	0.031	-21.158
1,2,3,7-tetrabromo-4,6,8-trichlorodibenzo-*p*-dioxin	1	-3739.225	0.599	-0.021	-22.109
1,2,3,7-tetrabromo-4,6,9-trichlorodibenzo-*p*-dioxin	1	-3739.302	-0.028	0.014	-21.791
1,2,3,7-tetrabromo-4,8,9-trichlorodibenzo-*p*-dioxin	1	-3739.252	-0.591	0.021	-20.895
1,2,3,7-tetrabromo-6,8,9-trichlorodibenzo-*p*-dioxin	1	-3739.253	-0.556	0.030	-21.196
1,2,3,8-tetrabromo-4,6,7-trichlorodibenzo-*p*-dioxin	1	-3739.253	0.676	-0.024	-22.174
1,2,3,8-tetrabromo-4,6,9-trichlorodibenzo-*p*-dioxin	1	-3739.302	-0.687	0.038	-21.141
1,2,3,8-tetrabromo-4,7,9-trichlorodibenzo-*p*-dioxin	1	-3739.226	-0.525	0.020	-21.001
1,2,3,8-tetrabromo-6,7,9-trichlorodibenzo-*p*-dioxin	1	-3739.253	-0.480	0.024	-21.187
1,2,3,9-tetrabromo-4,6,7-trichlorodibenzo-*p*-dioxin	1	-3739.336	0.108	0.006	-21.840
1,2,3,9-tetrabromo-4,6,8-trichlorodibenzo-*p*-dioxin	1	-3739.311	-0.636	0.035	-21.160
1,2,3,9-tetrabromo-4,7,8-trichlorodibenzo-*p*-dioxin	1	-3739.259	-0.459	0.019	-21.097
1,2,3,9-tetrabromo-6,7,8-trichlorodibenzo-*p*-dioxin	1	-3739.295	-0.428	0.024	-21.280
1,2,4,6-tetrabromo-3,7,8-trichlorodibenzo-*p*-dioxin	1	-3739.164	0.503	-0.016	-22.064
1,2,4,6-tetrabromo-3,7,9-trichlorodibenzo-*p*-dioxin	1	-3739.215	-0.102	0.021	-21.847
1,2,4,6-tetrabromo-3,8,9-trichlorodibenzo-*p*-dioxin	1	-3739.240	-0.837	0.042	-20.967
1,2,4,6-tetrabromo-7,8,9-trichlorodibenzo-*p*-dioxin	1	-3739.293	-0.020	-0.010	-21.185

1,2,4,7-tetrabromo-3,6,8-trichlorodibenzo-*p*-dioxin	1	-3739.130	0.530	-0.013	-22.201
1,2,4,7-tetrabromo-3,6,9-trichlorodibenzo-*p*-dioxin	1	-3739.206	-0.100	0.023	-21.904
1,2,4,7-tetrabromo-3,8,9-trichlorodibenzo-*p*-dioxin	1	-3739.156	-0.662	0.022	-20.802
1,2,4,7-tetrabromo-6,8,9-trichlorodibenzo-*p*-dioxin	1	-3739.251	-0.005	-0.012	-21.170
1,2,4,8-tetrabromo-3,6,7-trichlorodibenzo-*p*-dioxin	1	-3739.156	0.609	-0.016	-22.271
1,2,4,8-tetrabromo-3,6,9-trichlorodibenzo-*p*-dioxin	1	-3739.206	-0.765	0.041	-21.087
1,2,4,8-tetrabromo-3,7,9-trichlorodibenzo-*p*-dioxin	1	-3739.127	-0.593	0.018	-20.837
1,2,4,8-tetrabromo-6,7,9-trichlorodibenzo-*p*-dioxin	1	-3739.251	0.073	-0.016	-21.217
1,2,4,9-tetrabromo-3,6,7-trichlorodibenzo-*p*-dioxin	1	-3739.239	0.039	0.011	-21.855
1,2,4,9-tetrabromo-3,6,8-trichlorodibenzo-*p*-dioxin	1	-3739.217	-0.713	0.037	-21.081
1,2,4,9-tetrabromo-3,7,8-trichlorodibenzo-*p*-dioxin	1	-3739.164	-0.527	0.020	-21.009
1,2,4,9-tetrabromo-6,7,8-trichlorodibenzo-*p*-dioxin	1	-3739.293	0.128	-0.016	-21.316
1,2,6,7-tetrabromo-3,4,8-trichlorodibenzo-*p*-dioxin	1	-3739.256	0.573	-0.021	-22.053
1,2,6,7-tetrabromo-3,4,9-trichlorodibenzo-*p*-dioxin	1	-3739.338	-0.051	0.017	-21.818
1,2,6,8-tetrabromo-3,4,7-trichlorodibenzo-*p*-dioxin	1	-3739.160	0.593	-0.021	-22.109
1,2,6,8-tetrabromo-3,4,9-trichlorodibenzo-*p*-dioxin	1	-3739.253	-0.747	0.043	-21.164
1,2,6,8-tetrabromo-3,7,9-trichlorodibenzo-*p*-dioxin	1	-3739.129	-0.552	0.032	-21.279
1,2,6,8-tetrabromo-4,7,9-trichlorodibenzo-*p*-dioxin	1	-3739.214	0.072	-0.011	-21.352
1,2,6,9-tetrabromo-3,4,7-trichlorodibenzo-*p*-dioxin	1	-3739.255	0.050	0.007	-21.769
1,2,6,9-tetrabromo-3,4,8-trichlorodibenzo-*p*-dioxin	1	-3739.255	-0.686	0.039	-21.178
1,2,6,9-tetrabromo-3,7,8-trichlorodibenzo-*p*-dioxin	1	-3739.177	-0.494	0.029	-21.304
1,2,6,9-tetrabromo-4,7,8-trichlorodibenzo-*p*-dioxin	1	-3739.260	0.133	-0.011	-21.461
1,2,7,8-tetrabromo-3,4,6-trichlorodibenzo-*p*-dioxin	1	-3739.255	0.645	-0.021	-22.192
1,2,7,8-tetrabromo-3,4,9-trichlorodibenzo-*p*-dioxin	1	-3739.254	-0.556	0.023	-21.014
1,2,7,8-tetrabromo-3,6,9-trichlorodibenzo-*p*-dioxin	1	-3739.219	-0.507	0.027	-21.219
1,2,7,8-tetrabromo-4,6,9-trichlorodibenzo-*p*-dioxin	1	-3739.302	0.116	-0.014	-21.343
1,2,7,9-tetrabromo-3,4,6-trichlorodibenzo-*p*-dioxin	1	-3739.254	0.064	0.007	-21.797
1,2,7,9-tetrabromo-3,4,8-trichlorodibenzo-*p*-dioxin	1	-3739.160	-0.485	0.022	-21.143
1,2,7,9-tetrabromo-3,6,8-trichlorodibenzo-*p*-dioxin	1	-3739.131	-0.466	0.026	-21.289
1,2,7,9-tetrabromo-4,6,8-trichlorodibenzo-*p*-dioxin	1	-3739.212	0.160	-0.014	-21.445
1,2,8,9-tetrabromo-3,4,6-trichlorodibenzo-*p*-dioxin	1	-3739.338	-0.609	0.033	-21.156
1,2,8,9-tetrabromo-3,4,7-trichlorodibenzo-*p*-dioxin	1	-3739.255	-0.425	0.018	-21.137
1,3,6,8-tetrabromo-2,4,7-trichlorodibenzo-*p*-dioxin	1	-3739.036	0.574	-0.024	-22.018
1,3,6,8-tetrabromo-2,4,9-trichlorodibenzo-*p*-dioxin	1	-3739.129	-0.767	0.045	-21.201
1,3,6,9-tetrabromo-2,4,7-trichlorodibenzo-*p*-dioxin	1	-3739.132	0.028	0.007	-21.750
1,3,6,9-tetrabromo-2,4,8-trichlorodibenzo-*p*-dioxin	1	-3739.130	-0.709	0.041	-21.209
1,3,6,9-tetrabromo-2,7,8-trichlorodibenzo-*p*-dioxin	1	-3739.082	-0.519	0.031	-21.326
1,3,6,9-tetrabromo-4,7,8-trichlorodibenzo-*p*-dioxin	1	-3739.175	0.833	-0.045	-21.947
1,3,7,8-tetrabromo-2,4,6-trichlorodibenzo-*p*-dioxin	1	-3739.129	0.624	-0.023	-22.123
1,3,7,8-tetrabromo-2,4,9-trichlorodibenzo-*p*-dioxin	1	-3739.130	-0.576	0.025	-21.051
1,3,7,8-tetrabromo-2,6,9-trichlorodibenzo-*p*-dioxin	1	-3739.124	-0.530	0.029	-21.244
1,3,7,8-tetrabromo-4,6,9-trichlorodibenzo-*p*-dioxin	1	-3739.218	0.808	-0.050	-21.761
1,3,7,9-tetrabromo-2,4,6-trichlorodibenzo-*p*-dioxin	1	-3739.128	0.039	0.009	-21.824
1,3,7,9-tetrabromo-2,4,8-trichlorodibenzo-*p*-dioxin	1	-3739.035	-0.510	0.022	-21.118

1,4,6,9-tetrabromo-2,3,7-trichlorodibenzo-*p*-dioxin	1	-3739.178	-0.029	0.018	-21.917
1,4,7,8-tetrabromo-2,3,6-trichlorodibenzo-*p*-dioxin	1	-3739.176	0.554	-0.017	-22.135
1,4,7,8-tetrabromo-2,6,9-trichlorodibenzo-*p*-dioxin	1	-3739.219	0.002	-0.013	-21.164
2,3,7,8-tetrabromo-1,4,6-trichlorodibenzo-*p*-dioxin	1	-3739.219	0.580	-0.022	-22.047
1,2,3,4,6-pentabromo-7,8-dichlorodibenzo-*p*-dioxin	1	-3712.783	0.485	-0.015	-27.016
1,2,3,4,6-pentabromo-7,9-dichlorodibenzo-*p*-dioxin	1	-3712.834	-0.117	0.022	-26.804
1,2,3,4,6-pentabromo-8,9-dichlorodibenzo-*p*-dioxin	1	-3712.858	-0.846	0.044	-25.962
1,2,3,4,7-pentabromo-6,8-dichlorodibenzo-*p*-dioxin	1	-3712.748	0.513	-0.013	-27.129
1,2,3,4,7-pentabromo-6,9-dichlorodibenzo-*p*-dioxin	1	-3712.824	-0.113	0.023	-26.840
1,2,3,4,7-pentabromo-8,9-dichlorodibenzo-*p*-dioxin	1	-3712.775	-0.672	0.022	-25.743
1,2,3,6,7-pentabromo-4,8-dichlorodibenzo-*p*-dioxin	1	-3712.747	0.553	-0.022	-26.973
1,2,3,6,7-pentabromo-4,9-dichlorodibenzo-*p*-dioxin	1	-3712.830	-0.068	0.017	-26.770
1,2,3,6,7-pentabromo-8,9-dichlorodibenzo-*p*-dioxin	1	-3712.778	-0.589	0.033	-26.188
1,2,3,6,8-pentabromo-4,7-dichlorodibenzo-*p*-dioxin	1	-3712.652	0.575	-0.021	-27.059
1,2,3,6,8-pentabromo-4,9-dichlorodibenzo-*p*-dioxin	1	-3712.745	-0.761	0.045	-26.174
1,2,3,6,8-pentabromo-7,9-dichlorodibenzo-*p*-dioxin	1	-3712.653	-0.570	0.032	-26.223
1,2,3,6,9-pentabromo-4,7-dichlorodibenzo-*p*-dioxin	1	-3712.747	0.033	0.008	-26.747
1,2,3,6,9-pentabromo-4,8-dichlorodibenzo-*p*-dioxin	1	-3712.746	-0.702	0.040	-26.158
1,2,3,6,9-pentabromo-7,8-dichlorodibenzo-*p*-dioxin	1	-3712.700	-0.513	0.030	-26.272
1,2,3,7,8-pentabromo-4,6-dichlorodibenzo-*p*-dioxin	1	-3712.746	0.626	-0.022	-27.114
1,2,3,7,8-pentabromo-4,9-dichlorodibenzo-*p*-dioxin	1	-3712.746	-0.568	0.024	-26.001
1,2,3,7,8-pentabromo-6,9-dichlorodibenzo-*p*-dioxin	1	-3712.742	-0.525	0.027	-26.163
1,2,3,7,9-pentabromo-4,6-dichlorodibenzo-*p*-dioxin	1	-3712.745	0.046	0.009	-26.798
1,2,3,7,9-pentabromo-4,8-dichlorodibenzo-*p*-dioxin	1	-3712.651	-0.499	0.022	-26.101
1,2,3,7,9-pentabromo-6,8-dichlorodibenzo-*p*-dioxin	1	-3712.653	-0.485	0.028	-26.283
1,2,3,8,9-pentabromo-4,6-dichlorodibenzo-*p*-dioxin	1	-3712.829	-0.623	0.035	-26.165
1,2,3,8,9-pentabromo-4,7-dichlorodibenzo-*p*-dioxin	1	-3712.747	-0.438	0.017	-26.071
1,2,3,8,9-pentabromo-6,7-dichlorodibenzo-*p*-dioxin	1	-3712.778	-0.411	0.022	-26.247
1,2,4,6,7-pentabromo-3,8-dichlorodibenzo-*p*-dioxin	1	-3712.652	0.497	-0.015	-27.064
1,2,4,6,7-pentabromo-3,9-dichlorodibenzo-*p*-dioxin	1	-3712.733	-0.127	0.020	-26.752
1,2,4,6,7-pentabromo-8,9-dichlorodibenzo-*p*-dioxin	1	-3712.776	-0.029	-0.011	-26.128
1,2,4,6,8-pentabromo-3,7-dichlorodibenzo-*p*-dioxin	1	-3712.556	0.521	-0.016	-27.102
1,2,4,6,8-pentabromo-3,9-dichlorodibenzo-*p*-dioxin	1	-3712.649	-0.825	0.045	-26.068
1,2,4,6,8-pentabromo-7,9-dichlorodibenzo-*p*-dioxin	1	-3712.652	-0.006	-0.010	-26.222
1,2,4,6,9-pentabromo-3,7-dichlorodibenzo-*p*-dioxin	1	-3712.652	-0.023	0.012	-26.760
1,2,4,6,9-pentabromo-3,8-dichlorodibenzo-*p*-dioxin	1	-3712.653	-0.766	0.043	-26.130
1,2,4,6,9-pentabromo-7,8-dichlorodibenzo-*p*-dioxin	1	-3712.699	0.054	-0.007	-26.407
1,2,4,7,8-pentabromo-3,6-dichlorodibenzo-*p*-dioxin	1	-3712.651	0.558	-0.015	-27.182
1,2,4,7,8-pentabromo-3,9-dichlorodibenzo-*p*-dioxin	1	-3712.649	-0.637	0.021	-25.808
1,2,4,7,8-pentabromo-6,9-dichlorodibenzo-*p*-dioxin	1	-3712.740	0.025	-0.013	-26.187
1,2,4,7,9-pentabromo-3,6-dichlorodibenzo-*p*-dioxin	1	-3712.650	-0.024	0.015	-26.837
1,2,4,7,9-pentabromo-3,8-dichlorodibenzo-*p*-dioxin	1	-3712.556	-0.568	0.023	-26.011
1,2,4,7,9-pentabromo-6,8-dichlorodibenzo-*p*-dioxin	1	-3712.651	0.068	-0.014	-26.261
1,2,4,8,9-pentabromo-3,6-dichlorodibenzo-*p*-dioxin	1	-3712.734	-0.699	0.036	-26.062

1,2,4,8,9-pentabromo-3,7-dichlorodibenzo-*p*-dioxin	1	-3712.650	-0.504	0.019	-26.013
1,2,4,8,9-pentabromo-6,7-dichlorodibenzo-*p*-dioxin	1	-3712.776	0.144	-0.015	-26.359
1,2,3,4,6,7-hexabromo-8-chlorodibenzo-*p*-dioxin	1	-3686.270	0.479	-0.015	-31.990
1,2,3,4,6,7-hexabromo-9-chlorodibenzo-*p*-dioxin	1	-3686.352	-0.142	0.024	-31.787
1,2,3,4,6,8-hexabromo-7-chlorodibenzo-*p*-dioxin	1	-3686.175	0.503	-0.016	-32.028
1,2,3,4,6,8-hexabromo-9-chlorodibenzo-*p*-dioxin	1	-3686.268	-0.835	0.046	-31.036
1,2,3,4,7,8-hexabromo-6-chlorodibenzo-*p*-dioxin	1	-3686.268	0.541	-0.015	-32.110
1,2,3,6,7,8-hexabromo-4-chlorodibenzo-*p*-dioxin	1	-3686.270	0.571	-0.023	-31.960
1,2,3,6,7,9-hexabromo-4-chlorodibenzo-*p*-dioxin	1	-3686.268	0.011	0.011	-31.761
1,2,3,6,7,9-hexabromo-8-chlorodibenzo-*p*-dioxin	1	-3686.174	-0.515	0.030	-31.256
1,2,3,6,8,9-hexabromo-4-chlorodibenzo-*p*-dioxin	1	-3686.268	-0.693	0.041	-31.180
1,2,3,6,8,9-hexabromo-7-chlorodibenzo-*p*-dioxin	1	-3686.174	-0.498	0.029	-31.263
1,2,3,7,8,9-hexabromo-4-chlorodibenzo-*p*-dioxin	1	-3686.269	-0.481	0.021	-31.070
1,2,4,6,7,9-hexabromo-3-chlorodibenzo-*p*-dioxin	1	-3686.173	-0.047	0.016	-31.797
1,2,4,6,8,9-hexabromo-3-chlorodibenzo-*p*-dioxin	1	-3686.173	-0.756	0.042	-31.102
1,2,3,4,6,7,9-heptabromodibenzo-*p*-dioxin	1	-3659.791	-0.063	0.017	-36.753
1,2,3,4,6,7,8,9-octochlorodibenzo-*p*-dioxin	0	-4086.242	0.000	0.001	-2.315
1-bromo-2,3,4,6,7,8,9-heptachlorodibenzo-*p*-dioxin	0	-4059.651	0.086	-0.004	-7.352
2-bromo-1,3,4,6,7,8,9-heptachlorodibenzo-*p*-dioxin	0	-4059.610	0.061	-0.004	-7.311
1,2-dibromo-3,4,6,7,8,9-hexachlorodibenzo-*p*-dioxin	0	-4033.134	0.098	-0.004	-12.361
1,3-dibromo-2,4,6,7,8,9-hexachlorodibenzo-*p*-dioxin	0	-4033.009	0.076	-0.002	-12.394
1,4-dibromo-2,3,6,7,8,9-hexachlorodibenzo-*p*-dioxin	0	-4033.055	0.002	0.000	-12.294
1,6-dibromo-2,3,4,7,8,9-hexachlorodibenzo-*p*-dioxin	0	-4033.062	0.000	0.002	-12.341
1,8-dibromo-2,3,4,6,7,9-hexachlorodibenzo-*p*-dioxin	0	-4033.020	0.103	-0.001	-12.470
1,9-dibromo-2,3,4,6,7,8-hexachlorodibenzo-*p*-dioxin	0	-4033.062	0.158	-0.005	-12.465
2,3-dibromo-1,4,6,7,8,9-hexachlorodibenzo-*p*-dioxin	0	-4033.099	0.033	-0.001	-12.320
2,7-dibromo-1,3,4,6,8,9-hexachlorodibenzo-*p*-dioxin	0	-4032.980	0.000	0.000	-12.305
2,8-dibromo-1,3,4,6,7,9-hexachlorodibenzo-*p*-dioxin	0	-4032.978	0.077	-0.002	-12.402
1,2,3-tribromo-4,6,7,8,9-pentachlorodibenzo-*p*-dioxin	0	-4006.625	0.082	-0.004	-17.315
1,2,4-tribromo-3,6,7,8,9-pentachlorodibenzo-*p*-dioxin	0	-4006.529	0.011	0.001	-17.326
1,2,6-tribromo-3,4,7,8,9-pentachlorodibenzo-*p*-dioxin	0	-4006.544	0.009	0.000	-17.293
1,2,7-tribromo-3,4,6,8,9-pentachlorodibenzo-*p*-dioxin	0	-4006.503	0.039	-0.001	-17.333
1,2,8-tribromo-3,4,6,7,9-pentachlorodibenzo-*p*-dioxin	0	-4006.503	0.114	-0.003	-17.426
1,2,9-tribromo-3,4,6,7,8-pentachlorodibenzo-*p*-dioxin	0	-4006.545	0.172	-0.007	-17.426
1,3,6-tribromo-2,4,7,8,9-pentachlorodibenzo-*p*-dioxin	0	-4006.420	-0.012	0.002	-17.327
1,3,7-tribromo-2,4,6,8,9-pentachlorodibenzo-*p*-dioxin	0	-4006.377	0.016	-0.002	-17.286
1,3,8-tribromo-2,4,6,7,9-pentachlorodibenzo-*p*-dioxin	0	-4006.379	0.093	-0.001	-17.460
1,3,9-tribromo-2,4,6,7,8-pentachlorodibenzo-*p*-dioxin	0	-4006.420	0.145	-0.007	-17.397
1,4,6-tribromo-2,3,7,8,9-pentachlorodibenzo-*p*-dioxin	0	-4006.467	-0.071	0.004	-17.257
1,4,7-tribromo-2,3,6,8,9-pentachlorodibenzo-*p*-dioxin	0	-4006.424	-0.060	0.005	-17.312
1,7,8-tribromo-2,3,4,6,9-pentachlorodibenzo-*p*-dioxin	0	-4006.509	0.055	-0.001	-17.362
2,3,7-tribromo-1,4,6,8,9-pentachlorodibenzo-*p*-dioxin	0	-4006.467	-0.028	0.002	-17.288
1,2,3,4-tetrabromo-6,7,8,9-tetrachlorodibenzo-*p*-dioxin	0	-3980.147	-0.002	0.000	-22.235
1,2,3,7-tetrabromo-4,6,8,9-tetrachlorodibenzo-*p*-dioxin	0	-3979.994	0.022	-0.001	-22.285

1,2,3,8-tetrabromo-4,6,7,9-tetrachlorodibenzo-*p*-dioxin	0	-3979.995	0.098	-0.002	-22.405
1,2,3,9-tetrabromo-4,6,7,8-tetrachlorodibenzo-*p*-dioxin	0	-3980.036	0.154	-0.007	-22.376
1,2,4,6-tetrabromo-3,7,8,9-tetrachlorodibenzo-*p*-dioxin	0	-3979.941	-0.064	0.003	-22.232
1,2,4,7-tetrabromo-3,6,8,9-tetrachlorodibenzo-*p*-dioxin	0	-3979.898	-0.050	0.003	-22.267
1,2,4,8-tetrabromo-3,6,7,9-tetrachlorodibenzo-*p*-dioxin	0	-3979.897	0.029	0.001	-22.368
1,2,4,9-tetrabromo-3,6,7,8-tetrachlorodibenzo-*p*-dioxin	0	-3979.941	0.085	-0.001	-22.416
1,2,6,7-tetrabromo-3,4,8,9-tetrachlorodibenzo-*p*-dioxin	0	-3980.027	0.000	0.003	-22.340
1,2,6,8-tetrabromo-3,4,7,9-tetrachlorodibenzo-*p*-dioxin	0	-3979.902	0.021	0.002	-22.378
1,2,6,9-tetrabromo-3,4,7,8-tetrachlorodibenzo-*p*-dioxin	0	-3979.950	0.083	-0.002	-22.385
1,2,7,8-tetrabromo-3,4,6,9-tetrachlorodibenzo-*p*-dioxin	0	-3979.992	0.067	-0.002	-22.346
1,2,7,9-tetrabromo-3,4,6,8-tetrachlorodibenzo-*p*-dioxin	0	-3979.903	0.112	-0.003	-22.423
1,2,8,9-tetrabromo-3,4,6,7-tetrachlorodibenzo-*p*-dioxin	0	-3980.028	0.185	-0.005	-22.489
1,3,6,8-tetrabromo-2,4,7,9-tetrachlorodibenzo-*p*-dioxin	0	-3979.780	0.000	0.003	-22.386
1,3,6,9-tetrabromo-2,4,7,8-tetrachlorodibenzo-*p*-dioxin	0	-3979.826	0.057	-0.001	-22.384
1,3,7,8-tetrabromo-2,4,6,9-tetrachlorodibenzo-*p*-dioxin	0	-3979.867	0.044	-0.002	-22.325
1,3,7,9-tetrabromo-2,4,6,8-tetrachlorodibenzo-*p*-dioxin	0	-3979.777	0.085	-0.003	-22.395
1,4,6,9-tetrabromo-2,3,7,8-tetrachlorodibenzo-*p*-dioxin	0	-3979.874	0.000	0.002	-22.342
1,4,7,8-tetrabromo-2,3,6,9-tetrachlorodibenzo-*p*-dioxin	0	-3979.913	-0.029	0.002	-22.279
2,3,7,8-tetrabromo-1,4,6,9-tetrachlorodibenzo-*p*-dioxin	0	-3979.956	0.000	0.002	-22.327
1,2,3,4,6-pentabromo-7,8,9-trichlorodibenzo-*p*-dioxin	0	-3953.559	-0.079	0.005	-27.216
1,2,3,4,7-pentabromo-6,8,9-trichlorodibenzo-*p*-dioxin	0	-3953.516	-0.064	0.003	-27.201
1,2,3,6,7-pentabromo-4,8,9-trichlorodibenzo-*p*-dioxin	0	-3953.519	-0.017	0.001	-27.239
1,2,3,6,8-pentabromo-4,7,9-trichlorodibenzo-*p*-dioxin	0	-3953.395	0.005	0.001	-27.305
1,2,3,6,9-pentabromo-4,7,8-trichlorodibenzo-*p*-dioxin	0	-3953.442	0.065	-0.003	-27.308
1,2,3,7,8-pentabromo-4,6,9-trichlorodibenzo-*p*-dioxin	0	-3953.483	0.051	-0.001	-27.326
1,2,3,7,9-pentabromo-4,6,8-trichlorodibenzo-*p*-dioxin	0	-3953.394	0.094	-0.003	-27.373
1,2,3,8,9-pentabromo-4,6,7-trichlorodibenzo-*p*-dioxin	0	-3953.519	0.168	-0.005	-27.441
1,2,4,6,7-pentabromo-3,8,9-trichlorodibenzo-*p*-dioxin	0	-3953.424	-0.077	0.003	-27.193
1,2,4,6,8-pentabromo-3,7,9-trichlorodibenzo-*p*-dioxin	0	-3953.298	-0.050	0.004	-27.295
1,2,4,6,9-pentabromo-3,7,8-trichlorodibenzo-*p*-dioxin	0	-3953.348	0.010	0.003	-27.376
1,2,4,7,8-pentabromo-3,6,9-trichlorodibenzo-*p*-dioxin	0	-3953.387	-0.019	0.004	-27.338
1,2,4,7,9-pentabromo-3,6,8-trichlorodibenzo-*p*-dioxin	0	-3953.300	0.023	-0.001	-27.306
1,2,4,8,9-pentabromo-3,6,7-trichlorodibenzo-*p*-dioxin	0	-3953.423	0.100	-0.001	-27.431
1,2,3,4,6,7-hexabromo-8,9-dichlorodibenzo-*p*-dioxin	0	-3927.041	-0.090	0.004	-32.155
1,2,3,4,6,9-hexabromo-7,8-dichlorodibenzo-*p*-dioxin	0	-3926.966	-0.007	0.002	-32.278
1,2,3,4,7,8-hexabromo-6,9-dichlorodibenzo-*p*-dioxin	0	-3927.005	-0.033	0.002	-32.220
1,2,3,6,7,8-hexabromo-4,9-dichlorodibenzo-*p*-dioxin	0	-3927.010	0.000	0.001	-32.257
1,2,3,6,7,9-hexabromo-4,8-dichlorodibenzo-*p*-dioxin	0	-3926.915	0.058	-0.002	-32.309
1,2,3,6,8,9-hexabromo-4,7-dichlorodibenzo-*p*-dioxin	0	-3926.915	0.076	-0.003	-32.318
1,2,3,7,8,9-hexabromo-4,6-dichlorodibenzo-*p*-dioxin	0	-3927.010	0.117	-0.005	-32.327
1,2,4,6,7,9-hexabromo-3,8-dichlorodibenzo-*p*-dioxin	0	-3926.821	0.000	0.001	-32.292
1,2,4,6,8,9-hexabromo-3,7-dichlorodibenzo-*p*-dioxin	0	-3926.820	0.023	0.000	-32.311
1,2,3,4,6,7,8-heptabromo-9-chlorodibenzo-*p*-dioxin	0	-3900.533	-0.072	0.006	-37.226
1,2,3,4,6,7,9-heptabromo-8-chlorodibenzo-*p*-dioxin	0	-3900.439	-0.016	0.001	-37.222

References:

1. Haranczyk, M.; Urbaszek, P.; Ng, E.G.; Puzyn, T. *J Chem Inf Model* **2012**, *52*, 2902-2909.
2. Stewart, J.J. *Journal of computer-aided molecular design* **1990**, *4*, 1-105.
3. Puzyn, T.; Suzuki, N.; Haranczyk, M.; Rak, J. *J Chem Inf Model* **2008**, *48*, 1174-1180.
4. Kennard, R.W.; Stone, L.A. *Technometrics* **1969**, *11*, 137-&.
5. Hanwell, M.D.; Curtis, D.E.; Lonie, D.C.; Vandermeersch, T.; Zurek, E.; Hutchison, G.R. *Journal of cheminformatics* **2012**, *4*, 17.
6. Hlawacek, G.; Khokhar, F.S.; van Gastel, R.; Poelsema, B.; Teichert, C. *Nano letters* **2011**, *11*, 333-337.
7. Hassinen, T.; Perakyla, M. *J Comput Chem* **2001**, *22*, 1229-1242.
8. Zhao, Y.; Truhlar, D.G.. *Theor Chem Acc* **2008**, *120*, 215-241.
9. Scott, A.M.; Gorb, L.; Mobley, E.A.; Hill, F.C.; Leszczynski, J. *Langmuir* **2012**, *28*, 13307-13317.
10. Michalkova, A.; Gorb, L.; Hill, F.; Leszczynski, J. *J Phys Chem A* **2011**, *115*, 2423-2430.
11. Gu, J.D.; Wang, J.; Leszczynski, J.; Xie, Y.M.; Schaefer, H.F. *Chem Phys Lett* **2008**, *459*, 164-166.
12. Haranczyk, M.; Puzyn, T.; Sadowski, P. *Qsar Comb Sci* **2008**, *27*, 826-833.
13. OECD. *Oecd principles for the validation, for regulatory purposes, of (q)sar models*; Organisation of Economic Cooperation and Development: Paris, France, 2004.
14. Hewitt, M.; Cronin, M.T.D.; Madden, J.C.; Rowe, P.H.; Johnson, C.; Obi, A.; Enoch, S.J. *J Chem Inf Model* **2003**, *47*, 1460-1468
